# High-throughput screening and rational design of biofunctionalized surfaces with optimized biocompatibility and antimicrobial activity

**DOI:** 10.1038/s41467-021-23954-8

**Published:** 2021-06-18

**Authors:** Zhou Fang, Junjian Chen, Ye Zhu, Guansong Hu, Haoqian Xin, Kunzhong Guo, Qingtao Li, Liangxu Xie, Lin Wang, Xuetao Shi, Yingjun Wang, Chuanbin Mao

**Affiliations:** 1 National Engineering Research Center for Tissue Restoration and Reconstruction, Higher Education Mega Center, South China University of Technology, Panyu, Guangzhou China; 2 School of Materials Science & Engineering, Higher Education Mega Center, South China University of Technology, Panyu, Guangzhou China; 3 School of Biomedical Science and Engineering, Higher Education Mega Center, South China University of Technology, Panyu, Guangzhou China; 4 Department of Chemistry and Biochemistry, Stephenson Life Sciences Research Center, University of Oklahoma, Norman, OK USA; 5 Institute of Bioinformatics and Medical Engineering, Jiangsu University of Technology, Changzhou, China; 6 Bioland Laboratory (Guangzhou Regenerative Medicine and Health Guangdong Laboratory), Guangzhou, China; 7 School of Materials Science & Engineering, Zhejiang University, Hangzhou, China

**Keywords:** Antimicrobials, Biomaterials, Implants

## Abstract

Peptides are widely used for surface modification to develop improved implants, such as cell adhesion RGD peptide and antimicrobial peptide (AMP). However, it is a daunting challenge to identify an optimized condition with the two peptides showing their intended activities and the parameters for reaching such a condition. Herein, we develop a high-throughput strategy, preparing titanium (Ti) surfaces with a gradient in peptide density by click reaction as a platform, to screen the positions with desired functions. Such positions are corresponding to optimized molecular parameters (peptide densities/ratios) and associated preparation parameters (reaction times/reactant concentrations). These parameters are then extracted to prepare nongradient mono- and dual-peptide functionalized Ti surfaces with desired biocompatibility or/and antimicrobial activity in vitro and in vivo. We also demonstrate this strategy could be extended to other materials. Here, we show that the high-throughput versatile strategy holds great promise for rational design and preparation of functional biomaterial surfaces.

## Abstract (web summary)

Optimizing the concentration of different functional peptides on a surface can be a complex process. Here, the authors report on the use of a click immobilization strategy to create gradients of two different functional peptides on a surface to screen different density functions for rapid optimization.

## Introduction

In the clinic, a generation of biomaterials with improved biofunctions is urgently required^1–4^. As the interface between biomaterials and tissues is crucial^5^, biofunctionalization of the surfaces, i.e., improvement of the composition^6^, topography^7,8^, or hydrophobicity^9^ of the surfaces, is considered as a suitable strategy for the design of biomaterials. One approach to surface biofunctionalization is the display of peptides on biomaterials^10,11^. The peptides can be integrated onto biomaterial surfaces via physical adsorption^12–14^ or covalent immobilization^15–17^ to stimulate specific bioactivities, including biocompatibility^18^, hemocompatibility^19^, antimicrobial activity^20^, osteogenic activity^21^, or angiogenic activity^22^. Furthermore, more than one peptide can be used to functionalize the same surface of a biomaterial^11,23^.

Regardless of mono- or dual-peptide functionalization, the density of peptides on the surface often plays a critical role in the biofunction^24–26^. We^27,28^, along with others^29–31^, have revealed that low peptide densities can cause insufficient activities, while high peptide densities can generate undesired side effects. For example, antimicrobial peptide (AMP) has been utilized on biomaterial surface to inhibit bacterial infection^32,33^, but excessive AMP on the surface leads to cytotoxicity and inhibits tissue healing^27,34^. In addition, nonoptimized peptide densities on a dual-peptide functionalized surface could also sacrifice the biofunctions from one of the two peptides^35–37^. Therefore, it is of great importance to develop a method for the rational design of the optimized peptide densities, in particular, for dual-peptide functionalization.

However, to the best of our knowledge, there is still a lack of an efficient strategy for rational design and preparation of the biofunctionalized surfaces. As a traditional technique, orthogonal testing is time consuming, requiring the preparation of many surfaces for optimizing the parameters^11,38^. In some cases, microfluidic techniques were powerful to simplify the optimization process and obtain best conditions^39–41^. However, the systems were complicated to build. Still, it was not practical to apply them on some widely used biomaterials, e.g., titanium (Ti), directly to obtain the optimized preparation parameters for preparing biofunctionalized surfaces^42–44^.

A gradient surface can be used to collect a large amount of data by continuously varying the parameters in a single surface. Such surface can be employed to reveal the interactions between the cells and the surface properties^45–55^, e.g., surface topography^45–47^, the densities of biomolecules^48–51^, and surface compositions^52–55^. Unfortunately, until now, using a gradient surface to rationally design the optimized implants was still unrevealed, and almost no studies have been dedicated to the use of it as a platform for high-throughput screening of the optimized parameters for rational design and preparation of biofunctionalized surfaces.

**Fig. 1:**
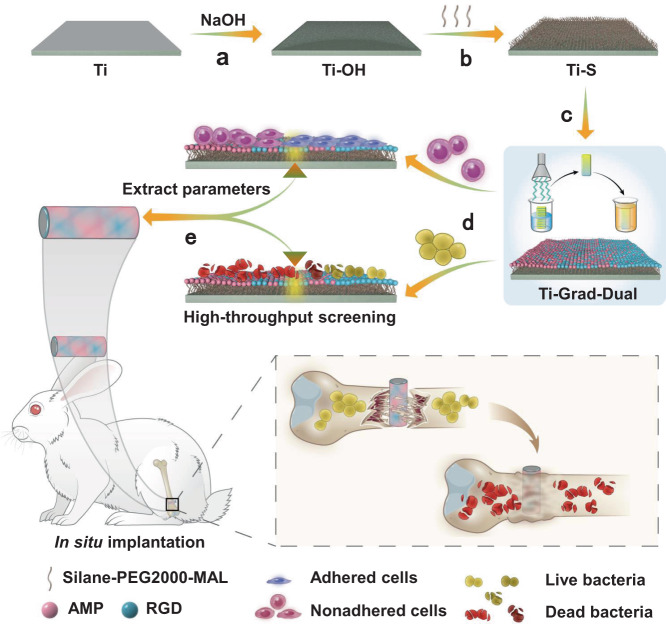
Schematic illustration of a platform for high-throughput screening and rational design of the biofunctionalized surfaces with optimized biocompatibility and antimicrobial activity. **a** Ti substrates were treated with sodium hydroxide to realize hydroxylation. **b** Hydroxylated Ti substrates were reacted with silane-PEG2000-MAL solution to introduce alkene bonds. **c** By the thiol–ene click chemistry, utilizing a combination of “evaporation” and “immersion” techniques to construct AMP and RGD dual-functionalized gradient surfaces. **d** Studying the biological properties of *mBMSCs* and *S. aureus* on the AMP and RGD dual-functionalized gradient surfaces, to identify the best region which can promote both cell adhesion and bacteria killing and thus to obtain the best parameters for producing the optimized AMP and RGD dual-functionalized surfaces. **e** The optimized AMP and RGD dual-functionalized surfaces were designed by the extracted parameters to achieve excellent in vitro and in vivo biocompatibility and antimicrobial activity for successful BAI inhibition and bone defect repair.

To tackle this challenge, we develop a high-throughput strategy based on the gradient surface for rational design and preparation of a mono- or dual-peptide functionalized biomaterial surface, in particular, obtaining the optimized peptide densities/ratios and preparation parameters (Fig. 1). We choose Ti as a substrate because it is an important biomaterial widely used in the clinic for orthopedic and cardiovascular treatments^56–58^. We employ RGD peptide (abbreviated as RGD, Arg-Gly-Asp, improving biocompatibility^59,60^) and HHC36 peptide (abbreviated as AMP, Lys-Arg-Trp-Trp-Lys-Trp-Trp-Arg-Arg, improving antimicrobial activity^61^) as two model peptides. The two peptides bear a sequence of Cys-Pro-Ala-Pro-Ala-Pro at the N-terminus as a rigid spacer to improve their orientation and bioactivity^62,63^. Then, we introduce an alkene group onto the Ti surface via a silane coupling agent, and form mono- or dual-peptide functionalized surface by the thiol–ene click chemistry^64–67^ with a gradient in the peptide density via the “titration” or “evaporation” technique.

Here, we show the gradient surface with mono or dual peptides can be generated on the Ti surfaces. We further confirm that the resultant gradient surface can be used as a high-throughput platform to discover the optimized parameters for forming a specific peptide density that can achieve best biocompatibility and excellent antimicrobial activity both in vitro and in vivo. Finally, we demonstrate that our strategy can be extended to generate gradient surfaces on other materials such as gold.

## Results

### The RGD-functionalized Ti surface

**Fig. 2:**
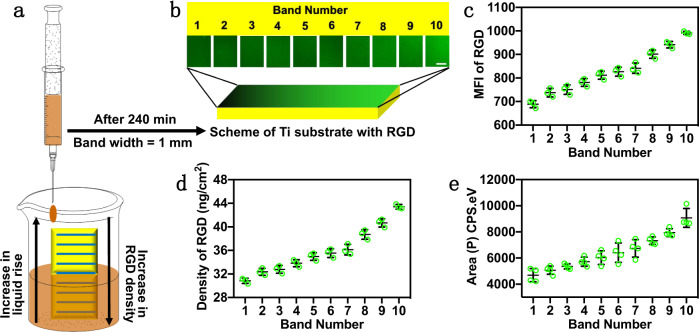
The preparation and gradient properties of Ti surfaces with gradient RGD density along the vertical direction. **a** Schematic diagram depicting the preparation of the RGD-functionalized gradient Ti surface. A Ti substrate, chemically modified with silane-PEG2000-MAL, was first vertically oriented in an empty well of a plate. Then, an FITC-labeled thiolated RGD solution with a concentration of *m* (*m* = 0.1, 0.5, or 1 μM) was added to the well at a rate of 0.5 mL/h, leading to the increase of liquid level in the well. RGD was conjugated to the freshly immersed section of the Ti substrate by a click reaction between double bonds in MAL and thiol groups in RGD. After 240 min, the Ti substrate was pulled out of the well and washed with ethanol. Consequently, a lower section of the Ti substrate would have been exposed to the RGD solution for a longer time and thus be modified with more RGD, making RGD density increasing vertically downward and generating a vertical gradient in the RGD density. The resultant gradient surface was denoted as Ti-Grad-*m*RGD, in which *m* represents the concentration of the RGD solution. For convenience, the surface was artificially divided into ten bands, numbered 1–10 from top to bottom, with a band width of 1 mm. Thus, it is expected that the RGD density is increasing from band 1 to 10. **b** The FITC fluorescence images of Ti-Grad-0.5RGD. The images of the ten bands were collected individually and lined up as they were originally on the Ti substrate (*n* = 3, scale bar, 200 μm). **c** The mean fluorescence intensity (MFI) and **d** the RGD density (calculated by the fluorescence method^11^) of each band of Ti-Grad-0.5RGD. In each band, we randomly selected three points to calculate the MFI (*n* = 3). **e** Peak area of N1s high-resolution spectra of each band on Ti-Grad-0.5RGD, confirming the increase in the RGD density from band 1 to 10 (*n* = 4). Data are displayed as mean ± SD and analyzed by GraphPad Prism software.

All peptides with given sequences (Supplementary Table 1) were synthesized with or without a fluorescent tag (FITC or Mca). Before the preparation of the gradient surface, Ti was modified with silane-PEG2000-MAL (Ti–S). The results of XPS, AFM, and FTIR (Supplementary Figs. 1–3) demonstrated that the silanization was homogeneous on Ti–S. The CCK-8 and antimicrobial results showed that Ti–S had similar biocompatibility and antimicrobial activity as the pristine Ti (Supplementary Fig. 4). Subsequently, we developed a “titration” method to prepare a gradient surface on Ti–S (Fig. 2a). Ti–S was first vertically oriented in an empty well. Then, a thiolated RGD–FITC solution with a concentration of *m* (*m* = 0.1, 0.5, or 1 μM) was added to the well at a rate of 0.5 mL/h, elevating the liquid level in the well. RGD was conjugated to the freshly immersed section of the Ti–S by a thiol–ene click reaction between double bonds in maleimide (MAL) and thiol groups in RGD. It takes at least 2 h to complete a thiol–ene click reaction on a materials surface^67^. Thus, we set the “titration” time as 4 h to ensure the click reaction is completed for the area immersed in the solution for the longest time. After 4 h, the Ti substrate was pulled out of the well and washed with ethanol. For convenience, the Ti surface was artificially divided into ten bands, numbered 1–10 from top to bottom, with a band width of 1 mm. Thus, the higher number a given band belonged to, the longer the immersion time (and the click reaction time) that band experienced, and the higher the RGD density that band would have. Namely, the RGD density was expected to be increasing from band 1 to 10. The resultant gradient surface was denoted as Ti-Grad-*m*RGD, in which *m* represents the concentration of the RGD solution.

The fluorescent peptides were often employed to quantify the grafting density of the peptides on the surface^11^. Herein, we also used the FITC-labeled peptides, i.e., RGD–FITC and AMP–FITC, to calculate the grafting density of the specific peptide. The all-atom molecular dynamic (MD) simulation results showed that the peptides with and without FITC had similar radius of gyration (Supplementary Fig. 5), demonstrating that the introduction of FITC had a negligible effect on the steric hindrance. FITC fluorescence imaging of different bands (Fig. 2b and Supplementary Fig. 6) showed that the mean fluorescence intensities (MFI) of Ti-Grad-0.5RGD increased from 688.6 ± 14.4 to 991.4 ± 7.0 a.u. from band 1 to band 10 (Fig. 2c). By establishing a standard curve following the reported fluorescence method^11^ (Supplementary Fig. 7), we found that there was an inverse linear relation between MFI and the reciprocal of the RGD–FITC density. Thus, we determined that the RGD density increased from 31.1 ± 0.4 ng/cm^2^ (one molecule in 4.8 nm^2^) in band 1 to 43.6 ± 0.4 ng/cm^2^ (one molecule in 3.4 nm^2^) in band 10 on Ti-Grad-0.5RGD (Fig. 2d). The surface XPS N1s intensity from each band also exhibited that a higher band number was corresponding to a higher N content (and thus a higher RGD density) (Fig. 2e and Supplementary Fig. 8). Meanwhile, the FTIR results showed that the intensities of carbonyl groups in amide I increased from band 1 to band 10, further demonstrating the gradient distribution of RGD (Supplementary Fig. 9).

**Fig. 3:**
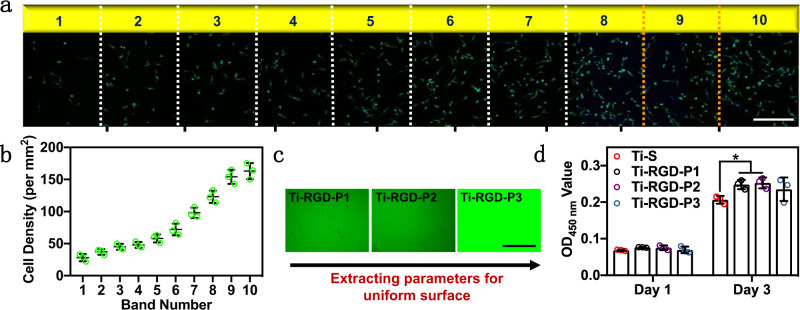
Correlation between the *mBMSCs* density and the band-dependent RGD density on the gradient Ti surfaces and the use of information from such correlation to prepare nongradient surfaces for optimized *mBMSCs* adhesion. **a** Distribution (*n* = 3) and **b** average number (*n* = 3 within a band) of fluorescently stained *mBMSCs* on different bands of Ti-Grad-0.5RGD after 24 h of culturing, showing an increase in the band number (and thus an increase in RGD density) resulted in an increase in the cell density. After being stained with F-actin and DAPI, the cells were observed by fluorescence microscope under the FITC and DAPI channels (scale bar, 400 μm). It should be noted that 9 images were obtained along the gradient direction of one sample and combined to form the image in **a**, and the dotted lines in **a** highlight the band boundaries (but not the dividing lines of the 9 images). The actual dividing boundaries of the 9 images are marked as solid black lines at the bottom of the image in **a**. **c** FITC fluorescence images of nongradient Ti surface prepared from the incubation time (in FITC-labeled RGD solution) used to generate bands 9 and 10, producing Ti-RGD-P1 and Ti-RGD-P2, respectively. A third surface, Ti-RGD-P3, was produced by increasing the RGD concentration to 1 μM for Ti-RGD-P2. For each sample, five images were chosen randomly to calculate the MFI (*n* = 5, scale bar, 500 μm). **d** CCK-8 results for the indicated uniform Ti surface with *mBMSCs* after 1 and 3 days of culturing (*n* = 3). (Sidak’s multiple comparisons test, two-way ANOVA. Ti–S vs Ti-RGD-P1, *p = 0.0119; Ti–S vs Ti-RGD-P2, *p = 0.0118.) Ti–S was the Ti surface modified with silane-PEG2000-MAL. Data are displayed as mean ± SD and analyzed by GraphPad Prism software.

As expected, the gradient in the RGD density resulted in a gradient distribution in the cell density and the spreading area of the cells on Ti-Grad-0.5RGD by imaging the fluorescently stained mouse bone marrow derived mesenchymal stem cells (*mBMSCs*) (Fig. 3a). That is, the cell density increased from 28.0 ± 6.0 cell/mm^2^ for band 1 to 163.0 ± 12.0 cell/mm^2^ for band 10 (Fig. 3b), and the spreading area of the cells increased from 1469.5 ± 75.8 μm^2^ for band 1 to 1976.9 ± 49.2 μm^2^ for band 10 (Supplementary Fig. 10). Bands 9 and 10, with high cell densities of 154.0 ± 11.0 and 163.0 ± 12.0 cell/mm^2^, and a large cell spreading area of 1938.1 ± 40.2 and 1976.9 ± 49.2 μm^2^, were corresponding to a RGD density of 41.4 ± 0. and 43.6 ± 0.4 ng/cm^2^, as well as an immersion/reaction time of 216 and 240 min (the terminal boundary of the band), respectively (Fig. 3c). Although both Ti-Grad-0.1RGD and Ti-Grad-1RGD also presented a gradient distribution of RGD density (Supplementary Figs. 11 and 12), both surfaces did not show the corresponding gradient distribution of cell density and cell spreading area (Supplementary Figs. 13 and 14), probably due to the insufficient or excessive RGD on the surfaces. Interestingly, we found that the cell spreading area on Ti-Grad-0.1RGD (1527.6 μm^2^) was smaller than that on Ti-Grad-1RGD (1821.8 μm^2^). When the RGD densities of the bands on these surfaces were similar to the specific bands on Ti-Grad-0.5RGD, their cell spreading area was similar as well. For instance, when the MFI of band 10 on Ti-Grad-0.5RGD was 991.4 a.u., and the MFI of band 1 on Ti-Grad-1RGD was 1085.0 a.u., the cell spreading area in these two bands were 1976.9 and 1821.3 μm^2^, respectively, though these bands were on the different surfaces.

Based on the parameters extracted from bands 9 and 10 on Ti-Grad-0.5RGD, we prepared uniform Ti surfaces of Ti-RGD-P1 and Ti-RGD-P2 by immersing Ti–S in 0.5 μM of RGD or RGD–FITC solution for 216 and 240 min, respectively. Both Ti-RGD-P1 and Ti-RGD-P2 presented high fluorescence due to the high RGD–FITC density (Fig. 3c). According to the standard curve (Supplementary Fig. 7) and the MFI of both surfaces (954.5 ± 21.2 and 1000.9 ± 71.4 a.u. for Ti-RGD-P1 and Ti-RGD-P2, respectively), we found that these two surfaces had similar densities of RGD (41.4 ± 1.1 and 44.3 ± 4.4 ng/cm^2^, respectively) as bands 9 and 10 on Ti-Grad-0.5RGD, demonstrating that the conditions used for producing a particular band extracted from the gradient surface study could be effectively used to produce nongradient surface with the same properties as that band. Further, CCK-8 results revealed that compared to that of Ti–S, the biocompatibilities of Ti-RGD-P1 and Ti-RGD-P2 were improved 1.2- and 1.2-folds after 3 days (Fig. 3d). We also prepared Ti-RGD-P3 surface with 1 μM of RGD–FITC solution and 240 min of incubation. This surface had an increased MFI of 3045.5 ± 30.2 a.u. (Fig. 3c), demonstrating the larger density of RGD. However, Ti-RGD-P3 exhibited a little lower biocompatibility than Ti-RGD-P1 and Ti-RGD-P2, which was 1.1-fold that of Ti–S (Fig. 3d). This finding was consistent with our results (Supplementary Fig. 14) and other reports^50^ that an excessive RGD density had a negligible or even negative effect on improving the cell viability. The above results suggested that the biocompatibility of the surface could be optimized when the densities of RGD were ~41.4–44.3 ng/cm^2^ on Ti. Moreover, we could obtain the optimized preparation parameters (0.5 μM of RGD solution and 216–240 min of reaction time) for preparing such surfaces from the gradient RGD-functionalized Ti surface.

### The AMP-functionalized Ti surface

**Fig. 4:**
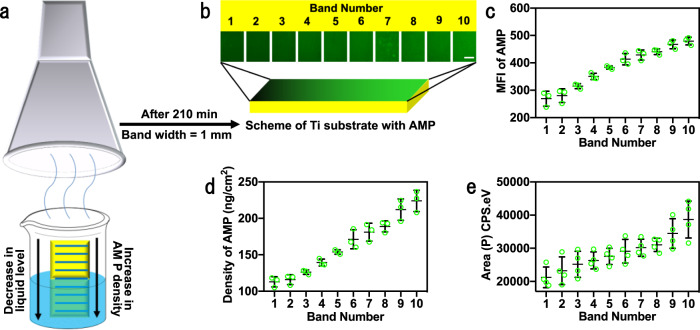
The preparation and gradient properties of the Ti surface with a gradient in AMP density along the vertical direction. **a** Schematic diagram depicting the preparation of the AMP-functionalized gradient Ti surface. A Ti substrate, chemically modified with silane-PEG2000-MAL, was first vertically oriented in an FITC-labeled thiolated AMP solution with a concentration of *m* (*m* = 1, 20, or 50 μM). Then, the solution phase was allowed to evaporate in a fume hood for 210 min. AMP was conjugated to the immersed section of the Ti substrate by a click reaction between double bonds in MAL and thiol groups in thiolated AMP. During the evaporation, the solution level was reduced to increase the concentration of the solution phase. As a result, a lower section of the Ti substrate would have been exposed to the AMP solution with a higher concentration and for a longer time and thus be modified with more AMP, making AMP density increasing vertically downward and generating a vertical gradient in the AMP density. The resultant gradient surface was denoted as Ti-Grad-mAMP, in which *m* represents the concentration of the AMP solution. For convenience, the surface was artificially divided into ten bands, numbered 1–10 from top to bottom, with a band width of 1 mm. Thus, it is expected that the AMP density is increasing from band 1 to 10. **b** The FITC fluorescence images of Ti-Grad-20AMP. The images of the ten bands were collected individually and lined up as they were originally on the Ti substrate (*n* = 3, scale bar, 200 μm). **c** The MFI and **d** the AMP density (calculated by the fluorescence method^11^) of each band of Ti-Grad-20AMP. In each band, we randomly selected three points to calculate the MFI (*n* = 3). **e** Peak area of N1s high-resolution spectra of each band on Ti-Grad-20AMP, confirming the increase in the AMP density from band 1 to 10 (*n* = 4). Data are displayed as mean ± SD and analyzed by GraphPad Prism software.

We also prepared the AMP-functionalized gradient surface on Ti–S based on the “evaporation” technique (Fig. 4a). By this method, the reaction time and the reactant concentration were also changed in different bands during the evaporation of the AMP–FITC solution (with Ti–S vertically oriented in the well) for 210 min. During the evaporation, the solution level was reduced to increase the concentration of the solution phase. As a result, a lower section of the Ti substrate with a larger band number would have been exposed to the AMP solution with a higher concentration and for a longer time and thus be modified with more AMP, making the AMP density increasing vertically from band 1 to 10. The resultant gradient surface was denoted as Ti-Grad-mAMP, in which *m* represents the concentration of the AMP solution.

Indeed, AMP–FITC had a gradient distribution on Ti-Grad-20AMP (Fig. 4b and Supplementary Fig. 15) with the MFI increasing from 269.2 ± 27.2 a.u. in band 1 to 479.6 ± 14.3 a.u. in band 10 (Fig. 4c), demonstrating successful preparation of the gradient surface. We also found that there was an inverse linear relation between MFI and the reciprocal of the AMP–FITC density (Supplementary Fig. 16). This standard curve allowed us to determine that the AMP density increased from 113.0 ± 7.0 ng/cm^2^ (one molecule in 3.0 nm^2^) in band 1 to 223.9 ± 14.6 ng/cm^2^ (one molecule in 1.5 nm^2^) in band 10 on Ti-Grad-20AMP (Fig. 4d). Moreover, the XPS N1s and the FTIR results revealed a similar trend in the distribution of AMP (Fig. 4e and Supplementary Figs. 17 and 18). In addition, the XPS N1s and the FTIR results of Ti-AMP-Control (Ti–S treated with AMP without thiol group) also showed that there were negligible physically adsorbed peptides on the surface after cleaning (Supplementary Fig. 19). Interestingly, although AMP had a higher grafting density than RGD, Ti-Grad-AMP displayed much lower MFI than Ti-Grad-RGD. Similarly, MFI of AMP–FITC was also lower than that of RGD–FITC in the peptide solution (Supplementary Fig. 20a). The MD simulation results showed that it resulted from the difference in the solvent accessible surface area (SASA) of FITC between these two peptides; the FITC in RGD–FITC had a larger SASA to be exposed than that in AMP–FITC and thus showed stronger fluorescence (Supplementary Fig. 20b, c).

**Fig. 5:**
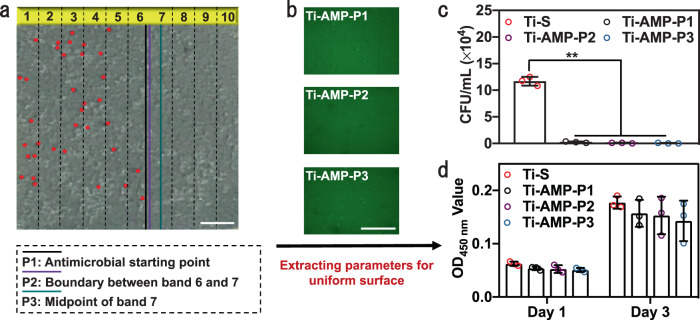
Correlation between the number of live bacteria (*S. aureus*) and the band-dependent AMP density on the gradient Ti surfaces and the use of information from such correlation to prepare nongradient surfaces for optimized bacteria killing. **a** Distribution of live bacteria (red dots, detected by Petrifilm method) on different bands of Ti-Grad-20AMP after 24 h of culturing, showing that higher band number area (bands 7–10, with higher AMP densities) did not have any live bacteria, whereas lower band number area (bands 1–6, with lower AMP densities) presented live bacteria. Three lines were denoted, including P1, P2, and P3, corresponding to the boundary between the area without and with dead bacteria, starting boundary of band 7, and midpoint position within band 7, respectively (*n* = 3, scale bar, 1.5 mm). **b** FITC fluorescence images of the nongradient Ti surface prepared using the conditions (immersion time and AMP concentration during immersion) corresponding to P1, P2, and P3 in **a**, producing Ti-AMP-P1, Ti-AMP-P2, and Ti-AMP-P3, respectively. For each sample, five images were chosen randomly to calculate the MFI (*n* = 5, scale bar, 500 μm). **c** Bacterial viability of Ti–S, AMP-P1, Ti-AMP-P2, and Ti-AMP-P3 (*n* = 3) (Sidak’s multiple comparisons test, two-way ANOVA. Ti–S vs Ti-AMP-P1, **p < 0.0001; Ti–S vs Ti-AMP-P2, **p < 0.0001; Ti–S vs Ti-AMP-P3, **p < 0.0001). **d** CCK-8 results for the indicated uniform Ti surface with *mBMSCs* after 1 and 3 days of culturing (*n* = 3). Ti–S was the Ti surface modified with silane-PEG2000-MAL. Data are displayed as mean ± SD and analyzed by GraphPad Prism software.

The antimicrobial activity of Ti-Grad-20AMP was characterized by the Petrifilm method. The surface started to exhibit antimicrobial activity from the point denoted with the black line in band 6, which was set as P1 (antimicrobial starting point; 48.3 μM of AMP for 123.1 min) (Fig. 5a). Moreover, we found that the gradient surface exhibited excellent antimicrobial activity (without live bacteria being detected) from band 7 to 10 but poor antimicrobial activity (with live bacteria being detected) from band 1 to 6 (Fig. 5a). Consequently, we denoted the position with the purple line as P2 (the boundary between bands 6 and 7; 50.0 μM of AMP for 126.0 min) and that with the cyan line as P3 (midpoint of band 7; 57.1 μM of AMP for 136.5 min). According to the distribution of AMP (Fig. 4d), the AMP density at P1 was ~171.2 ± 13.1 ng/cm^2^ (same as that of band 6), and the densities of AMP at P2 and P3 were ~181.0 ± 12.2 ng/cm^2^ (same as that of band 7). In addition, we found that there was no gradient distribution of AMP on Ti-Grad-1AMP (Supplementary Fig. 21), which should be caused by the insufficient initial density of AMP. Consequently, the surfaces of Ti–S and Ti-Grad-1AMP did not exhibited gradient antimicrobial activity due to the lack of AMP, and the bacteria distributed uniformly on the surfaces (Supplementary Fig. 22).

Based on the preparation parameters obtained from the three positions (P1, P2, and P3) on Ti-Grad-20AMP, we prepared the nongradient surfaces and denoted these surfaces as Ti-AMP-P1, Ti-AMP-P2, and Ti-AMP-P3. The MFI of these three surfaces were 403.1 ± 9.1, 412.5 ± 21.4, and 426.6 ± 24.3 a.u. (Fig. 5b). According to the standard curve (Supplementary Fig. 16), the AMP densities on these three surfaces were determined to be 164.7 ± 5.1, 170.8 ± 12.8, and 180.2 ± 17.5 ng/cm^2^, respectively, which were similar to the densities of the specific sites (P1, P2, and P3) on Ti-Grad-20AMP. Furthermore, the antimicrobial assays illustrated that Ti-AMP-P1, Ti-AMP-P2, and Ti-AMP-P3 exhibited an efficiency of 98.0, 99.0, and 99.7% in the inhibition of *S. aureus*, respectively, compared to Ti–S (Fig. 5c).

Excellent antimicrobial activity for P1, P2, and P3 positions on gradient surfaces as well as the nongradient surfaces of their counterparts suggested that the optimized AMP densities were ~164.7–180.2 ng/cm^2^ on Ti implant. The parameters for producing such nongradient surfaces, which were derived from the gradient surface study, are to immerse Ti–S into an AMP solution (48.3–57.1 μM) for a given time (123.1–136.5 min, respectively). Unfortunately, besides the antimicrobial activity, these nongradient surfaces exhibited evident cytotoxicity to *mBMSCs* (Fig. 5d). Compared to Ti–S, the biocompatibilities of Ti-AMP-P3 were only 81.3 and 80.5% on day 1 and 3, respectively, due to the high density of AMP on the surface. In addition, the cytotoxicity of the uniform surface would further increase with its antimicrobial activity (Supplementary Figs. 23 and 24). It could also demonstrate the limitation of the mono-functionalized surfaces for biomaterials, suggesting the need to develop a AMP/RGD dual-peptide functionalized gradient surface.

### The dual-functionalized gradient Ti surface

The RGD-functionalized surfaces exhibited negligible antimicrobial activity and improved biocompatibility, whereas the AMP-functionalized surface had evident cytotoxicity but excellent antimicrobial activity at an improper peptide density. In addition, there exists an optimized density of RGD or AMP for best biocompatibility or antimicrobial capability. Therefore, we proceeded to construct RGD/AMP dual-peptide functionalized gradient surface and used such surface as a high-throughput platform for determining the best combination of RGD and AMP density that can achieve excellent combination of biocompatibility and antimicrobial activity.

**Fig. 6:**
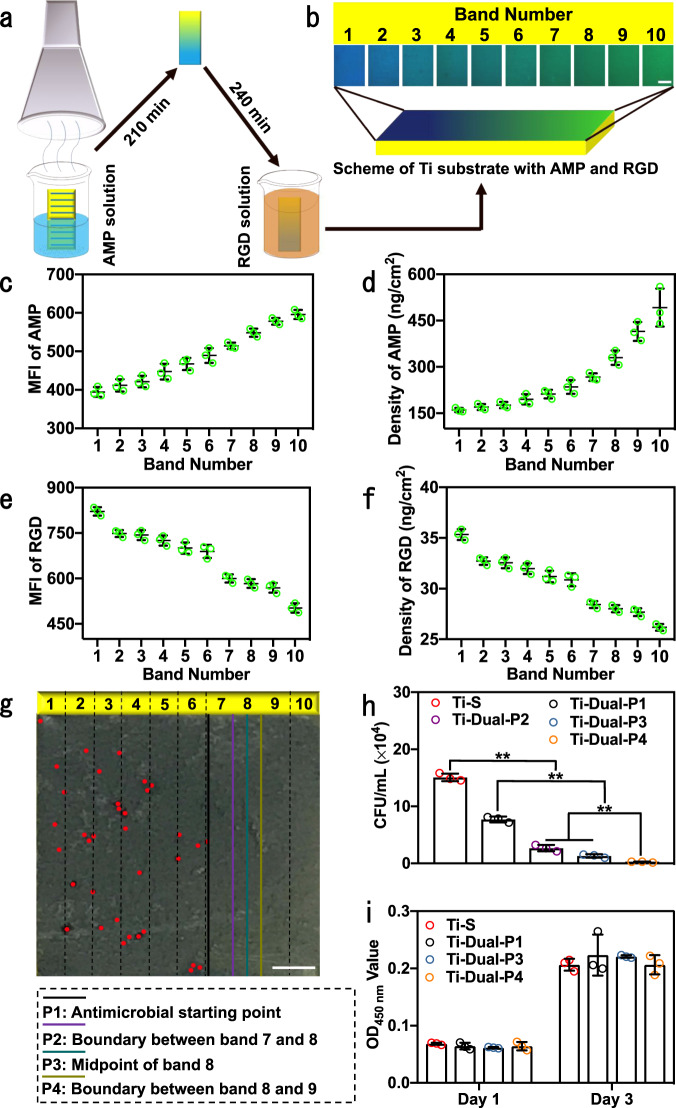
The characterization of the dual-functionalized gradient/uniform Ti surfaces. **a** Schematic diagram depicting the preparation of the dual-functionalized gradient Ti surface (Ti-Grad-Dual), in which Ti-Grad-50AMP was first prepared and then incubated into RGD solution (0.5 μM) for 240 min. The gradient in the AMP density on Ti-Grad-50AMP would cause a gradient in the density of unconjugated MAL on this substrate. Thus, incubation of Ti-Grad-50AMP in Mca-labeled RGD would give rise to a gradient in the RGD density. A higher AMP density band with a lower density of unconjugated MAL would be corresponding to a lower RGD density. The surface was divided into ten bands with a band width of 1 mm. Band 1 had the lowest density of AMP but highest density of RGD, and band 10 exhibited the opposite trend. **b** Fluorescence images of AMP–FITC and RGD–Mca on Ti-Grad-Dual, which were obtained by fluorescence microscope under the FITC and DAPI channel (*n* = 3, scale bar, 200 μm). **c** The MFI of Ti-Grad-50AMP with AMP–FITC and **d** the densities of AMP calculated by the fluorescence method. **e** MFI of Ti-Grad-Dual with RGD–FITC and **f** the densities of RGD calculated by the fluorescence method. In each band, we randomly selected three points to calculate the MFI in **c** and **e** (*n* = 3). **g** Distribution of *S. aureus* on Ti-Grad-Dual detected by the Petrifilm method (*n* = 3, scale bar, 1.5 mm). Four lines are denoted, including P1, P2, P3, and P4, corresponding to the boundary between the area without and with dead bacteria, boundary between bands 7 and 8, and midpoint position within band 8, and boundary between bands 8 and 9, respectively. **h** Antimicrobial assay of the indicated uniform Ti surfaces against *S. aureus* by an agar plate method (*n* = 3) (Sidak’s multiple comparisons test, two-way ANOVA. Ti–S vs Ti-Dual-P1, **p = 0.0001, Ti–S vs Ti-Dual-P2, **p < 0.0001; Ti–S vs Ti-Dual-P3, **p < 0.0001; Ti–S vs Ti-Dual-P4, **p < 0.0001; Ti-Dual-P1 vs Ti-Dual-P2, **p = 0.0003; Ti-Dual-P1 vs Ti-Dual-P3, **p < 0.0001; Ti-Dual-P1 vs Ti-Dual-P4, **p < 0.0001; Ti-Dual-P2 vs Ti-Dual-P4, **p = 0.0018; Ti-Dual-P3 vs Ti-Dual-P4, **p = 0.0036). **i** CCK-8 results for the indicated uniform Ti surfaces with *mBMSCs* after 1 and 3 days of culturing (*n* = 3). Nongradient Ti surfaces generated from the conditions (immersion time and concentration) corresponding to P1, P2, P3, and P4 in **g**, were termed Ti-Dual-P1, Ti-Dual-P2, Ti-Dual-P3, and Ti-Dual-P4, respectively, in **h** and **i**. Data are displayed as mean ± SD and analyzed by GraphPad Prism software.

To achieve this goal, we prepared the complementary dual-functionalized gradient Ti surface (abbreviated as Ti-Grad-Dual) by immersing Ti-Grad-50AMP in RGD solution (0.5 μM) for 240 min (Fig. 6a). Ti-Grad-50AMP had been proved to show a gradient in the AMP density, which would cause a gradient in the density of unconjugated MAL on this surface (Supplementary Fig. 25). Hence, when Ti-Grad-50AMP was incubated in thiolated Mca-labeled RGD, the RGD would be conjugated onto the unconjugated MAL sites on the surface by the thiol–ene click reaction, leading to a gradient in the RGD density on the Ti-Grad-50AMP. On the resultant Ti-Grad-Dual surface, a higher AMP density band would have a lower RGD density. We then aimed to identify a position with a specific AMP and RGD density that could lead to a combination of biocompatibility and antimicrobial capability.

Indeed, the fluorescence image of the dual-functionalized surface prepared by AMP–FITC and RGD–Mca demonstrated that the gradient direction of AMP and RGD were opposite; the band with a higher AMP density had a lower RGD density due to the competition for the reaction sites on the same surface (Fig. 6b and magnified in Supplementary Fig. 26). Hereafter, we used FITC to label both AMP and RGD so that we could use fluorescence from FITC, instead of using both FITC and Mca, to calculate the densities of AMP and RGD, to eliminate the interaction between different fluorescence dyes and achieve more accurate quantification^68–70^. As Ti-Grad-Dual was prepared from Ti-Grad-50AMP, these two surfaces had similar distributions of AMP. By the fluorescence method and the standard curve (Supplementary Fig. 16), the MFI of Ti-Grad-50AMP increased from 394.5 ± 12.6 a.u. in band 1 to 595.7 ± 11.8 a.u. in band 10 (Fig. 6c), corresponding to an increase in the AMP density from 160.0 ± 6.9 ng/cm^2^ (one molecule in 2.1 nm^2^) to 492.1 ± 61.5 ng/cm^2^ (one molecule in 0.7 nm^2^) on Ti-Grad-50AMP (as well as on Ti-Grad-Dual) (Fig. 6d and Supplementary Fig. 27). We further employed the combination of AMP and RGD–FITC to calculate the RGD density on Ti-Grad-Dual, and found that the RGD density was decreasing from band 1 and 10, opposite to the trend of AMP density. The RGD density was indeed determined to decrease from 35.3 ± 0.5 ng/cm^2^ (one molecule in 4.2 nm^2^) in band 1 to 26.2 ± 0.3 ng/cm^2^ (one molecule in 5.6 nm^2^) in band 10 on Ti-Grad-Dual (Fig. 6e, f and Supplementary Figs. 7 and 28). These results verified the complementary distribution of AMP and RGD, and indicated successful preparation of the dual-functionalized gradient surface.

The gradient antimicrobial activity of Ti-Grad-Dual was characterized by the Petrifilm method (Fig. 6g). When the band number was increasing from 1 to 10, the surface started to completely kill bacteria starting from the position marked as the black line and denoted as P1 in band 7 (corresponding to reaction with AMP of 128.0 μM for 127.9 min and with RGD of 0.5 μM for 240.0 min). The other three positions with excellent antimicrobial activities, marked as P2, P3, and P4 in Fig. 6g, were the boundary between bands 7 and 8 (corresponding to reaction with AMP of 166.7 μM for 147.0 min and with RGD of 0.5 μM for 240.0 min), midpoint of band 8 (reaction with AMP of 200.0 μM for 157.5 min and RGD of 0.5 μM for 240.0 min), and boundary between bands 8 and 9 (reaction with AMP of 250.0 μM for 168.0 min and RGD of 0.5 μM for 240.0 min). Unlike Ti-Grad-Dual (Fig. 6g) and Ti-Grad-20AMP (Fig. 5a) with gradient antimicrobial activities, Ti-Grad-50AMP had an excellent antimicrobial activity on the whole surface without gradient change (Supplementary Fig. 29a) due to the high density of AMP. For example, the density of AMP in band 1 of Ti-Grad-50AMP was 160.0 ng/cm^2^, which was similar to that in band 6 of Ti-Grad-20AMP (171.2 ng/cm^2^) with an antimicrobial activity (Figs. 4d, 5a, and 6d). In addition, Ti-Grad-20Dual (prepared by immersing Ti-Grad-20AMP in 0.5 μM of RGD for 240.0 min) did not display any antimicrobial activity (Supplementary Fig. 29b), probably because RGD could eliminate the antimicrobial activity of AMP on the surface by the cancellation of the positive charge in AMP via aspartic acid^71^. However, the AMP could only display antimicrobial activities when its density was higher than 266.7 ng/cm^2^ (the density in band 7 on Ti-Grad-Dual), which was much higher than that in band 10 of Ti-Grad-20AMP (223.9 ng/cm^2^) (Figs. 4d and 6d). This result also demonstrated the significance of finding a condition for dual-peptide engineered surface with a combination of excellent antimicrobial activity and biocompatibility.

We prepared the nongradient dual-functionalized surfaces of Ti-Dual-P1, Ti-Dual-P2, Ti-Dual-P3, and Ti-Dual-P4 using parameters corresponding to P1–P4 on Ti-Grad-Dual. We calculated the densities of AMP and RGD on these surfaces based on the standard curve (Supplementary Figs. 7 and 16) and fluorescence images (Supplementary Fig. 30). The densities of AMP on Ti-Dual-P1, Ti-Dual-P2, Ti-Dual-P3, and Ti-Dual-P4 were 238.1 ± 16.0, 274.7 ± 21.9, 305.4 ± 31.2, and 338.2 ± 26.2 ng/cm^2^, respectively (Supplementary Table 2), which were consistent with the densities between bands 7 and 8 on Ti-Grad-Dual (266.7 ± 12.3–329.7 ± 23.4 ng/cm^2^) (Fig. 6d). The densities of RGD on Ti-Dual-P1, Ti-Dual-P2, Ti-Dual-P3, and Ti-Dual-P4 were 31.2 ± 1.2, 29.9 ± 0.6, 28.4 ± 0.8, and 27.8 ± 0.4 ng/cm^2^, respectively (Supplementary Table 2), which were also similar to the densities between bands 7 and 8 on Ti-Grad-Dual (28.4 ± 0.3–28.0 ± 0.3 ng/cm^2^) (Fig. 6f). Moreover, compared to Ti–S, these surfaces exhibited antimicrobial activities and exhibited 50.2, 81.7, 93.3, and 98.2% inhibition of *S. aureus*, respectively (Fig. 6h). Besides excellent antimicrobial activity, Ti-Dual-P4 also exhibited good biocompatibility, being similar to Ti–S (Fig. 6i). These results indicate that RGD improved the biocompatibility of the AMP-functionalized antimicrobial surfaces. And the best molar ratio of AMP to RGD was 5.3:1 for a combination of excellent antimicrobial activity and biocompatibility.

### In vivo antimicrobial and osteogenesis assay in the infection model

**Fig. 7:**
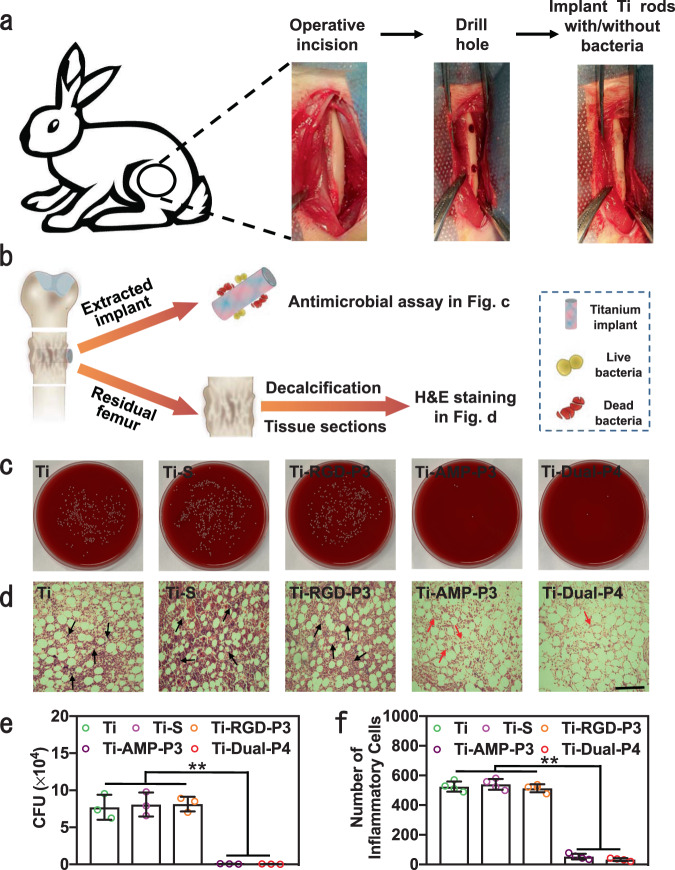
In vivo assay of antimicrobial activity of the implants modified by peptides. **a** Schematic diagram and processes of the surgery. **b** Schematic of antimicrobial assay and H&E staining to evaluate the antimicrobial ability of the implants. For the antimicrobial assay, the implants were extracted from femur, cultured and characterized on blood agar plates. For H&E staining, the residual tissues of femur without implants were made into tissue section for pathological examination. **c** The images of the blood agar plates in the indicated groups (*n* = 3). **d** The H&E staining images of the bone tissues after implant were taken out. The inflammatory cells and cytoplasm were pointed out by the black and red arrows, respectively (*n* = 4, scale bar, 200 μm). **e** Antimicrobial activities of the implants against *S. aureus* by the agar plate method. Three implants were collected for each group (*n* = 3). (Sidak’s multiple comparisons test, two-way ANOVA. Ti vs Ti-AMP-P3, **p = 0.0014; Ti vs Ti-Dual-P4, **p = 0.0014; Ti–S vs Ti-AMP-P3, **p = 0.0010; Ti–S vs Ti-Dual-P4, **p = 0.0010; Ti-RGD-P3 vs Ti-AMP-P3, **p = 0.0001; Ti-RGD-P3 vs Ti-Dual-P4, **p = 0.0001.) **f** The number of inflammatory cells determined from the H&E staining images. Four images were collected for each group (*n* = 4). (Sidak’s multiple comparisons test, two-way ANOVA. Ti vs Ti-AMP-P3, **p < 0.0001; Ti vs Ti-Dual-P4, **p < 0.0001; Ti–S vs Ti-AMP-P3, **p < 0.0001; Ti–S vs Ti-Dual-P4, **p < 0.0001; Ti-RGD-P3 vs Ti-AMP-P3, **p < 0.0001; Ti-RGD-P3 vs Ti-Dual-P4, **p < 0.0001.) Data are displayed as mean ± SD and analyzed by GraphPad Prism software.

Furthermore, we employed the extracted parameters of P3 on Ti-Grad-RGD, P3 on Ti-Grad-20AMP, and P4 on Ti-Grad-Dual to prepare the functionalized Ti rods (also denoted as Ti-RGD-P3, Ti-AMP-P3, and Ti-Dual-P4) and characterized the antimicrobial activity of the implants in vivo in the New Zealand rabbit bone defect model (Fig. 7a). After 7 days of acute infection, the antimicrobial assay and hematoxylin and eosin (H&E) staining were used to evaluate the antimicrobial activity in vivo (Fig. 7b). Both Ti-AMP-P3 and Ti-Dual-P4 rods exhibited an excellent antimicrobial activity compared to Ti rods, killing 99.2 and 99.6% of *S. aureus* on the implant, respectively, while Ti-RGD-P3 rods had no obvious antimicrobial activity (Fig. 7c, e). In addition, the immunohistochemistry staining results showed that bacteria (the spots pointed by red arrows) were present in the tissues around Ti, Ti–S, and Ti-RGD-P3 rods, while almost no bacteria were observed in the tissues around Ti-AMP-P3 and Ti-Dual-P4 rods (Supplementary Fig. 31). H&E staining results showed that the tissues around Ti–S and Ti-RGD-P3 rods had clear infiltration of inflammatory cells (blue spots pointed by black arrows), while the tissues around Ti-AMP-P3 and Ti-Dual-P4 rods had little destruction and infection (Fig. 7d). The inflammatory cell contents in Ti-AMP-P3 and Ti-Dual-P4 groups were 90.2 and 93.9% lower than those in the Ti group (Fig. 7f). It was reported that the serious infiltration of inflammatory cells was mainly caused by bacterial infection rather than by the antimicrobial implants^72–74^. Overall, Ti-AMP-P3 and Ti-Dual-P4 rods have the best antimicrobial activity.

**Fig. 8:**
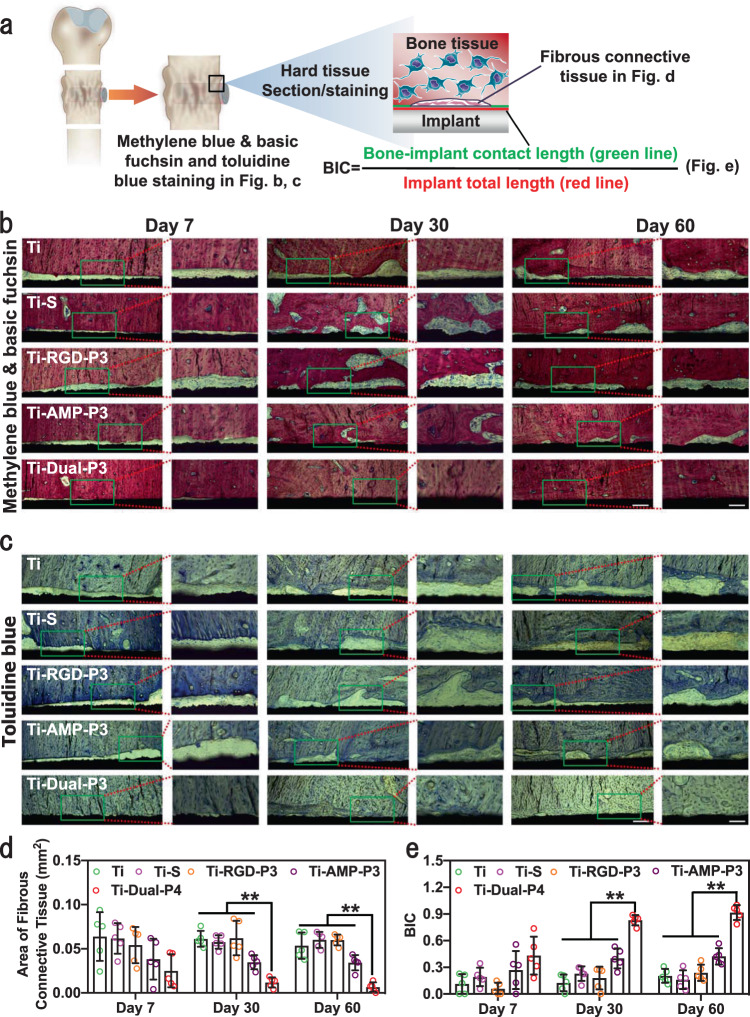
In vivo osteogenesis assay in the infection model. **a** Schematic for preparing hard tissue sections and assessing the osseointegration of implants in vivo. After the hard tissue sections were prepared from the femur with implants, they were stained with methylene blue and basic fuchsin and toluidine blue. The area of fibrous connective tissue at the interface between bone tissue and implant was quantitatively analyzed using Image J software. The bone–implant contact length and implant total length were measured by software in the microscope to calculate the bone–implant contact (BIC). **b** The methylene blue and basic fuchsin (*n* = 5) and **c** toluidine blue staining (*n* = 3) images of the hard tissue section of implantation. The interface between the implant and tissue in the green rectangle was enlarged (the scale bars before and after enlargement (×10 and ×20 magnification) were 200 and 100 μm, respectively). Quantitative analysis of methylene blue and basic fuchsin staining: **d** area of fibrous connective tissue and **e** BIC at the interface between bone tissue and implant. Five sections in each group were chosen for the analysis (*n* = 5). (Sidak’s multiple comparisons test, two-way ANOVA. In d-day 30: Ti vs Ti-Dual-P4, **p < 0.0001; Ti–S vs Ti-Dual-P4, **p < 0.0001; Ti-RGD-P3 vs Ti-Dual-P4, **p = 0.0006; Ti-AMP-P3 vs Ti-Dual-P4, **p = 0.0008. In d-day 60: Ti vs Ti-Dual-P4, **p = 0.0002; Ti–S vs Ti-Dual-P4, **p < 0.0001; Ti-RGD-P3 vs Ti-Dual-P4, **p < 0.0001; Ti-AMP-P3 vs Ti-Dual-P4, **p = 0.0003. In e-day 30: Ti vs Ti-Dual-P4, **p < 0.0001; Ti–S vs Ti-Dual-P4, **p < 0.0001; Ti-RGD-P3 vs Ti-Dual-P4, **p < 0.0001; Ti-AMP-P3 vs Ti-Dual-P4, **p < 0.0001. In e-day 60: Ti vs Ti-Dual-P4, **p < 0.0001; Ti–S vs Ti-Dual-P4, **p < 0.0001; Ti-RGD-P3 vs Ti-Dual-P4, **p < 0.0001; Ti-AMP-P3 vs Ti-Dual-P4, **p < 0.0001.) Data are displayed as mean ± SD and analyzed by GraphPad Prism software.

We further characterized the osseointegration of the implants by the in vivo infection model adopted in other reports^75,76^ (Fig. 8a). After 7, 30, and 60 days of implantation, the results of methylene blue and basic fuchsin and toluidine blue staining of the hard tissue sections (Fig. 8b, c) showed that compared to other groups, there were negligible fibrous connective tissues around Ti-Dual-P4. Especially after 30 and 60 days of implantation, tight osteointegration was formed between Ti-Dual-P4 and tissues. Meanwhile, the amount of fibrous connective tissues around Ti-AMP-P3 was lower than that around Ti, Ti–S, and Ti-RGD-P3, as Ti-AMP-P3 could inhibit infection to protect the osteointegration. We quantitatively analyzed the methylene blue and basic fuchsin staining results as in other reports^77–79^. The results (Fig. 8d, e) showed that after 60 days of implantation and compared to Ti, Ti–S, and Ti-RGD-P3, the area of fibrous connective tissue at the interface of Ti-Dual-P4 was significantly reduced by 87.8%, 89.2% and 89.1%, respectively. The BIC of Ti-Dual-P4 was 4.5-, 5.7-, and 3.8-folds that of Ti, Ti–S, and Ti-RGD-P3, respectively. Although compared to Ti, Ti–S, and Ti-RGD-P3, the area of fibrous connective tissue at the interface of Ti-AMP-P3 was lower and its BIC was higher, Ti-AMP-P3 was not as good as Ti-Dual-P4, demonstrating that the single functionalization of AMPs with antimicrobial activities was not enough for the formation of the connective tissue.

In addition, following a reported method^76,80,81^, we employed the in vivo noninfection model to characterize the osteointegration of the implants (Fig. 7a). The results also showed that Ti-Dual-P4 had excellent osteointegration without bacterial infection, similar to that of Ti-RGD-P3 (Supplementary Fig. 32).

Collectively, the in vivo results demonstrated that only the dual-functionalized Ti implant (Ti-Dual-P4) exhibited the best combination of both excellent antimicrobial activity and osteogenic activity for bone defect repair at the same time.

### Extension of the high-throughput strategy to Au substrates

We further demonstrated that our strategy displayed broad-spectrum applications for other biomaterials, i.e., Au, which had wide application in biosensing^82,83^. We integrated the above peptides on Au directly via the thiol group. Similar to Ti, we prepared the RGD-functionalized, AMP-functionalized, and dual-peptide-functionalized gradient surfaces, and prepared the uniform surfaces with the optimized parameters extracted from these gradient surfaces (Supplementary Figs. 33–59). The results demonstrated that our strategy can be extended to the controlled dual-peptide modification on a variety of biomaterials.

## Discussion

Biofunctionalization of surfaces with peptides is considered a suitable strategy for the design of biomaterials^10,84^. And for this strategy, previous studies showed that the density of peptides on the surfaces often played a critical role in achieving desired biofunctions^48^. Nonetheless, until now, there is still a lack of an efficient strategy for rational design and preparation of the biofunctionalized surfaces. For the functionalized surface, particularly the dual-functionalized surface on which a peptide may affect the function of another one, complex orthogonal tests were always employed to optimize a large number of variables, i.e., the densities and ratios of peptides, reaction time, and reactant concentrations^11,85^.

The gradient surface had been used to reveal the relationships between the cells and the surface properties^45–50,55,85,86^. In contrast to these reports, we demonstrated that the gradient surface could serve as a high-throughput platform to screen the optimized preparation parameters for rational design and preparation of the biofunctionalized Ti surface. In our strategy, due to the controllable preparation of gradient surface, the above-mentioned rational reaction parameters, i.e., reaction time and reactant concentration, could be extracted accurately. When a substrate was treated with these parameters, a nongradient surface was prepared with the densities and ratios of peptides highly similar to those of the specific site on the gradient surface. Subsequently, the uniform surfaces exhibited desired biofunctions, for both the mono- and dual-functionalized implant. These results demonstrated the high accuracy of our high-throughput strategy. Meanwhile, the extension of this high-throughput strategy to Au substrates illustrated that it had a broad-spectrum application.

We employed the orthopedic Ti implants as the substrates to verify the feasibility of our high-throughput strategy. Biomaterial-associated infection (BAI) and the delay of osseointegration are two main clinical reasons for the failure of surgery^87–89^. Previous reports and clinical experience showed that overcoming one of these two problems was not enough^90^. Our study also confirmed this phenomenon. For example, a mono-functionalized surface, Ti-RGD-P3, had high risk of bacterial infection due to the lack of AMP, but another one, Ti-AMP-P3, had fibrous connective tissues around it and showed delayed osseointegration due to the lack of RGD. Unfortunately, in the previous study, it is very difficult to explore Ti implants with both excellent antimicrobial activity and the promotion of osseointegration simultaneously by simply mixing two peptides because these two peptides had contradictory biological effects^91^. And finding their best density/ratio turned out to be difficult. However, with our high-throughput strategy to maximize the functions of the two peptides in one biomaterial, we could rationally design the dual-functionalized implant, i.e., Ti-Dual-P4, to show these two contradictory biological activities can be achieved at the same time. Compared with the implants functionalized with a single peptide, the multifunctional implant Ti-Dual-P4 could inhibit BAI and promote osseointegration in both infection and noninfection models in vivo (Figs. 7 and 8 and Supplementary Fig. 32). Also, the reported dual-functionalized implants often required the use of external stimuli, including electricity^91^, light^75^, and temperature^92^, but our rationally designed dual-functionalized implant did not rely on the external stimuli, making our approach more practical in the clinic.

Herein, a high-throughput strategy was developed based on gradient surfaces for rational design and preparation of peptide functionalized surfaces. This strategy provided a series of optimized parameters in a convenient and rapid manner, including the densities and ratios of the peptides, and the related preparation parameters of reaction time and reactant concentrations. With the extracted parameters from the gradient surface, we prepared both mono- and dual-peptide functionalized Ti surfaces with highly similar biofunctions to those of the specific site on the gradient surface, displaying biocompatibility or/and antimicrobial activity both in vitro and in vivo. In addition, our strategy could be extended to functionalize other biomaterials. We believe that this high-throughput method holds great potential for rational design and preparation of biomaterial surface.

## Methods

### Materials

Ti substrates (square with side length of 4 or 10 mm, Chemhui Metal Materials Ltd., Baoji, China), Gold (Au) substrates (square with side length of 4 or 10 mm, Tsinghua-Foxconn Nanotechnology Research Center, Beijing, China), silane-PEG2000-MAL (Ponsure Biotech, Inc., Shanghai, China), peptides (GL Biochemical Ltd., Shanghai, China), cells and bacteria (*mBMSCs*, ATCC CRL-12424; *S. aureus*, ATCC 6538P. VWR International, LLC, Pennsylvania, USA), cell counting kit-8 (CCK-8, Dojindo, Shanghai, China), and Petrifilm (Minnesota Mining and Manufacturing Company, Saint Paul, USA) were purchased. The reagents for the cell assay were got from Gibco (USA). The reagents for bacterial cultivation were purchased from Guangdong Huankai Microbial Technology Co., Ltd. (Guangzhou, China). The other reagents for preparation of the surfaces were got from Tianjin Damao Factory (Tianjin, China).

### Pretreatment of the Ti substrates

Ti substrates were cleaned ultrasonically with ethanol/deionized water, dried by nitrogen stream, and treated by 5 M NaOH (60 °C for 24 h). After being cleaned with deionized water for three times and dried by nitrogen stream, Ti substrates were reacted with 1 mL of silane-PEG2000-MAL solution (0.5 mg/mL, 95% ethanol and 5% deionized water) at 50 °C in an oil bath for 12 h. Then, Ti substrates were washed with ethanol, dried by nitrogen stream, and denoted as Ti–S.

### Preparation of the RGD-functionalized gradient Ti surface

The RGD-functionalized gradient Ti surface was prepared based on the “perfusion” technique. Ti–S (10 mm × 10 mm) was placed vertically into a 24-well plate. RGD or RGD–FITC was dissolved in ethanol at the specified concentrations (0.1, 0.5, or 1 μM). Then, the solution was injected into a well (0.5 mL/h) with a single-syringe pump (LSP01-1A, LONGER, Baoding). After 4 h, the whole substrate would be immersed in the solution. The sample was then cleaned with ethanol for three times immediately from top to bottom and denoted as Ti-Grad-*m*RGD, in which *m* represents the concentration of RGD solution (*m* = 0.1, 0.5, or 1 μM).

### Preparation of the AMP-functionalized gradient Ti surface

The AMP-functionalized gradient Ti surface was prepared based on the “evaporation” technique. Briefly, Ti–S (10 mm × 10 mm) was placed vertically in a 24-well plate, and AMP or AMP–FITC was dissolved in ethanol at the concentrations of 1, 20, or 50 μM. Then, 2 mL of the solution was injected to immerse the sample completely. The system was placed under air ejector fans. The air speed was controlled to evaporate the solution completely in 210 min. After that, the surface was washed with ethanol for three times immediately from top to bottom and denoted as Ti-Grad-mAMP, in which *m* represents the initial concentration of AMP solution (*m* = 1, 20, or 50 μM).

### Preparation of the dual-functionalized gradient Ti surface

The dual-functionalized gradient Ti surface was prepared based on the AMP-functionalized gradient Ti surface. Briefly, Ti-Grad-50AMP or Ti-Grad-20AMP was immersed into 0.5 μM of RGD, RGD–Mca, or RGD–FITC solution for 4 h. Then, the surfaces were cleaned with ethanol immediately from top to bottom, denoted as Ti-Grad-Dual (from Ti-Grad-50AMP) or Ti-Grad-20Dual (from Ti-Grad-20AMP).

### Calculation of the peptide density

The peptide density was calculated by the reported fluorescence method^11^. Briefly, a standard calibration curve of MFI-density was obtained as follows. 0.6 μL of AMP–FITC or RGD–FITC with different concentrations in deionized water was added directly onto Ti–S or Au surfaces (4 mm × 4 mm). The peptide solution concentration ranged from 4.43 μM (calculated with a peptide density of 0.1 molecule per nm^2^, which corresponded to the grafting densities of AMP–FITC and RGD–FITC of 42.2 and 23.2 ng/cm^2^, respectively) to 132.90 μM (determined from a peptide density of three molecules per nm^2^, which corresponded to the grafting densities of AMP–FITC and RGD–FITC of 1266.0 and 696.0 ng/cm^2^, respectively). After that, MFI of the surface was obtained via the fluorescence microscope (Eclipse Ti-U, Nikon) by choosing ten points randomly.

By the obtained standard curves, the densities of the peptides on the surfaces were calculated according to the MFI of the band/surface (10 mm × 10 mm) with RGD–FITC or AMP–FITC by adding 3.75 μL of deionized water. Three points in one band or five points on one uniform surface were chosen randomly for the calculation. Particularly, for the dual-functionalized gradient/uniform surface, we hypothesized that the integration of RGD would not impact the immobilized AMP. Therefore, the MFI values of the surfaces with AMP–FITC were collected as described above before the integration of RGD. In addition, we employed the combination of AMP and RGD–FITC to calculate the density of RGD on the dual-functionalized surface, and the MFI values of the surfaces were collected as described above.

### Extraction of the parameters from the specific sites on the gradient surface for producing uniform surfaces

The preparation parameters of the specific site on the gradient surface were extracted, including the peptide densities, reaction times, and reactant concentrations. For the RGD-functionalized gradient surface, the reaction time of the specific site (*t*_*x*_) was the same as that of the band having this site, and could be calculated as follows:

1 $${{t}}_{{x}}={{t}}_{{p}}\times \frac{{{N}}_{{x}}}{{N}}$$

where *t*_*p*_ was the total time of the titering, *N*_*x*_ was the band no. for a specific site, and *N* was the number of the total bands (*n* = 10).

For the AMP-functionalized gradient surface and dual-functionalized gradient surface, the reaction time of the specific site with AMP (*t*_*x*_) and the reactant concentration of AMP (*C*_*x*_) at time *t*_*x*_ were calculated as follows:

2 $${{t}}_{{x}}={{t}}_{{e}}\times \frac{{{l}}_{{x}}}{{l}}$$

3 $$\,{{C}}_{{x}}=C\times \frac{{l}}{{{l-l}}_{{x}}}$$

where *t*_*e*_ was the total time of the evaporation, *l*_*x*_ was the vertical distance between the specific site and the bottom of the substrate, *l* was the length of the substrate sides, and *C* was the initial concentration of the AMP.

Based on these parameters, we prepared the uniform surfaces by immersing the samples (Ti–S or Au) into the solution with the given concentration of peptide for the specific reaction time. Then, the surfaces were washed with ethanol for three times immediately.

### Antimicrobial assay

The antimicrobial activity of the samples against *S. aureus* was characterized by the Petrifilm or the agar plate. For Petrifilm assay, the samples (10 mm × 10 mm) were incubated with the bacterial suspension (20 μL, 1 × 10^5^ CFU/mL in PBS) in 24-well plate. Meanwhile, the Petrifilm was pretreated with 1 mL of sterilized water. After 2 h of culturing (37 °C), the samples were dried naturally in a biological safety cabinet, and transferred to the Petrifilm immediately. After another 24 h of culturing (37 °C), the bacterial distribution was observed on the Petrifilm directly. In agar plate assay, the samples were incubated with the bacterial suspension (20 μL, 1 × 10^5^ CFU/mL in PBS) in 24-well plate. After 2 h of culturing (37 °C), the samples were moved into a tube with the bacterial suspension. Then, 1.98 mL of PBS was injected into the tube. The tube was ultrasonicated for 2 min, and vortexed for another 1 min. After that, the detached bacteria were transferred to a new tube, and 60 μL of the suspension was added on the agar plate to characterize the bacterial viability.

### Cell assay

Mice *mBMSCs* were cultured with medium (High-Dulbecco’s modified Eagle Medium containing 10% fetal bovine serum). Cells in passages 3–5 were used for the following assays. The Ti samples (10 mm × 10 mm) were placed in 24-well plate, to which *mBMSCs* (3 × 10^4^ cells per well) were added. In the fluorescence-based assay, after 24 h, the Ti samples were cleaned with PBS, and treated with 4 vol% neutral paraformaldehyde solution at 4 °C overnight. Then, the Ti samples were treated with 0.1% Triton for 12 min, F-actin solution (10 mg/L) for 60 min, and DAPI solution (5 mg/L) for 6 min. After that, the samples were cleaned with PBS and characterized with the Eclipse Ti-U instrument (Nikon, Japan) under the FITC and DAPI channels. To characterize the distribution of the cells on the gradient surface, we obtained 9 images along the gradient direction of the sample, then combined them into one image, and divided the combined image into 10 bands.

The biocompatibilities of the Ti samples were characterized by the CCK-8 assay. After being cultured with the cells for 24 and 72 h, the Ti samples were transferred to new 24-well plates. Then, the complete medium (350 μL) and the CCK-8 solution (35 μL) were added for each Ti sample. After 3 h of culturing, the OD value of the solution (100 μL) was characterized at the wavelength of 450 nm.

### In vivo assay

All in vivo experiments were approved by the Institutional Animal Care and Use Committee of Guangdong Medical Laboratory Animal Center (Foshan, China). Overall, 40 rabbits (12-week old and ~1.8 kg) were divided into five groups for in vivo assay (*n* = 8). First, all the rabbits were anesthetized with pentobarbital sodium, and two holes perpendicular to the centerline of the femur were drilled with an electric drill (Φ 2 mm) in each rabbit femur (the distance between the two holes was 1 cm). Then, for the infection model, 15 μL of bacterial solution (containing 7.5 × 10^6^ CFU of *S. aureus*) was injected into the implant site as described in the literature^93^, and Ti rods (Φ 2 mm × 6 mm) were implanted. For the noninfection model, the Ti rods were implanted without bacteria. After being sealed with bone candle, the skin was stitched with sutures. Then, the rabbits were raised with the food without antibiotics.

For the antimicrobial assay, on the 7th day, the implants were extracted from the infection model of euthanized rabbits, treated with 3 mL of nutrient broth for 3 h at 37 °C, and vortexed for 1 min. Subsequently, the suspension was collected and diluted 10^3^-fold and 10^4^-fold in PBS. The diluted suspension (10 μL) was employed to characterize the bacterial viability on blood agar plates. The residual femur was successively treated with 4% formaldehyde for 3 days and EDTA solution for 60 days. The decalcified tissue was successively dehydrated with 75, 85, 90, 95, and 100% ethanol for 4, 2, 2, 1, and 0.5 h, respectively. After being treated with dimethylbenzene (three times, each for 10 min), the samples were fixed with paraffin wax (60 °C, 1 h), and cooled for solidification. After that, the tissue sections (4 μm) were obtained and baked (40 °C, 36 h). For the immunohistochemistry staining, the tissues were boiled in sodium citrate buffer for antigen retrieval and then incubated with primary antibody (anti-protein A, Abcam, Cambridge, UK) and secondary antibody (IgG H&L (HRP), Cambridge, UK) successively. Each antibody was diluted for 1000 times. For the pathological examinations, the tissue slices were treated with dimethylbenzene (two times, each for 15 min) and ethanol (three times, each for 5 min). The samples were washed with deionized water (5 min) and treated with hematoxylin (2 min). Then, the slices were treated by eosin solution (5 min), dehydrated with alcohol (10 min) as well as dimethylbenzene (30 min). Finally, the samples were characterized with the Eclipse Ti-U instrument (Nikon, Japan) under the bright field, and the inflammatory cell numbers were calculated from four areas.

For the osseointegration assay, after implantation for indicated times in infection or noninfection model, the Ti rods were collected and fixed in 10% formalin, dehydrated with acetone in −20 °C for eight times, and subsequently embedded in poly(methylmethacrylate). Then, the sections (10–20 μm) were made with a diamond histological saw, stained with methylene blue and basic fuchsin or toluidine blue for histological observation by the Eclipse Ti-U instrument (Nikon, Japan) under the bright field channels. The methylene blue and basic fuchsin staining images with ×10 magnification were employed for quantitative analysis, in which nearly a whole side of the interface between the implant and the cortical bone could be shown (~2 mm). Specifically, the area of fibrous connective tissue at the interface between bone tissue and implant was measured by Image J software, while the area in the bone tissue (not in contact with implant) was not calculated. The bone–implant contact length and implant total length were measured by the software in Eclipse Ti-U instrument (Nikon, Japan) to calculate the bone–implant contact (BIC, defined as bone–implant contact length/implant total length). Five sections in each group were chosen for the quantitative analysis.

### Statistics and reproducibility

The data shown as the mean ± standard deviation (SD) were obtained by at least three independent experiments and analyzed by the GraphPad Prism 8.0.0 software. The statistical significance of observed differences was analyzed by ANOVA test.

### Reporting summary

Further information on research design is available in the Nature Research Reporting Summary linked to this article.

## Supplementary information

Supplementary Information

Reporting Summary

## Data Availability

All data are reported in this manuscript and supporting information also available from the authors upon request.
