# Supplementary material for: High-throughput screening and rational design of biofunctionalized surfaces with optimized biocompatibility and antimicrobial activity
Source: Nat Commun. 2021 Jun 18;12:3757. doi: 10.1038/s41467-021-23954-8 (PMC8213795; doi:10.1038/s41467-021-23954-8)
Supplement: Supplementary file 1 — Supplementary Information [file 41467_2021_23954_MOESM1_ESM.pdf]

High-throughput Screening and Rational Design of Biofunctionalized Surfaces with Optimized Biocompatibility and Antimicrobial Activity

*Zhou Fang[1,2,&], Junjian Chen[2,3,&], Ye Zhu[4], Guansong Hu[2,3], Haoqian Xin[1,2], Kunzhong Guo[1,3], Qingtao Li[1], Liangxu Xie[5], Lin Wang[1, 6, &, *], Xuetao Shi[3,*], Yingjun Wang[2,*], Chuanbin Mao[4,,7*]*

Supplementary Information

1. National Engineering Research Center for Tissue Restoration and Reconstruction, South China University of Technology, Higher Education Mega Center, Panyu, Guangzhou 510006, China. E-mail: wanglin3@scut.edu.cn

2. School of Materials Science & Engineering, South China University of Technology, Higher Education Mega Center, Panyu, Guangzhou 510006, China. Email: imwangyj@scut.edu.cn

3. School of Biomedical Science and Engineering, South China University of Technology, Higher Education Mega Center, Panyu, Guangzhou 510006, China. Email: shxt@scut.edu.cn

4. Department of Chemistry and Biochemistry, University of Oklahoma, Stephenson Life Sciences Research Center Norman, OK, 73019, USA. E-mail: maophage@gmail.com

5. Institute of Bioinformatics and Medical Engineering, Jiangsu University of Technology, Changzhou 213001, China.

6. Bioland Laboratory (Guangzhou Regenerative Medicine and Health Guangdong Laboratory), Guangzhou 510005, China.

7. School of Materials Science & Engineering, Zhejiang University, Hangzhou 310027, China.

* wanglin3@scut.edu.cn

* shxt@scut.edu.cn

* imwangyj@scut.edu.cn

* maophage@gmail.com

[&] Z.F, J.C and L.W contributed equally

Methods

Pretreatment of the Au substrates. Au substrates were washed ultrasonically by acetone, ethanol and deionized water. Then, the substrates were treated with piranha solution (V(H_2SO_4) : V(30% H_2O_2) = 7:3) at 60 °C in a water bath for 15 min, and washed again in an ultrasonic bath successively with ethanol/water. After that, the substrates were dried by nitrogen stream and denoted as Au.

Preparation of the RGD-functionalized gradient Au surfaces. The preparation of the RGD-functionalized gradient Au surfaces was similar to that of titanium (Ti), which was based on the "titration" technique. Briefly, the cleaned Au (10 mm × 10 mm) was placed vertically in a 24-well plate. RGD or RGD-FITC solution (0.1 μM, 1 μM or 10 μM in ethanol) was injected into the well (3 mL/h). After 40 min, the substrate would be immersed in the solution completely. Then, the substrate was cleaned with ethanol for three times immediately from top to bottom and denoted as Au-Grad-mRGD, in which m represents the concentration of RGD solution (m = 0.1, 1 or 10).

Preparation of the AMP-functionalized gradient Au surfaces. Similar to that of the Ti, the preparation of the AMP-functionalized gradient Au surfaces was based on the "evaporation" technique. Briefly, the samples (10 mm × 10 mm) were fixed vertically in 24-well plate, and AMP or AMP-FITC (2 mL with the concentration of 5 μM or 50 μM in ethanol) was added into the well. Then, by controlling the evaporation rate, the solution was evaporated completely in 150 min. The surface was washed with ethanol for three times immediately from top to bottom and denoted as Au-Grad-mAMP, in which m represents the initial concentration of AMP solution (m = 5 or 50).

Preparation of the dual-functionalized gradient Au surfaces. The dual-functionalized gradient Au surfaces was prepared by immersing Au-Grad-50AMP into 0.1 μM of RGD, RGD-FITC or RGD-Mca in ethanol for 40 min. Then, the surfaces were cleaned with ethanol immediately from top to bottom, denoted as Au-Grad-Dual.

Quartz crystal microbalance (QCM-D) assay. Au electrode was cleaned and tested with QCM-D (Q-Sense AB, Sweden). Ethanol was introduced into Au electrode until the frequency was balanced. Then, 50 μM RGD solution in ethanol was introduced onto the electrode at a rate of 29.14 μL/min. After balancing, the electrodes were rinsed with ethanol to attain balance again.

X-ray photoelectron spectroscopy (XPS) assay. The XPS assay was performed on the photoelectron spectrometer with the X-ray source of Al Kα (1486.4 eV) (AXIS ULTR DLD, Kratos, England). The high-resolution spectra were generated with the pass energy of 40 eV. For the gradient surface, each band was characterized at the middle area with the step length of 1 mm.

Atomic force microscope (AFM) assay. The AFM images were acquired by a MultiMode Nanoscope IIIa atomic force microscope (Digital Instruments Inc., Santa Barbara, CA). The tapping mode was used with the pixel of 384 × 384 and 7 kHz scanning rate at a displacement resolution of 10 μm. The Ra value of each band on the surface was counted. Each band was characterized with the step length of 1 mm for 3 times.

Fourier transform infrared spectrometer (FTIR) assay. The FTIR spectra were collected on Vertex 70 (Bruker, Germany) under the absorption mode. The spectra

were recorded within a spectral region of 600-4000 cm^{-1} with a resolution of 4 cm^{-1} and 32 scans per point in band 1, 6 and 10.

All-atom molecule dynamics (MD) simulation. The parameters and topology files of the FITC groups were created with *antechamber* and *tleap* modules of Amber16[1]. The partial charges of the FITC groups were calculated using the AM1-BCC method. The missing parameters were extracted from the GAFF force field[2]. The structures of AMP, AMP-FITC, RGD and RGD-FITC peptides were initially constructed using *tleap* and *antechamber* modules of Amber16 based on the amino acid sequences[1]. The parameters for normal residues were described by the AMBER99SB-ILDN force field. The four peptides were solvated with a TIP3P water model. All topology files were converted to GROMACS format using the ACPYPE 0.0.1 tool[3]. The solvent molecules were randomly and partly substituted by chloride and sodium ions to neutralize the system charge. The simulations were carried out in a canonical ensemble with the temperature kept at 300 K by a v-rescale thermostat. A Maxwell-Boltzmann distribution under 300 K allocated the initial velocity of each atom. The van der Waals interactions were calculated by a switched potential, and the short-range electrostatic interactions were calculated by a cutoff distance of 12 Å and the long-range interactions were computed by a particle mesh Ewald method. All bonds were constrained by the LINCS algorithm. The energies of all the models were firstly minimized to eliminate the steric clashes and inappropriate geometry. Next, a 500 ps NVT equilibration with position restraints on heavy atoms was conducted to equilibrate the solvent and ions. Then, a 500 ps NPT equilibration was conducted. Finally, MD simulation of 100 ns was performed in GROMACS 5.1.4 software package, with the first 50 ns for equilibration and the last 50 ns for analysis. The visualization was conducted by PyOML[4] 2.4.0.

To compute the solvent accessible surface area (SASA) of the FITC group in the AMP-FITC and RGD-FITC, we adopted the double cubic lattice method (DCLM) for computing the average solvent accessible surface area in the last 50 ns of the MD simulation with the "gmx_sasa" program in GROMACS 5.1.4.

The RGD-functionalized Au surfaces. In the present study, we demonstrated that our high-throughput technique could also be applied on Au surface by integrating the above peptides directly via the thiol group[5]. We first prepared the RGD-functionalized gradient Au surfaces (Au-Grad-0.1RGD, Au-Grad-1RGD and Au-Grad-10RGD) by the "titration" technique. According to other report[6] and the QCM-D results (Supplementary Fig. 33), we found that the reaction between Au and thiol group could be finished around 30 min. To ensure the completed reaction, we set the perfusion time as 40 min in the present study. The mean fluorescence intensity (MFI) of Au-Grad-0.1RGD with RGD-FITC increased from 167.0 ± 9.8 a.u. in band 1 to 264.4 ± 12.4 a.u. in band 10 (Supplementary Fig. 34a, b). According to the standard curve (Supplementary Fig. 35), we found that there was a linear relation (in reverse proportion) between MFI and the reciprocal inverse of the density of RGD-FITC. By calculation, the densities of RGD increased from 21.8 ± 0.3 ng/cm^2 (1 molecule in 6.7 nm^2) in band 1 to 26.0 ± 0.6 ng/cm^2 (1 molecule in 5.6 nm^2) in band 10 (Supplementary Fig. 34c). The XPS N1s high-resolution spectra also showed a gradient trend with increasing N1s intensities from band 1 to band 10 (Supplementary Fig. 36).

With the gradient distribution of RGD, Au-Grad-0.1RGD exhibited gradient biocompatibility, and the cell density and cell spreading area were highest (Supplementary Fig. 37) in band 9 and band 10 while the densities of RGD

were 25.2 ± 0.5 ng/cm^2 and 26.0 ± 0.6 ng/cm^2, respectively (Supplementary Fig. 34c). Based on the extracted parameters in band 3, band 9 and band 10 on Au-Grad-0.1RGD, we prepared uniform surfaces of Au-RGD-P1 (0.1 μM of RGD for 10 min), Au-RGD-P2 (0.1 μM of RGD for 36 min) and Au-RGD-P3 (0.1 μM of RGD for 40 min). The MFI of Au-RGD-P1, Au-RGD-P2, and Au-RGD-P3 were 182.3 ± 10.2 a.u., 243.5 ± 19.3 a.u. and 252.2 ± 29.4 a.u., respectively (Supplementary Fig. 38a, b, c). By the fluorescence method, the corresponding densities of RGD were 22.4 ± 0.4 ng/cm^2, 25.0 ± 0.7 ng/cm^2 and 25.5 ± 1.0 ng/cm^2, respectively, which were similar to those in band 3 (23.0 ± 0.3 ng/cm^2), band 9 (25.2 ± 0.5 ng/cm^2) and band 10 (26.0 ± 0.6 ng/cm^2) on Au-Grad-0.1RGD. Further CCK-8 assay showed that Au-RGD-P2 and Au-RGD-P3 had higher biocompatibilities, which were 1.2-fold and 1.2-fold of the value for pristine Au (Supplementary Fig. 39).

We also prepared the uniform surfaces with higher density of RGD, *i.e.*, Au-RGD-P4 (1 μM of RGD solution for 40 min) and Au-RGD-P5 (10 μM of RGD solution for 40 min). Both surfaces had high MFI values of 1423.4 ± 36.7 a.u. and 3590.1 ± 53.8 a.u., respectively (Supplementary Fig. 38d, e), demonstrating higher densities of RGD. Interestingly, negligible improvement of biocompatibility was observed for Au-RGD-P4 and Au-RGD-P5 compared to Au (1.0-fold and 1.0-fold, respectively) (Supplementary Fig. 39). Additionally, although there were gradient distributions of RGD on Au-Grad-1RGD and Au-Grad-10RGD (Supplementary Fig. 40, 41), we did not find the gradient distribution of cell density and cell spreading area on these two surfaces (Supplementary Fig. 42, 43). These results were consistent with those of the Ti surface (Fig. 3b and Supplementary Fig. 10-14) and other

reports[7], suggesting that the biocompatibility of the surface would not be improved with the excessive density of RGD.

The above results provided us the efficient parameters to prepare the biocompatible Au surface, *i.e.*, the optimized densities of RGD on the Au surface were assumed to be between 25.2 ng/cm^2 and 26.0 ng/cm^2, which could be obtained by incubating Au into 0.1 μM of RGD solution for 36 to 40 min. We also revealed that these optimized densities on the Au surface were less than those on the Ti surface, which might be caused by the high biocompatibility of Au compared to Ti[8].

The AMP-functionalized Au surfaces. The AMP-functionalized gradient Au surfaces could be prepared by the "evaporation" technique. As mentioned in the *Main Text*, we extended the evaporation time to 150 min to ensure the completed reaction and to decelerate the increasing speed of the concentration of AMP to decrease the deviation of extracted reactant concentration from the actual value. With an initial concentration of 50 μM, the peptide exhibited a gradient distribution on Au-Grad-50AMP, and the MFI ranged from 196.7 ± 10.1 a.u. to 330.6 ± 6.4 a.u. (Supplementary Fig. 44a, b). Based on the standard curve (Supplementary Fig. 45), we also found that there was a linear relation between MFI and the reciprocal density of AMP-FITC. By calculation, the densities of the peptide ranged from 132.5 ± 7.0 ng/cm^2 (1 molecule in 2.5 nm^2) in band 1 to 435.5 ± 49.5 ng/cm^2 (1 molecule in 0.8 nm^2) in band 10 (Supplementary Fig. 44c), which was consistent with the XPS N1s high-resolution spectra (Supplementary Fig. 46). Further antimicrobial assay showed that Au-Grad-50AMP exhibited gradient antimicrobial activity from the point with the black line, which we denoted as P1 (antimicrobial starting

point; 69.5 μM of AMP for 42.1 min) (Supplementary Fig. 47). Moreover, we denoted the purple line as P2 (midpoint of band 4; 76.9 μM of AMP for 52.5 min) and the cyan line as P3 (terminus of band 4, 83.3 μM of AMP for 60 min). According to the results of the distribution of AMP on Au-Grad-50AMP (Supplementary Fig. 44c), the densities of AMP from P1 to P3 ranged from approximately 173.8 ± 24.4 ng/cm^2 to 192.4 ± 25.4 ng/cm^2. Additionally, we found that there was no gradient distribution of AMP on Au-Grad-5AMP (Supplementary Fig. 48), which should be caused by the insufficient initial concentration of AMP. Consequently, the surfaces of Au and Au-Grad-5AMP did not exhibit antimicrobial activity due to the lack of AMP, and the bacteria distributed uniformly on the surfaces (Supplementary Fig. 49).

Based on the extracted parameters from Au-Grad-50AMP, we prepared the uniform surfaces of Au-AMP-P1, Au-AMP-P2 and Au-AMP-P3. As determined by the fluorescence method, the densities of AMP on these three surfaces were 165.4 ± 9.7 ng/cm^2, 186.4 ± 12.8 ng/cm^2 and 205.8 ± 13.4 ng/cm^2, respectively, (Supplementary Fig. 50), which were similar to those of the specific sites on the gradient surface. Compared to Au, Au-AMP-P1, Au-AMP-P2 and Au-AMP-P3 could exhibit 59.7%, 84.5% and 99.5% inhibition of *S. aureus*, respectively (Supplementary Fig. 51).

These results demonstrated that our gradient surface could provide parameters to prepare the antimicrobial Au surface. Namely, the efficient densities of AMP on the Au surface were assumed to be around 205.8 ± 13.4 ng/cm^2, which could be obtained by incubating Au with 83.3 μM of AMP for 60 min. Additionally, we found that the antimicrobial activity was sensitive to the density of AMP, which increased from 84.5% to 99.5% with the addition of

only approximately 20 ng of AMP per cm^2 (from 186.4 ± 12.8 ng/cm^2 on Au-AMP-P2 to 205.8 ± 13.4 ng/cm^2 on Au-AMP-P3). Unfortunately, similar to AMP-functionalized Ti surfaces, Au-AMP-P1, Au-AMP-P2 and Au-AMP-P3 exhibited cytotoxicity, as demonstrated by 1.1%, 5.7% and 18.7% inhibition of *mBMSCs* after 3 days of culturing (Supplementary Fig. 52).

The Dual-functionalized Au surfaces. Due to the cytotoxicity of the antimicrobial surface, we further designed dual-functionalized Au surfaces by incubating Au-Grad-50AMP in 0.1 μM of RGD solution for 40 min. The fluorescence images of the surfaces prepared by AMP-FITC and RGD-Mca demonstrated that the distributions of the two peptides were opposite (Supplementary Fig. 53). As Au-Grad-Dual was prepared from Au-Grad-50AMP, the dual-functionalized surface should have similar distribution of AMP, which was from 196.7 ± 10.1 a.u. in band 1 to 330.6 ± 6.4 a.u. in band 10 (Supplementary Fig. 44a, b). We employed the combination of AMP/RGD-FITC to calculate the distribution of RGD on Au-Grad-Dual. The results showed that the distribution of RGD followed the opposite trend as the band number, and the MFI decreased from 222.4 ± 20.2 a.u. in band 1 to 147.2 ± 12.3 a.u. in band 10 on Au-Grad-Dual (Supplementary Fig. 54a, b). According to the standard curve (Supplementary Fig. 35), the MFI results demonstrated a decrease in the densities of RGD from 24.0 ± 0.7 ng/cm^2 (1 molecule in 6.1 nm^2) to 21.2 ± 0.4 ng/cm^2 (1 molecule in 6.9 nm^2) (Supplementary Fig. 54c).

Because of the distribution of peptides, Au-Grad-Dual began to exhibit antimicrobial activity from the point with the black line in band 4, denoted as P1 (antimicrobial starting point; 73.2 μM of AMP for 47.5 min; 0.1 μM of

RGD for 40.0 min) (Supplementary Fig. 55). Compared to Au-Grad-50AMP, the antimicrobial starting point on Au-Grad-Dual moved to the band with larger number, demonstrating the similar phenomenon in Ti that RGD could eliminate the antimicrobial activity of AMP on the surface by the cancellation of the positive charge in AMP via aspartic acid[8]. We also denoted the purple line as P2 (midpoint of band 4; 76.9 μM of AMP for 52.5 min; 0.1 μM of RGD for 40.0 min) and the cyan line as P3 (terminus of band 4; 83.3 μM of AMP for 60 min; 0.1 μM of RGD for 40.0 min). Additionally, we found that similar to Au-Grad-5AMP, the dual-functionalized gradient surface Au-Grad-5Dual (prepared from Au-Grad-5AMP) exhibited negligible antimicrobial activity, and the bacteria distributed uniformly (Supplementary Fig. 56).

Considering the antimicrobial activity, we prepared the uniform surfaces of Au-Dual-P3 according to the extracted parameters from the sites of P3 on Au-Grad-Dual. By the fluorescence method, we calculated the densities of the AMP on Ti-Dual-P3 with AMP-FITC, which was 196.7 ± 12.7 ng/cm^2 (Supplementary Fig. 57a). With the combination of AMP/RGD-FITC, the densities of RGD on Ti-Dual-P3 was 22.6 ± 1.0 ng/cm^2 (Supplementary Fig. 57b). These densities were similar to those on the specific sites of Au-Grad-Dual, and Au-Dual-P3 exhibited antimicrobial activity, inhibiting 94.7% of *S. aureus*, respectively (Supplementary Fig. 58). Furthermore, unlike the AMP-functionalized surface, Ti-Dual-P3 exhibited improved biocompatibilities (Supplementary Fig. 59). Particularly, after 3 days of culturing, Au-Dual-P3 exhibited better biocompatibility than Au (1.1-fold). It demonstrated that we could obtain the optimized parameters for the dual-functionalized Au surface by our gradient surface, on which the densities

of AMP and RGD were 196.7 ± 12.7 ng/cm^2 and 22.6 ± 1.0 ng/cm^2, respectively, with the molecular ratio of 3.8:1. Moreover, this surface could be prepared by immersing the surface in 83.3 μM of HHC36 for 60 min, and consequently in 0.1 μM of RGD for 40.0 min.

The above results demonstrated that our high-throughput technique based on the gradient surface showed a broad application on Au surface. By this technique, we could obtain the optimized parameters, *i.e.*, the densities and ratios of the peptides, and the related preparation parameters, to prepare the Au surfaces with excellent biocompatibility and/or antimicrobial activity.

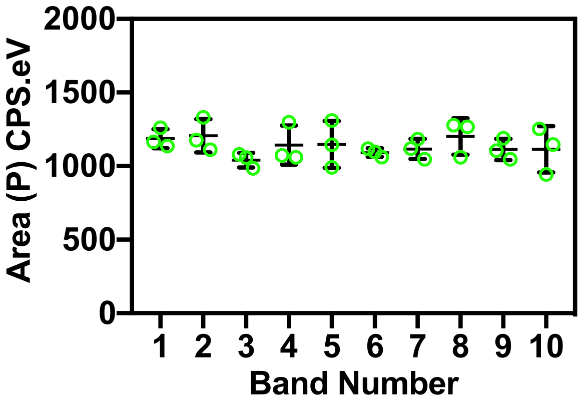

Supplementary Fig. 1 | Peak area of Si2p high-resolution spectra of each band on Ti-S (n=3). Data are displayed as mean ± SD. See Experimental section of "X-ray photoelectron spectroscopy (XPS) assay" in the Supporting Information for details of the assay.

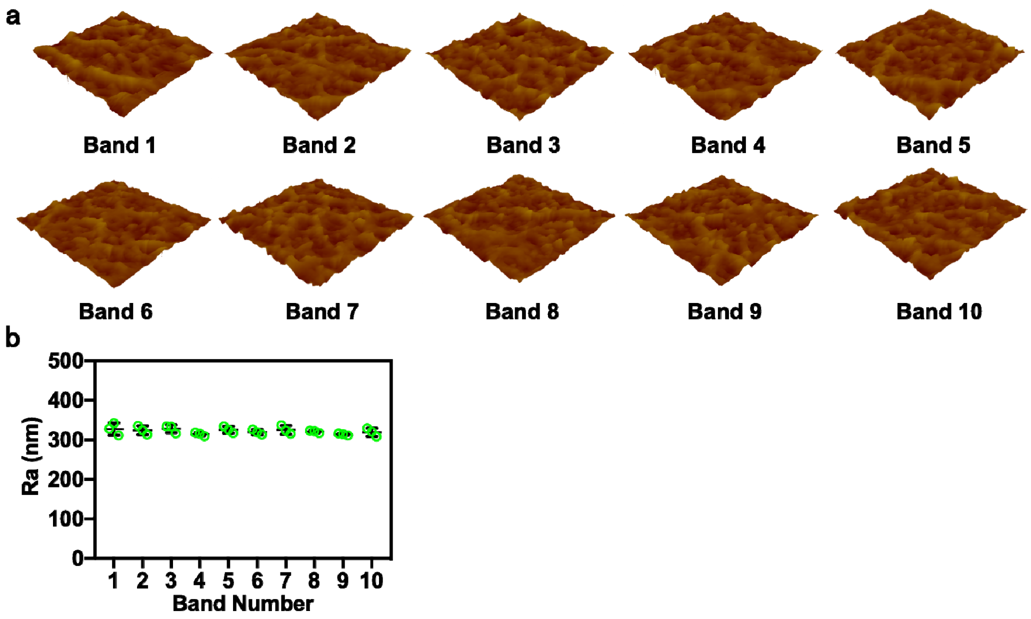

Supplementary Fig. 2 | AFM images and roughness of bands on Ti-S. (a) The AFM images of all bands on Ti-S. (b) The Ra values of each band on Ti-S calculated from the AFM images (n=3). Data are displayed as mean ± SD. See Experimental section of "Atomic force microscope (AFM) assay" in the Supporting Information for details of the assay.

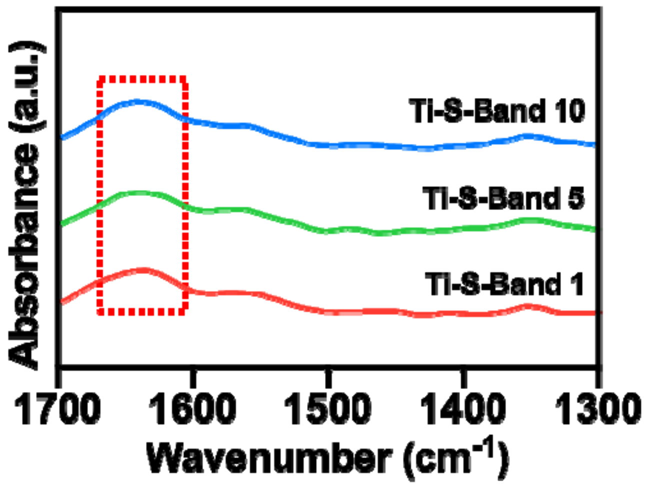

Supplementary Fig. 3 | FTIR spectra of the amide bond in the specific band on Ti-S. See Experimental section of "Fourier transform infrared spectrometer (FTIR) assay" in the Supporting Information for details of the assay.

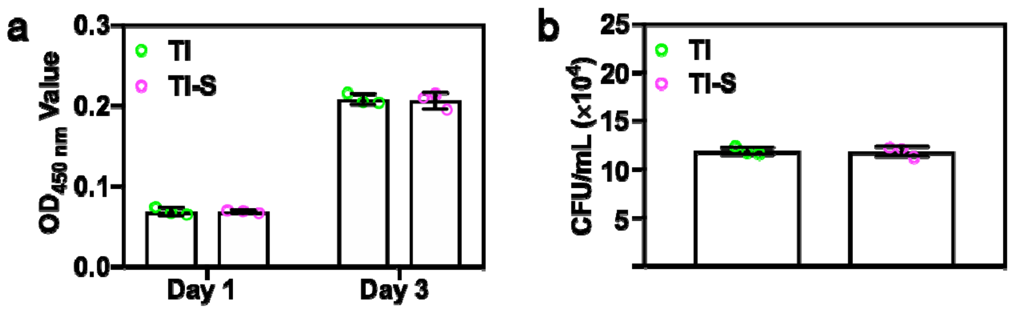

Supplementary Fig. 4 | CCK-8 and antimicrobial results of pristine Ti and Ti-S. (a) CCK-8 results of the indicated uniform Ti surfaces with *mBMSCs* after 1 and 3 days of culturing. The *mBMSCs* were seeded at the concentration of 3×10^4 cells on each sample (n=3). (b) Antimicrobial assay of the indicated uniform Ti surfaces against *S. aureus* by an agar plate method after 2 h of culturing. The bacterial concentration was 10^5 CFU/mL (n=3). Data are displayed as mean ± SD. See Experimental section of "Cell assay" and "Antimicrobial assay" in the Main Text for details of the assay.

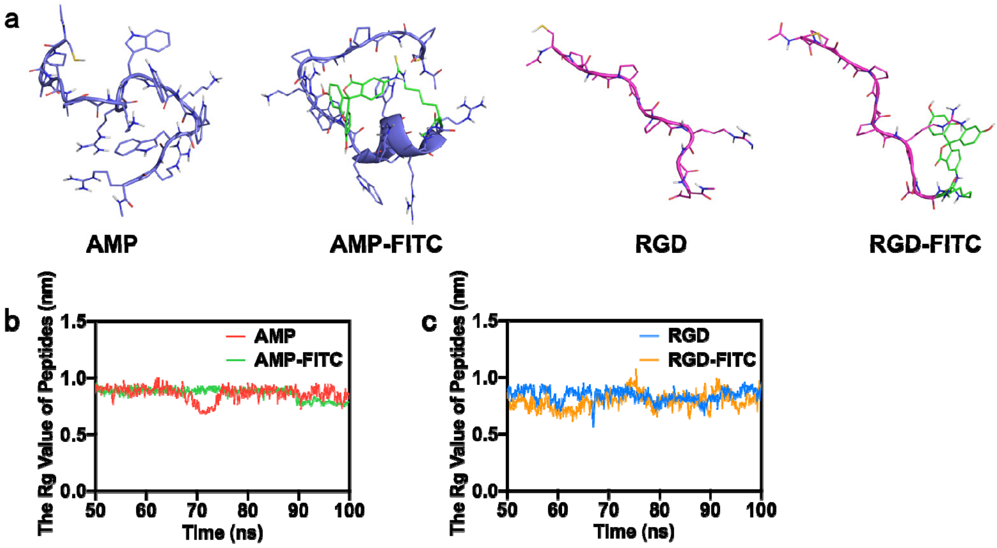

Supplementary Fig. 5 | All-atom molecular dynamic (MD) simulation of the peptides. (a) The dominant configuration of the peptides. (b) The radius of gyration (Rg) of AMP and AMP-FITC during 50-100 ns. (c) The radius of gyration (Rg) of RGD and RGD-FITC during 50-100 ns. See Experimental section of "All atom molecular dynamics (MD) simulation" in the Supporting Information for details of the assay.

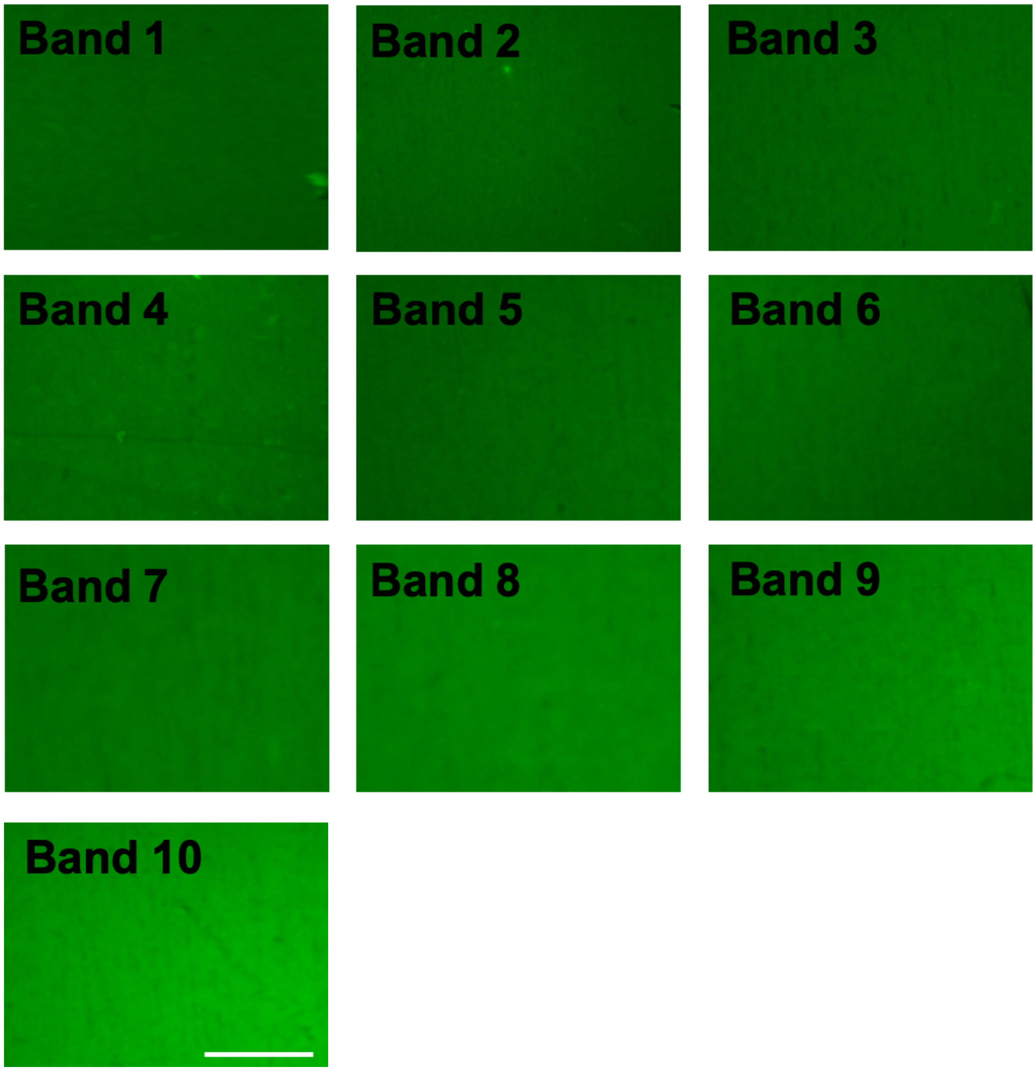

Supplementary Fig. 6 | The magnified FITC fluorescence images of different bands on Ti-Grad-0.5RGD (n=3, scale bar, 200 μm). See Experimental section of "Preparation of the RGD-functionalized gradient Ti surfaces" in the Main Text for details of the assay.

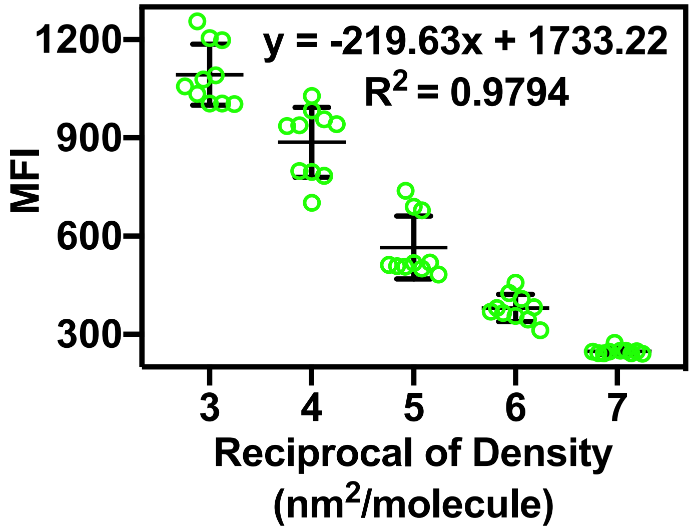

Supplementary Fig. 7 | The standard curve of MFI and the reciprocal inverse of the density of RGD-FITC on Ti-S. The curve was obtained by fluorescence microscope under the FITC channel. On each surface, we randomly selected 10 points to calculate the MFI (n=10). There was an inverse linear relation between MFI and the reciprocal of the RGD-FITC density. Data are displayed as mean ± SD. See Experimental section of “Calculation of the density of peptide” in the Main Text for details of the assay.

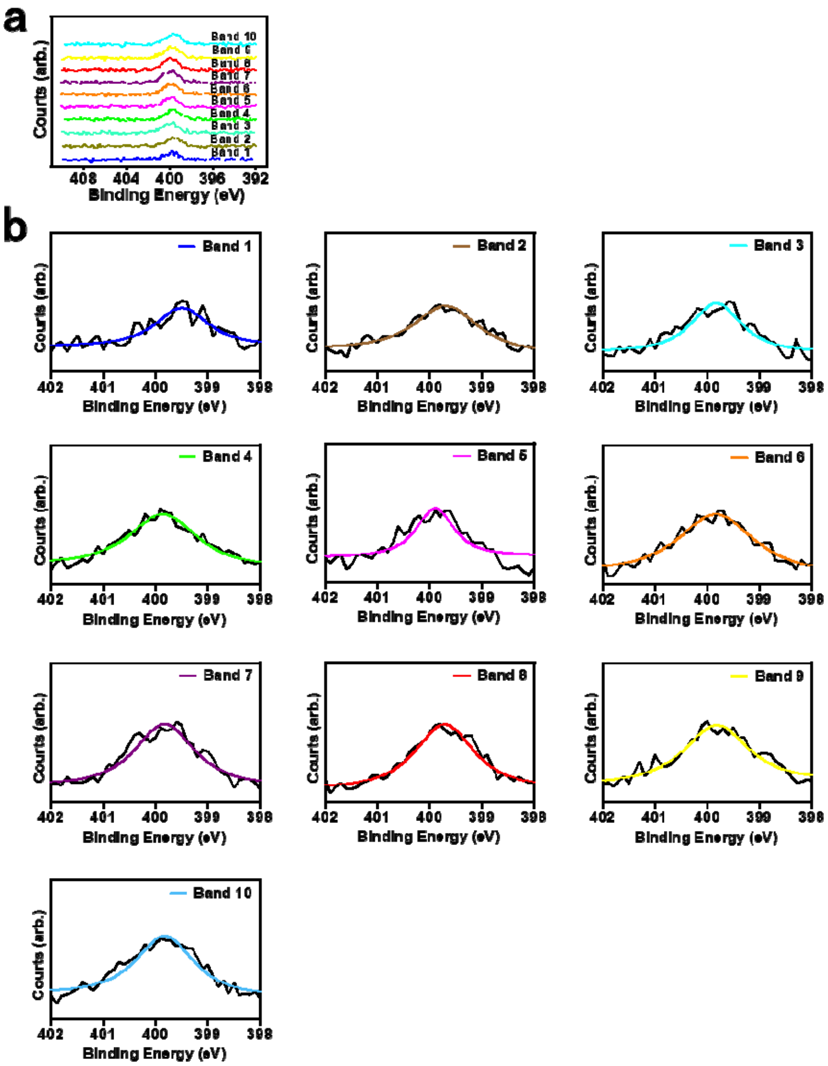

Supplementary Fig. 8 | (a) XPS N1s high-resolution spectra of the indicated bands on Ti-Grad-0.5RGD. (b) The enlarged XPS N1s high-resolution spectrum of each band in (a) with the binding energy from 398 eV to 402 eV was shown. See Experimental section of "X-ray photoelectron spectroscopy (XPS) assay" in the Supporting Information for details of the assay.

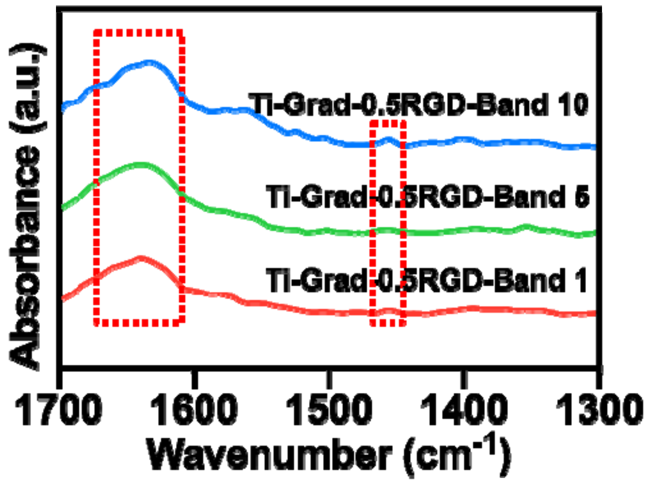

Supplementary Fig. 9 | FTIR spectra of the amide bond in the specific bands on Ti-Grad-0.5RGD. See Experimental section of "Fourier transform infrared spectrometer (FTIR) assay" in the Supporting Information for details of the assay.

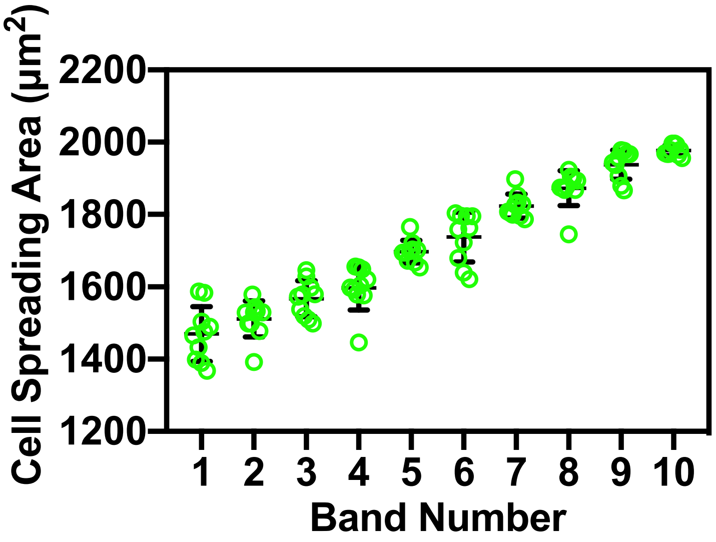

Supplementary Fig. 10 | The cell spreading area of *mBMSCs* in the indicated bands on Ti-Grad-0.5RGD. The cells were stained with F-actin and DAPI after 24 h of culturing, and subsequently being observed. In each band, we randomly selected 10 cells to calculate the cell spreading area (n=10). Data are displayed as mean ± SD. See Experimental section of "Cell assay" in the Main Text for details of the assay.

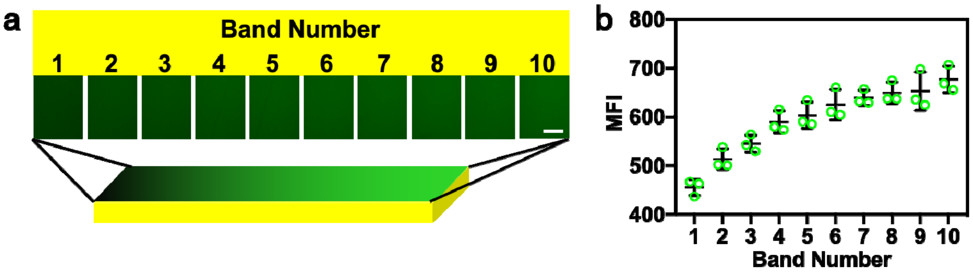

Supplementary Fig. 11 | The gradient properties of Ti-Grad-0.1RGD with gradient RGD density along the vertical direction. (a) The FITC fluorescence images of Ti-Grad-0.1RGD. The images of the 10 bands were collected individually and lined up as they were originally on the Ti substrate (n=3, scale bar, 200 μm). (b) The mean fluorescence intensity (MFI) of each band of Ti-Grad-0.1RGD. In each band, we randomly selected 3 points to calculate the MFI (n=3). Data are displayed as mean ± SD. See Experimental section of "Preparation of the RGD-functionalized gradient Ti surfaces" in the Main Text for details of the assay.

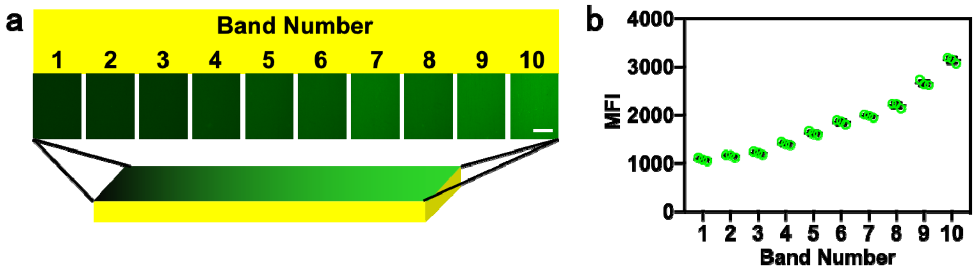

Supplementary Fig. 12 | The gradient properties of Ti-Grad-1RGD with gradient RGD density along the vertical direction. (a) The FITC fluorescence images of Ti-Grad-1RGD. The images of the 10 bands were collected individually and lined up as they were originally on the Ti substrate (n=3, scale bar, 200 μm). (b) The mean fluorescence intensity (MFI) of each band of Ti-Grad-1RGD. In each band, we randomly selected 3 points to calculate the MFI (n=3). Data are displayed as mean ± SD. See Experimental section of "Preparation of the RGD-functionalized gradient Ti surfaces" in the Main Text for details of the assay.

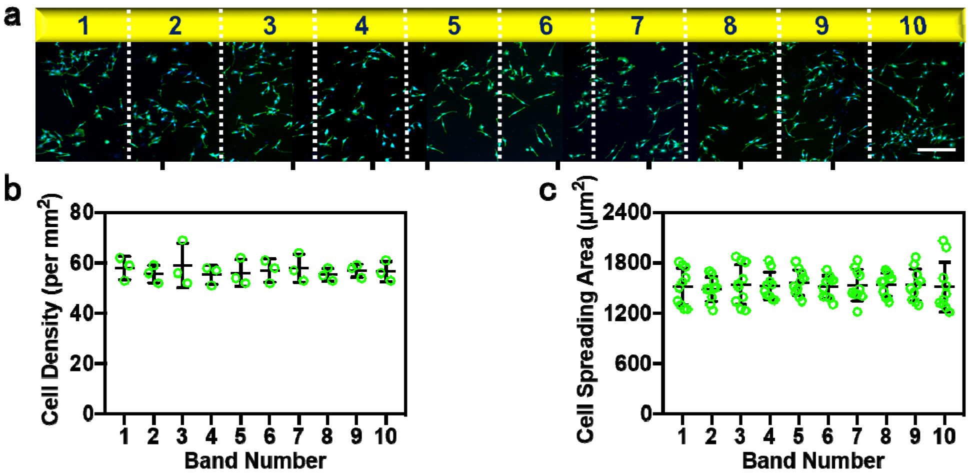

Supplementary Fig. 13 | The distribution of *mBMSCs* on Ti-Grad-0.1RGD. (a) Distribution (n=3), (b) average number (n=3 within a band) and (c) cell spreading area (n=10 within a band) of fluorescently stained *mBMSCs* on different bands of Ti-Grad-0.1RGD after 24 h of culturing. After being stained with F-actin and DAPI, the cells were observed by fluorescence microscope under the FITC and DAPI channels (scale bar, 400 μm). Data are displayed as mean ± SD. It should be noted that 9 images were obtained along the gradient direction of one sample and combined to form the image in **a**, and the dotted lines in **a** highlight the band boundaries (but not the dividing lines of the 9 images). The actual dividing boundaries of the 9 images are marked as solid black lines at the bottom of the image in **a**. See Experimental section of “Cell assay” in the Main Text for details of the assay.

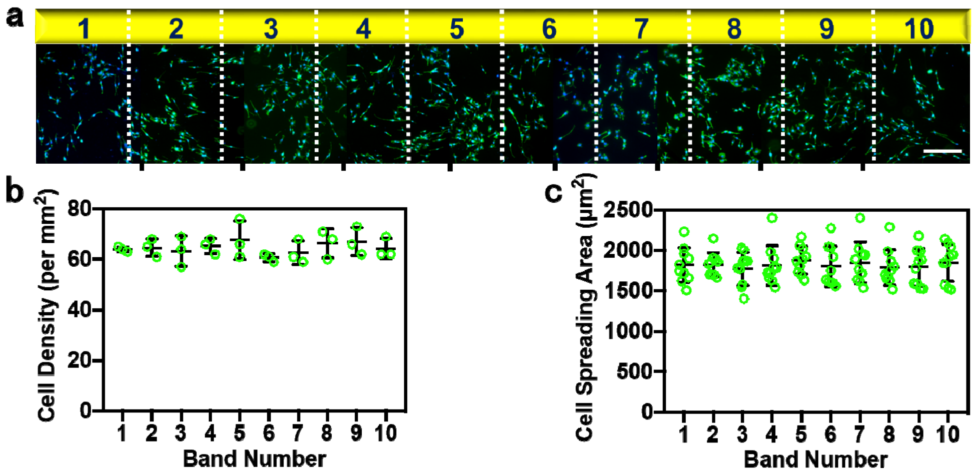

Supplementary Fig. 14 | The distribution of *mBMSCs* on Ti-Grad-1RGD. (a) Distribution (n =3), (b) average number (n=3 within a band) and (c) cell spreading area (n=10 within a band) of fluorescently stained *mBMSCs* on different bands of Ti-Grad-1RGD after 24 h of culturing. After being stained with F-actin and DAPI, the cells were observed under a fluorescence microscope through the FITC and DAPI channels (scale bar, 400 μm). Data are displayed as mean ± SD. It should be noted that 9 images were obtained along the gradient direction of one sample and combined to form the image in **a**, and the dotted lines in **a** highlight the band boundaries (but not the dividing lines of the 9 images). The actual dividing boundaries of the 9 images are marked as solid black lines at the bottom of the image in **a**. See Experimental section of "Cell assay" in the Main Text for details of the assay.

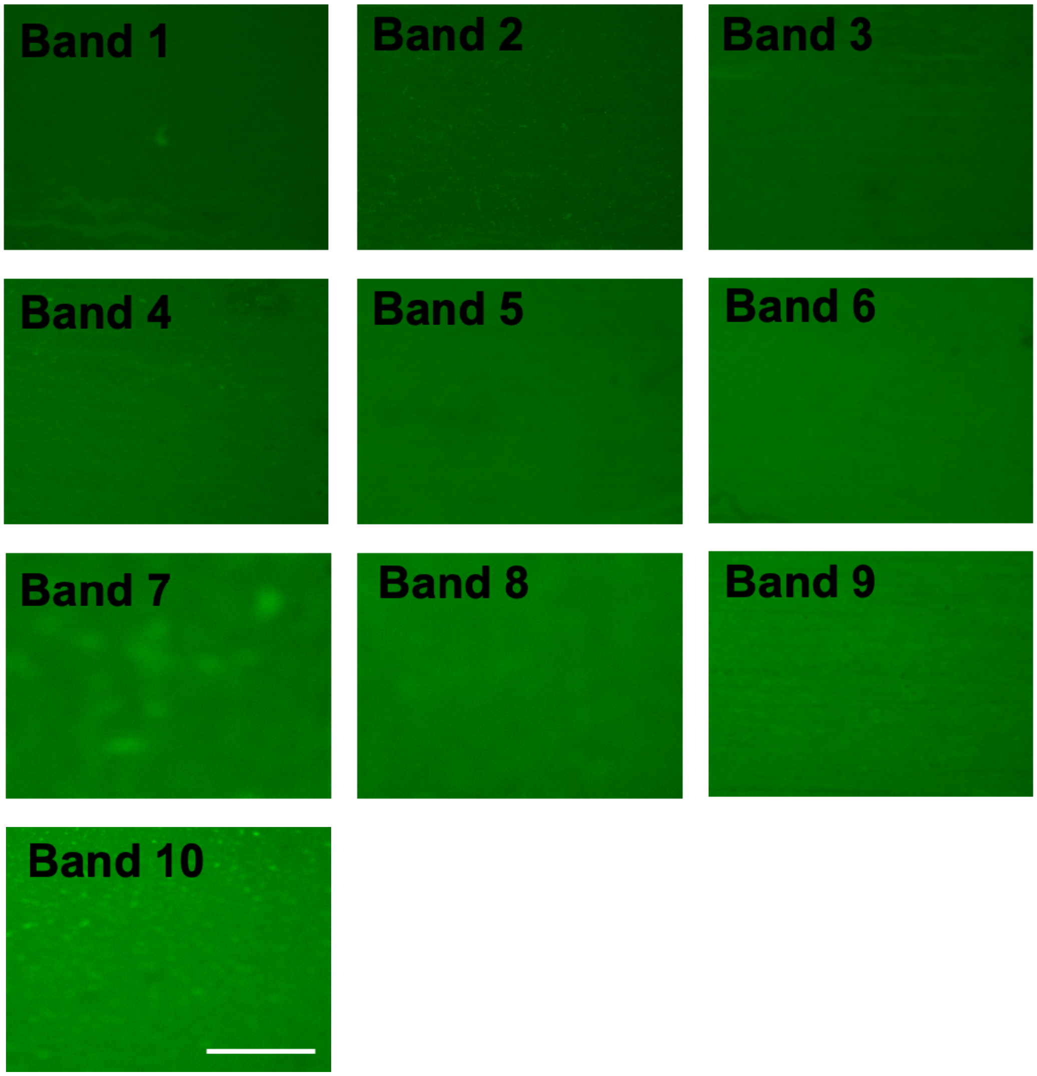

Supplementary Fig. 15 | The magnified FITC fluorescence images of different bands on Ti-Grad-20AMP (n=3, scale bar, 200 µm). See Experimental section of "Preparation of the AMP-functionalized gradient Ti surfaces" in the Main Text for details of the assay.

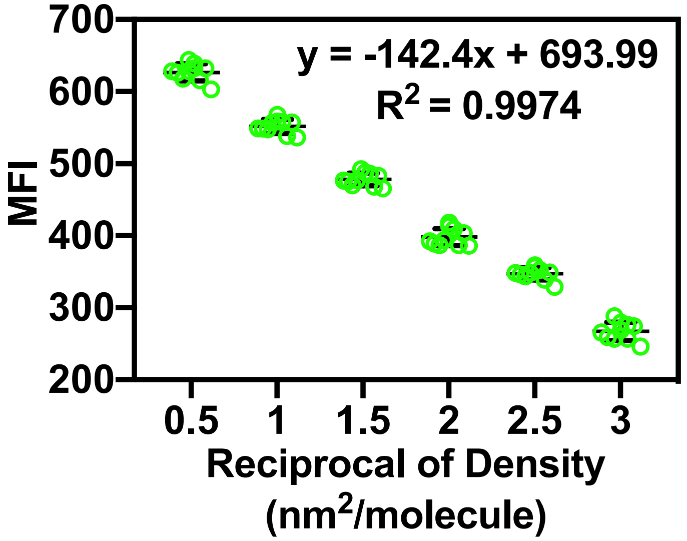

Supplementary Fig. 16 | The standard curve of MFI and the reciprocal inverse of the density of AMP-FITC on Ti-S. The curve was obtained by a fluorescence microscope through the FITC channel. On each surface, we randomly selected 10 points to calculate the MFI (n=10). There was an inverse linear relation between MFI and the reciprocal of the AMP-FITC density. Data are displayed as mean ± SD. See Experimental section of "Calculation of the density of peptide" in the Main Text for details of the assay.

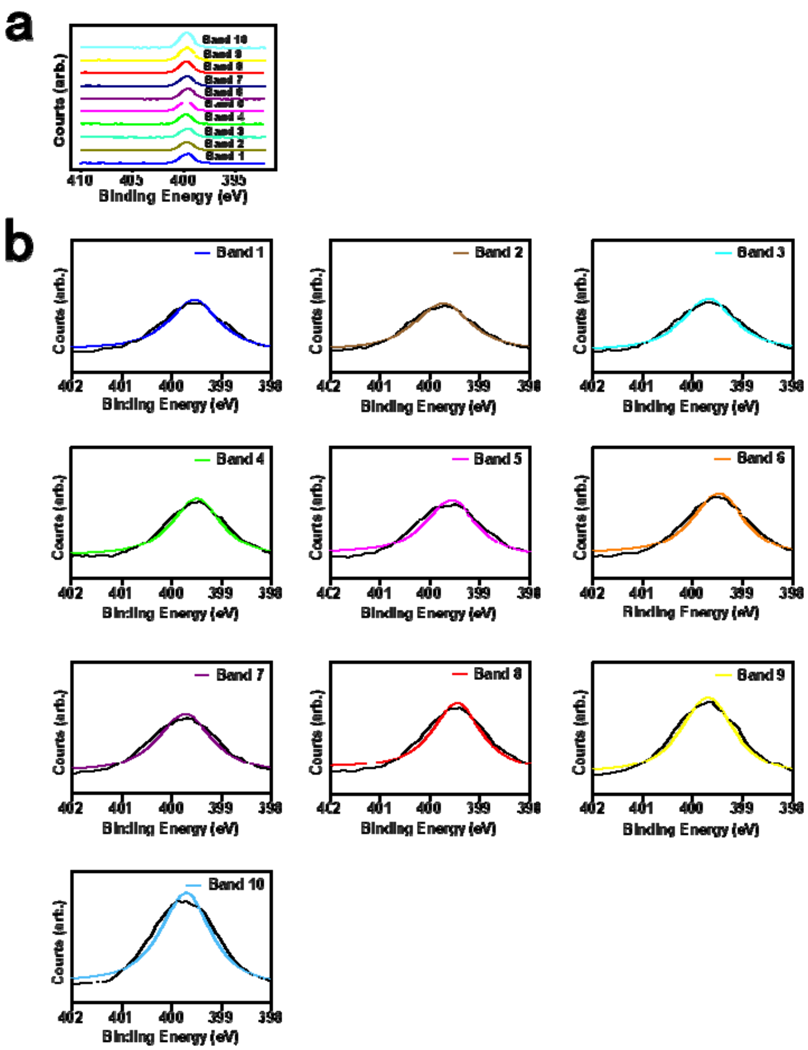

Supplementary Fig. 17 | (a) XPS N1s high-resolution spectra of the indicated bands on Ti-Grad-20AMP. (b) The enlarged XPS N1s high-resolution spectrum of each band in (a) with the binding energy from 398 eV to 402 eV was shown. See Experimental section of "X-ray photoelectron spectroscopy (XPS) assay" in the Supporting Information for details of the assay.

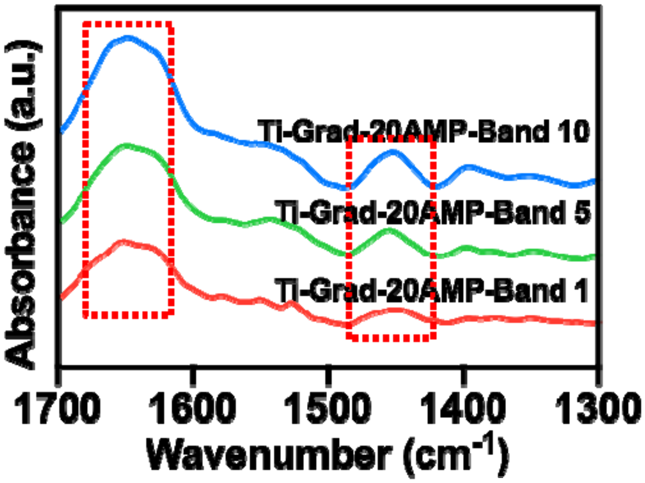

Supplementary Fig. 18 | FTIR spectra of the amide bond in the specific bands on Ti-Grad-20AMP. See Experimental section of "Fourier transform infrared spectrometer (FTIR) assay" in the Supporting Information for details of the assay.

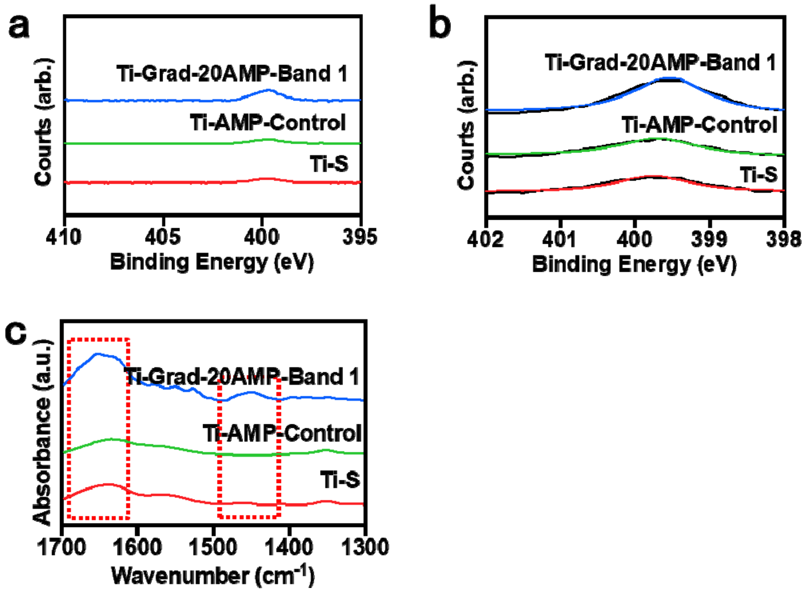

Supplementary Fig. 19 | XPS and FTIR results of different surfaces. (a) XPS N1s high-resolution spectra of Ti-S, Ti-AMP-Control and Ti-Grad-20AMP-Band 1. (b) The enlarged XPS N1s high-resolution spectrum of the indicated surfaces in (a) with the binding energy from 398 eV to 402 eV was shown. (c) FTIR spectra of the amide bond on Ti-S, Ti-AMP-Control and band 1 on Ti-Grad-20AMP. See Experimental section of "X-ray photoelectron spectroscopy (XPS) assay" and "Fourier transform infrared spectrometer (FTIR) assay" in the Supporting Information for details of the assay.

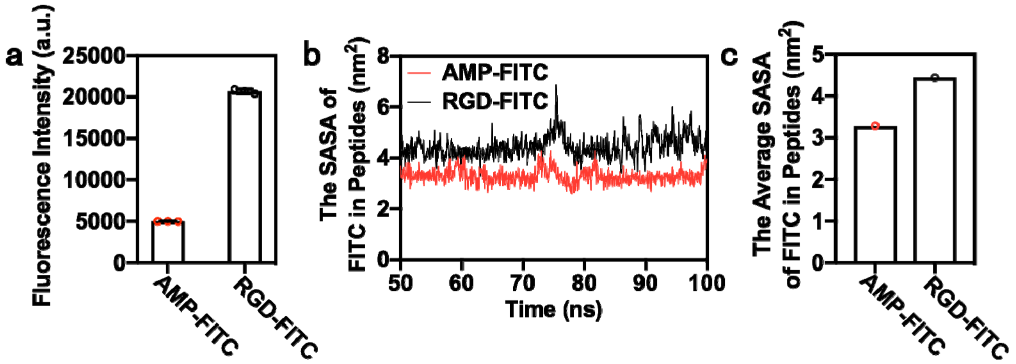

Supplementary Fig. 20 | All-atom molecular dynamic (MD) simulation of the peptides. (a) The fluorescence intensity of AMP-FITC and RGD-FITC at the same concentration in water (100 µM, n=3). Data are displayed as mean ± SD. (b) The solvent accessible surface area (SASA) of FITC and (c) the average SASA of FITC in different peptides in water. See Experimental section of "molecular dynamics simulation (MD) assay" in the Supporting Information for details of the assay.

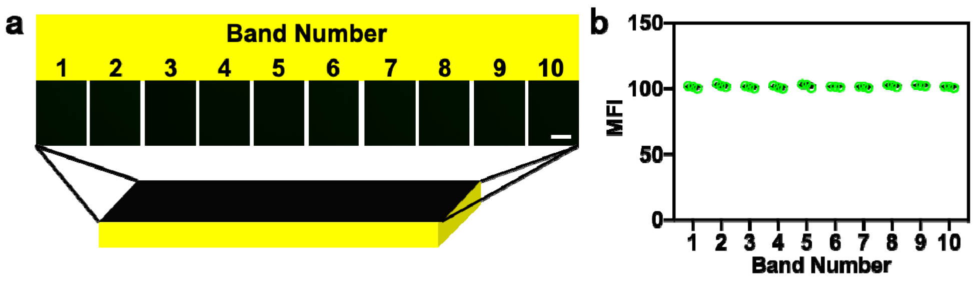

Supplementary Fig. 21 | The gradient properties of Ti-Grad-1AMP with gradient AMP density along the vertical direction. (a) The FITC fluorescence images of Ti-Grad-1AMP. The images of the 10 bands were collected individually and lined up as they were originally on the Ti substrate (n=3, scale bar, 200 μm). (b) The mean fluorescence intensity (MFI) of each band of Ti-Grad-1AMP. In each band, we randomly selected 3 points to calculate the MFI (n=3). Data are displayed as mean ± SD. See Experimental section of “Preparation of the AMP-functionalized gradient Ti surfaces” in the Main Text for details of the assay.

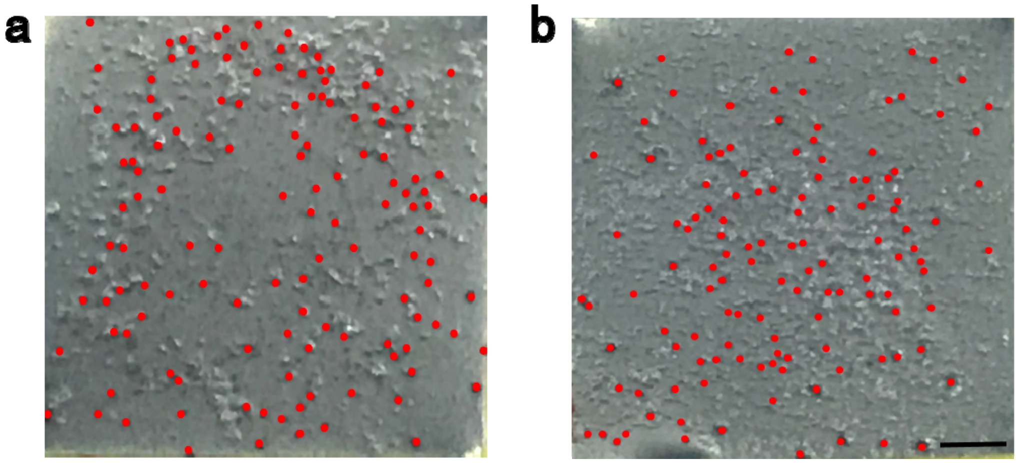

Supplementary Fig. 22 | Distribution of live bacteria (red dots, detected by Petrifilm method) on different bands of AMP-functionalized Ti surface after 24 h of culturing. (a) Ti-S and (b) Ti-Grad-1AMP (n=3, scale bar, 1.5 mm). See Experimental section of "Antimicrobial assay" in the Main Text for details of the assay.

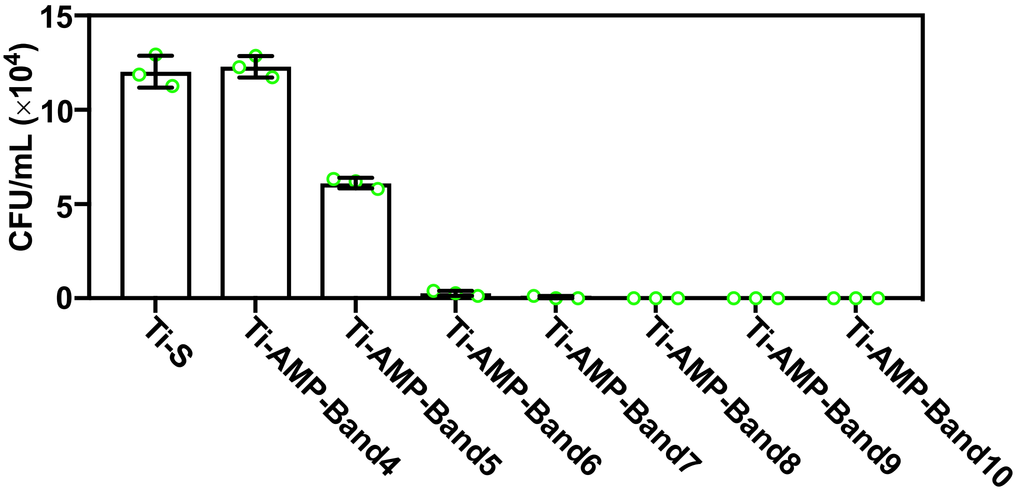

Supplementary Fig. 23 | Antimicrobial assays of the uniform middle of each band of Ti surfaces prepared from band 4 to band 10 against *S. aureus* by an agar plate method (n=3). Data are displayed as mean ± SD. See Experimental section of "Antimicrobial assay" in the Main Text for details of the assay.

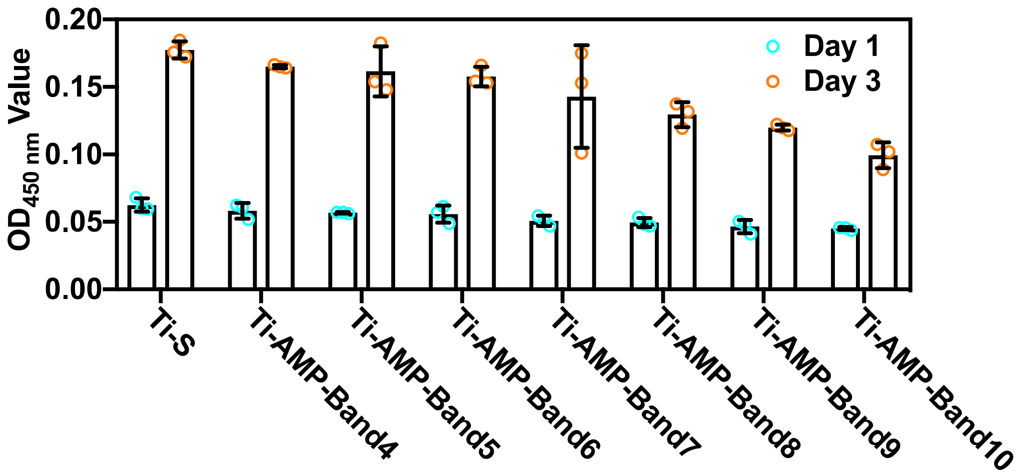

Supplementary Fig. 24 | CCK-8 results for the uniform middle of each band of Ti surfaces prepared from band 4 to band 10 with *mBMSCs* after 1 and 3 days of culturing (n=3). Data are displayed as mean ± SD. See Experimental section of "Cell assay" in the Main Text for details of the assay.

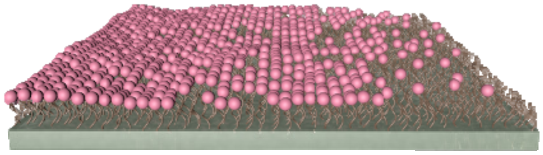

Supplementary Fig. 25 | Ti-Grad-50AMP shows a gradient in the AMP density, which would cause a gradient in the density of unconjugated MAL on this surface.

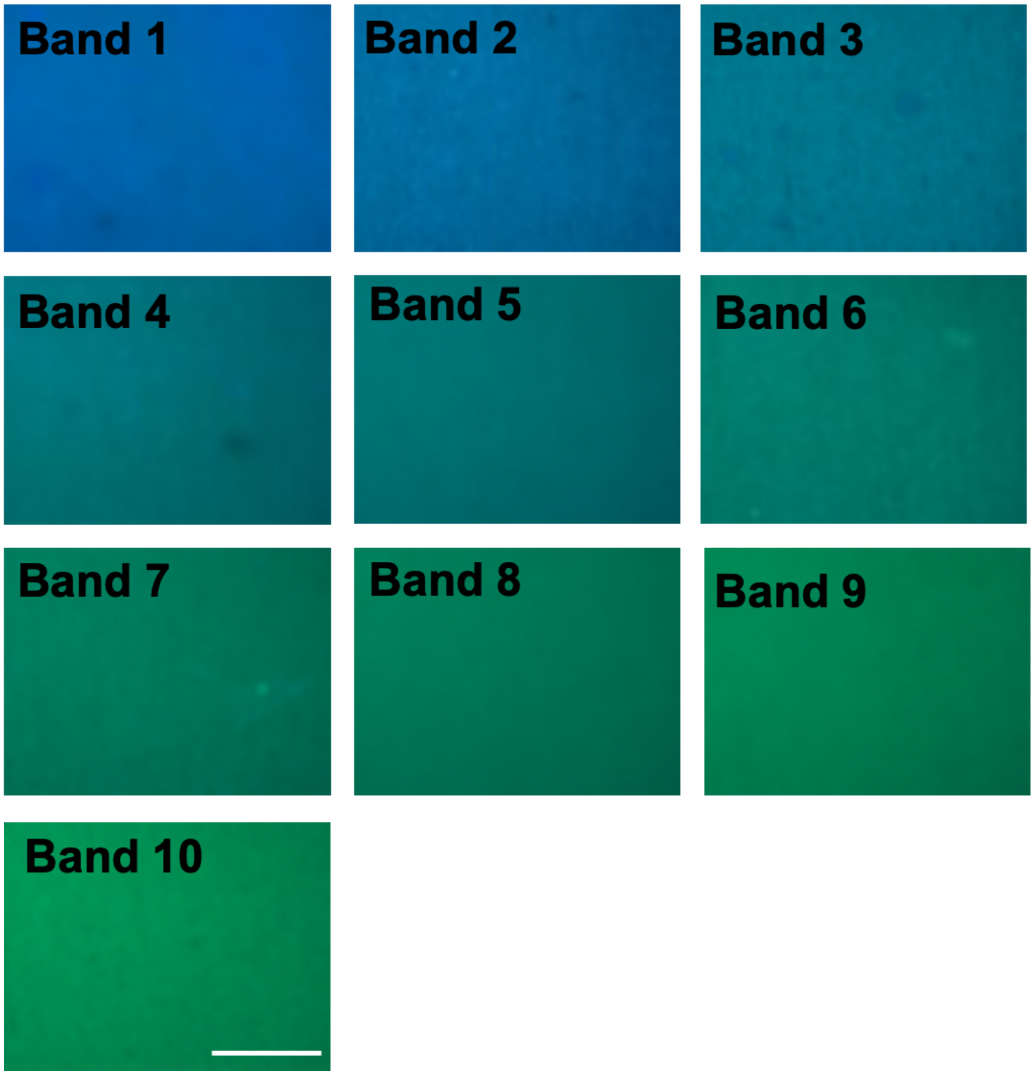

Supplementary Fig. 26 | The magnified FITC fluorescence images with AMP-FITC/RGD-Mca of different bands on Ti-Grad-Dual (n=3, scale bar, 200 μm). See Experimental section of "Preparation of the dual-functionalized gradient Ti surfaces" in the Main Text for details of the assay.

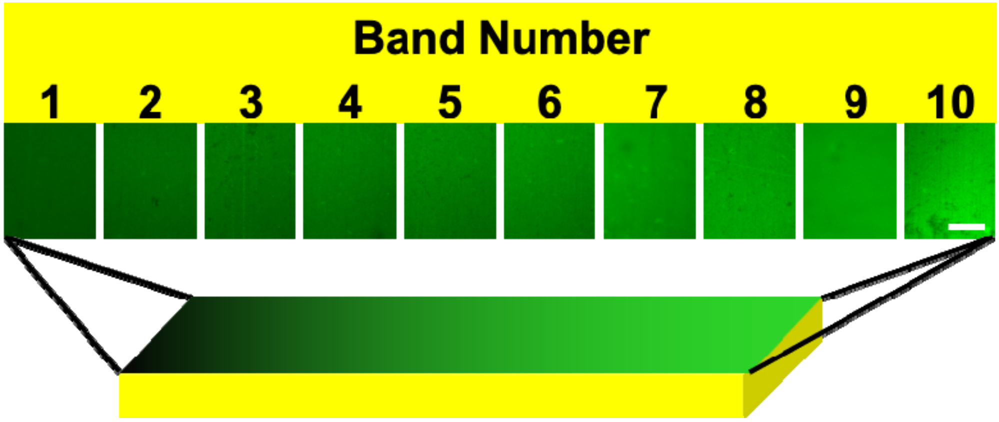

Supplementary Fig. 27 | The FITC fluorescence images of Ti-Grad-50AMP with AMP-FITC. The images of the 10 bands were collected individually and lined up as they were originally on the Ti substrate (n=3, scale bar, 200 μm). See Experimental section of "Preparation of the AMP-functionalized gradient Ti surface" in the Main Text for details of the assay.

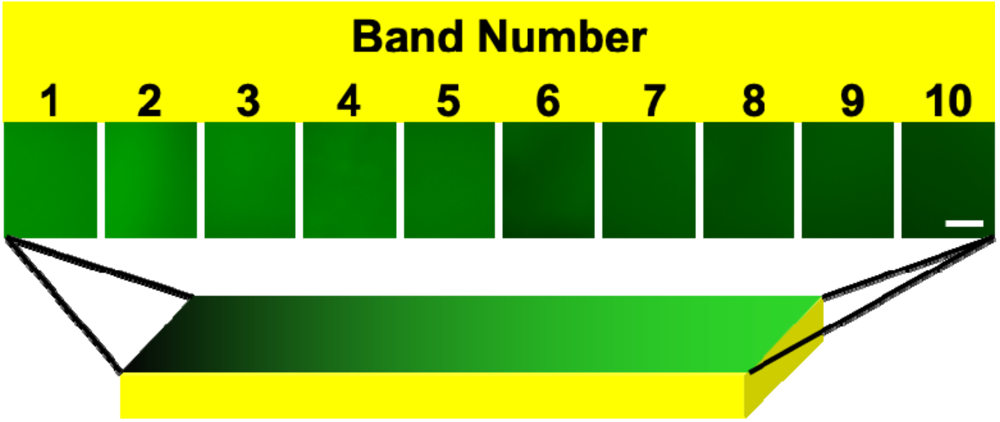

Supplementary Fig. 28 | The FITC fluorescence images of Ti-Grad-50Dual with the combination of AMP/RGD-FITC. The images of the 10 bands were collected individually and lined up as they were originally on the Ti substrate (n=3, scale bar, 200 μm). See Experimental section of "Preparation of the dual-functionalized gradient Ti surfaces" in the Main Text for details of the assay.

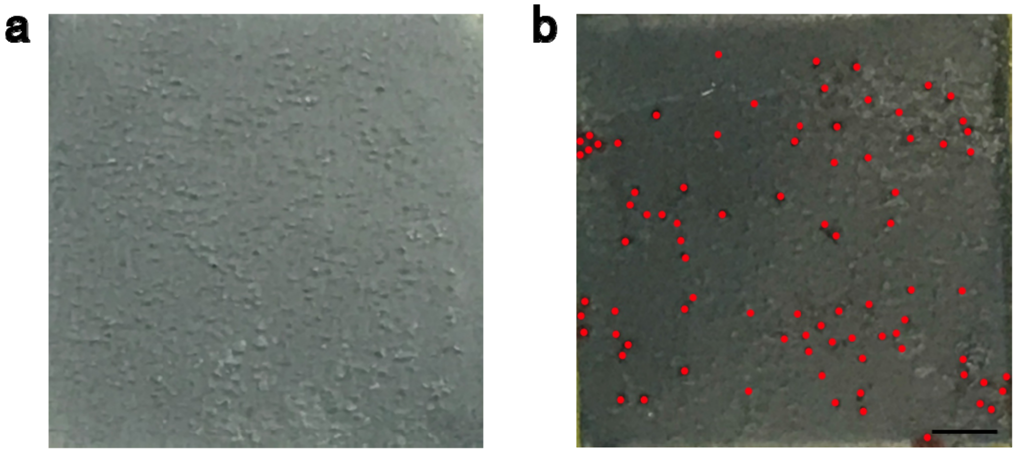

Supplementary Fig. 29 | Distribution of live bacteria (red dots, detected by Petrifilm method) on different bands of AMP-functionalized Ti surfaces after 24 h of culturing. (a) Ti-Grad-50AMP and (b) Ti-Grad-20Dual (n=3, scale bar, 1.5 mm). See Experimental section of "Antimicrobial assay" in the Main Text for details of the assay.

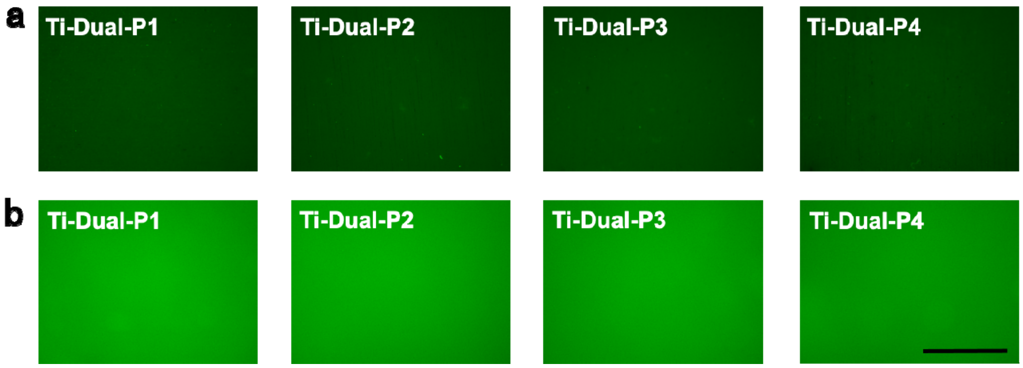

Supplementary Fig. 30 | The FITC fluorescence images of the dual-functionalized uniform Ti surfaces. (a) The indicated Ti surfaces with AMP-FITC. (b) The indicated Ti surfaces with the combination of AMP/RGD-FITC (scale bar, 500 μm). The images were obtained by fluorescence microscope under the FITC channel. For each sample, 5 images were chosen randomly to calculate the MFI (n=5). See Experimental section of "Extraction of the parameters from the specific site on gradient surface for uniform surfaces" in the Main Text for details of the assay.

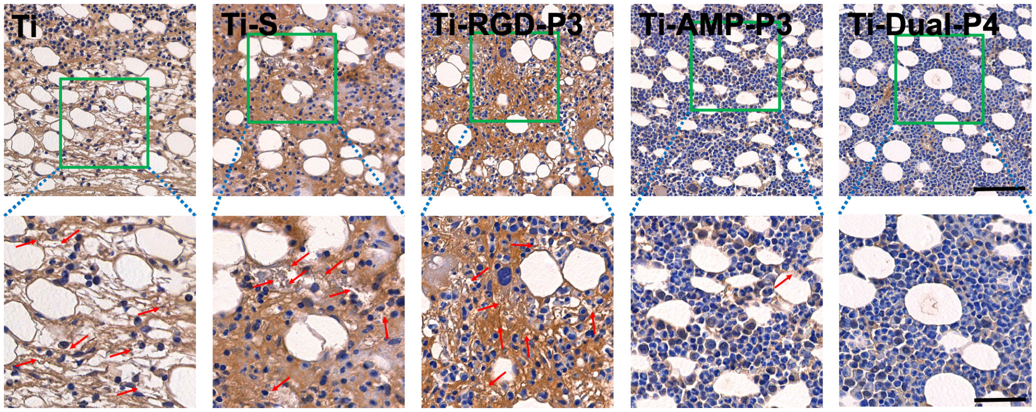

Supplementary Fig. 31 | The immunohistochemistry staining images of the bone tissues. The *S. aureus* bacteria were pointed out by the red arrows. The scale bars before and after enlargement (20× and 40× magnification) were 80 and 10 μm, respectively (n=3). See Experimental section of "In vivo assay" in the Main Text for details of the assay.

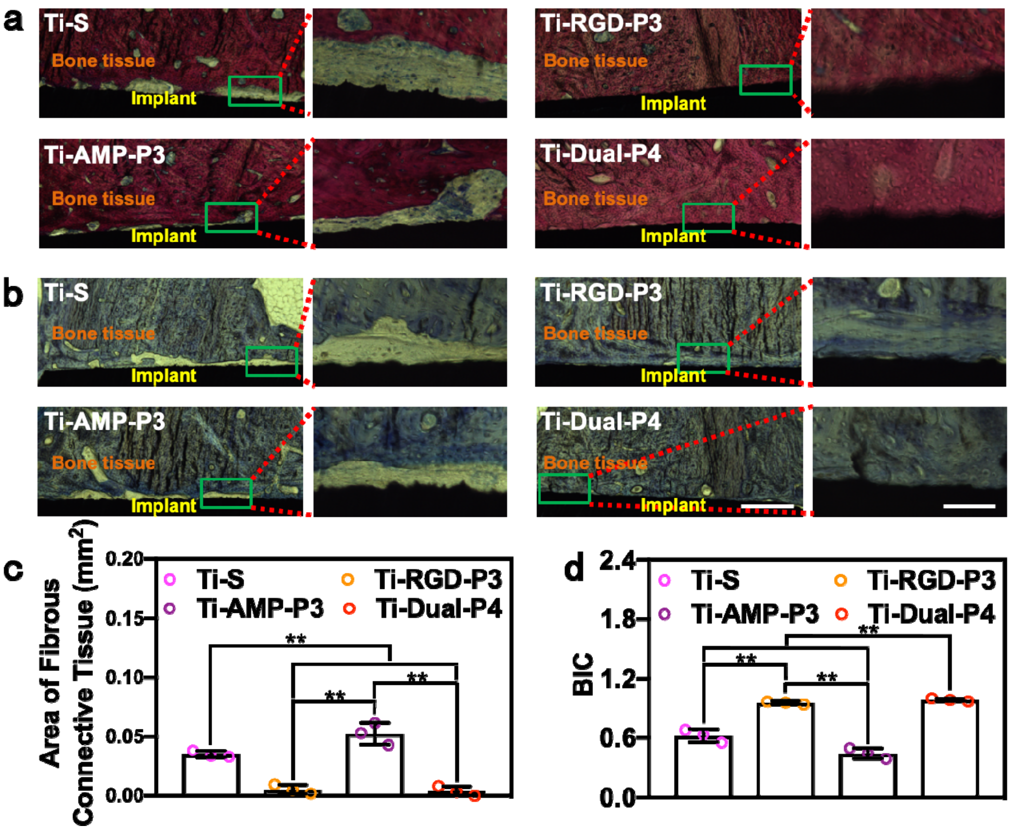

Supplementary Fig. 32 | In vivo osteogenesis assay in the non-infection model. (a) The methylene blue & basic fuchsin (n=3) and (b) toluidine blue staining (n=3) images of the hard tissue section of implantation. The interface between the implant and tissue in the green rectangle was enlarged (the scale bars before and after enlargement (10× and 20× magnification) were 200 and 100 µm, respectively). Quantitative analysis of methylene blue & basic fuchsin staining: (c) area of fibrous connective tissue and (d) BIC at the interface between bone tissue and implant. Three sections in each group were chosen for the analysis (n = 3). (Sidak's multiple comparisons test, two-way ANOVA. In figure c: Ti-S vs Ti-RGD-P3, **p=0.0004; Ti-S vs Ti-Dual-P4, **p=0.0004; Ti-RGD-P3 vs Ti-AMP-P3, **p = 0.0012; Ti-AMP-P3 vs Ti-Dual-P4, **p = 0.0011. In figure d: Ti-S vs Ti-RGD-P3, **p = 0.0010; Ti-S vs Ti-Dual-P4, **p=0.0007; Ti-RGD-P3 vs Ti-AMP-P3, **p < 0.0010; Ti-AMP-P3 vs Ti-Dual-P4, **p < 0.0001). Data are displayed as mean ± SD. See Experimental section of "In vivo assay" in the Main Text for details of the assay.

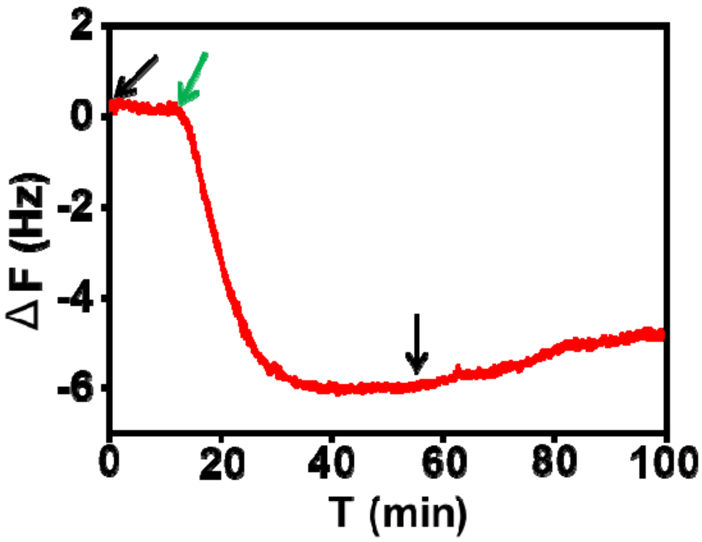

Supplementary Fig. 33 | QCM-D assay of the integration of RGD on the Au substrate. The black arrows indicated the injection of ethanol, and the green arrow indicated the injection of 50 μM RGD solution in ethanol. See Experimental section of "Quartz crystal microbalance with dissipation (QCM-D) assay" in the Supporting Information for details of the assay.

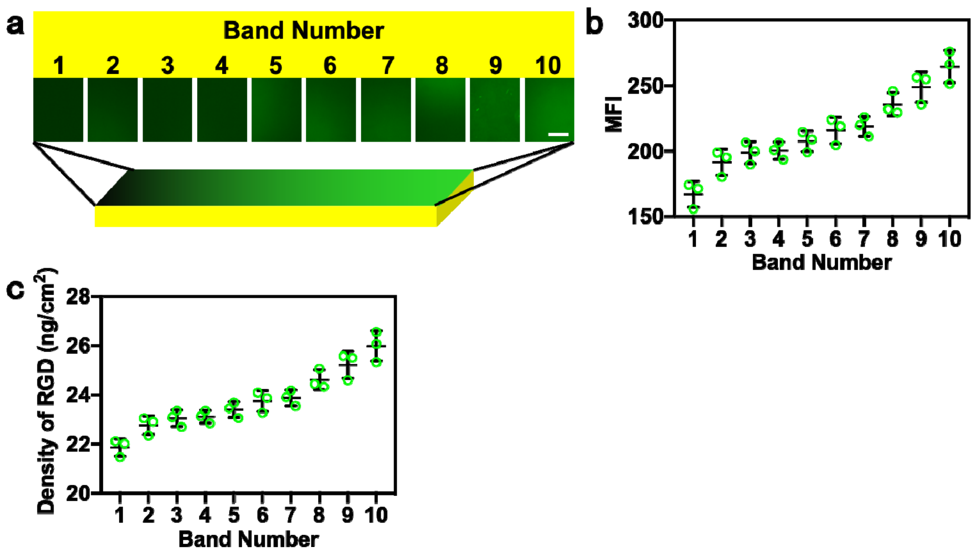

Supplementary Fig. 34 | The gradient properties of Au-Grad-0.1RGD with gradient RGD density along the vertical direction. (a) The images of the 10 bands were collected individually and lined up as they were originally on the Au substrate (n=3, scale bar, 200 μm). (b) The mean fluorescence intensity (MFI) and (c) the RGD density (calculated by the fluorescence method[11]) of each band of Au-Grad-0.1RGD. In each band, we randomly selected 3 points to calculate the MFI (n=3). Data are displayed as mean ± SD. See Experimental section of "Preparation of the RGD-functionalized gradient Au surfaces" in the Supporting Information and "Calculation of the density of peptide" in the Main Text for details of the assay.

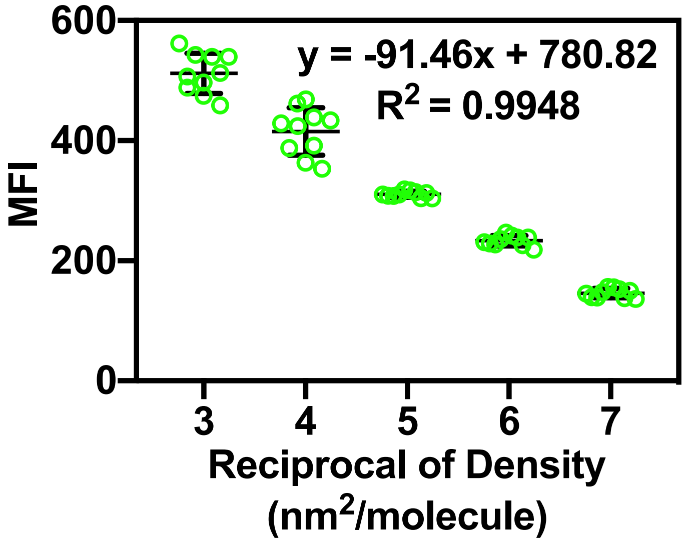

Supplementary Fig. 35 | The standard curve of MFI and the reciprocal inverse of the density of RGD-FITC on the Au substrates. The curve was obtained by fluorescence microscope under the FITC channel. On each surface, we randomly selected 10 points to calculate the MFI (n=10). There was an inverse linear relation between MFI and the reciprocal of the RGD-FITC density. Data are displayed as mean ± SD. See Experimental section of "Calculation of the density of peptide" in the Main Text for details of the assay.

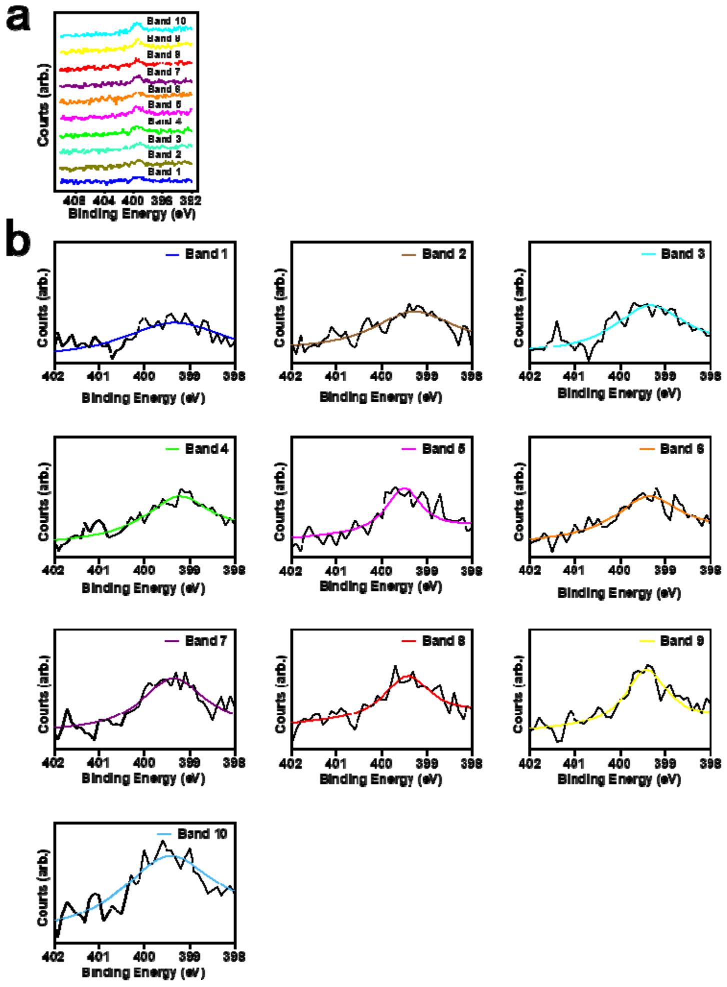

Supplementary Fig. 36 | (a) XPS N1s high-resolution spectra of the indicated bands on Au-Grad-0.1RGD. (b) The enlarged XPS N1s high-resolution spectrum of each band with the binding energy from 398 eV to 402 eV was shown. See Experimental section of "X-ray photoelectron spectroscopy (XPS) assay" in the Supporting Information for details of the assay.

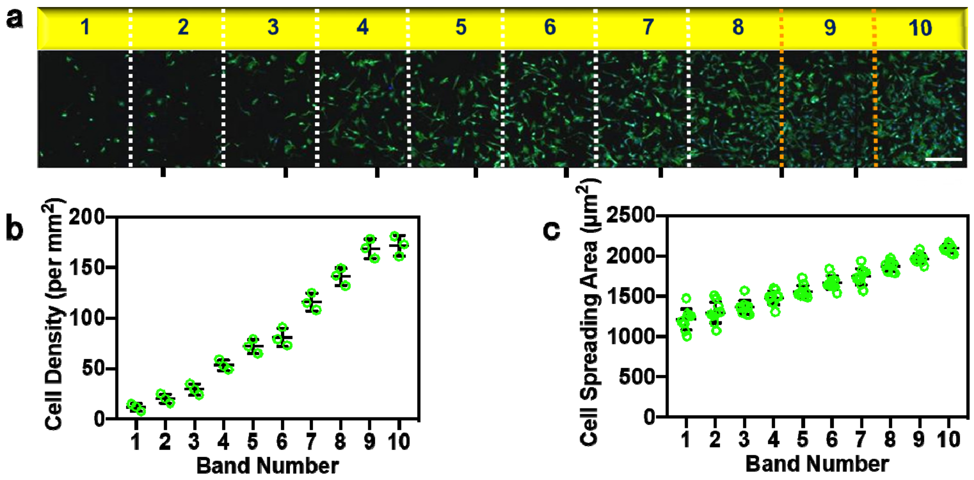

Supplementary Fig. 37 | The distribution of *mBMSCs* on Au-Grad-0.1RGD. (a) Distribution (n=3), (b) average number (n=3 within a band) and (c) cell spreading area (n=10 within a band) of fluorescently stained *mBMSCs* on different bands of Au-Grad-0.1RGD after 24 h of culturing. After being stained with F-actin and DAPI, the cells were observed by fluorescence microscope under the FITC and DAPI channels (scale bar, 400 μm). Data are displayed as mean ± SD. It should be noted that 9 images were obtained along the gradient direction of one sample and combined to form the image in **a**, and the dotted lines in **a** highlight the band boundaries (but not the dividing lines of the 9 images). The actual dividing boundaries of the 9 images are marked as solid black lines at the bottom of the image in **a**. See Experimental section of "Cell assay" in the Main Text for details of the assay.

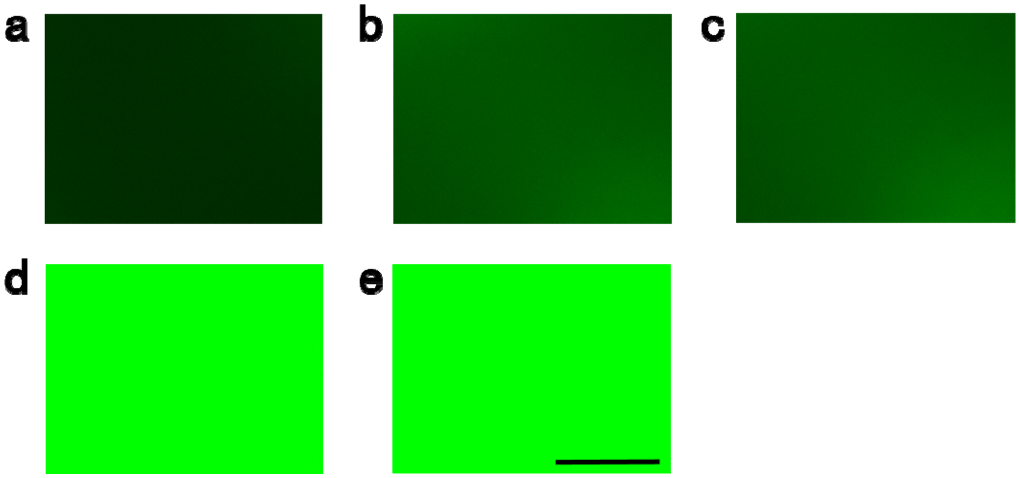

Supplementary Fig. 38 | The fluorescence images of the RGD-functionalized uniform Au surfaces. (a) Au-RGD-P1, (b) Au-RGD-P2, (c) Au-RGD-P3, (d) Au-RGD-P4 and (e) Au-RGD-P5. The images were obtained by fluorescence microscope under the FITC channel. For each sample, 5 images were chosen randomly to calculate the MFI (n=5, scale bar, 500 μm). See Experimental section of "Extraction of the parameters from the specific site on gradient surface for uniform surfaces" in the Main Text for details of the assay.

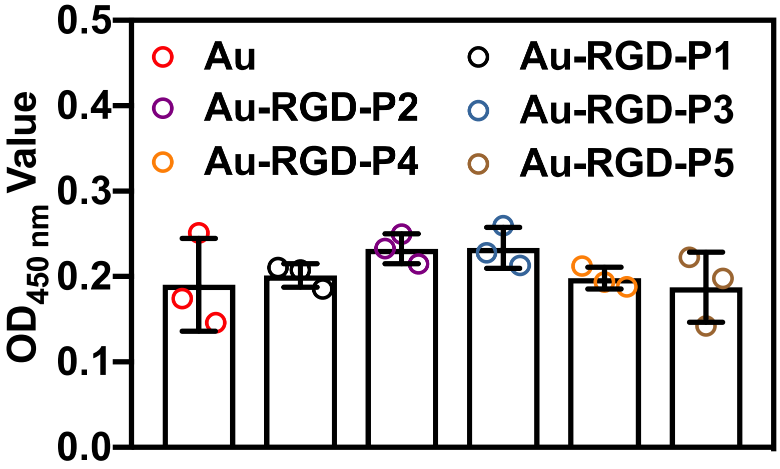

Supplementary Fig. 39 | CCK-8 results for the indicated uniform Au surface with *mBMSCs* after 3 days of culturing (n=3). Data are displayed as mean ± SD. See Experimental section of "Cell assay" in the Main Text for details of the assay.

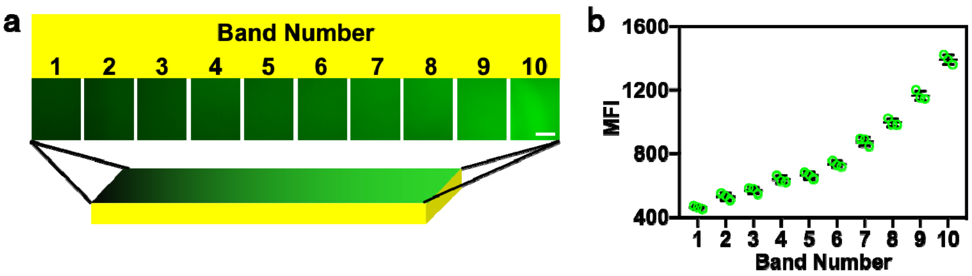

Supplementary Fig. 40 | The gradient properties of Au-Grad-1RGD with gradient RGD density along the vertical direction. (a) The FITC fluorescence images of Au-Grad-1RGD. The images of the 10 bands were collected individually and lined up as they were originally on the Au substrate (n=3, scale bar, 200 μm). (b) The mean fluorescence intensity (MFI) of each band of Au-Grad-1RGD. In each band, we randomly selected 3 points to calculate the MFI (n=3). Data are displayed as mean ± SD. See Experimental section of "Preparation of the RGD-functionalized gradient Au surface" in the Supporting Information for details of the assay.

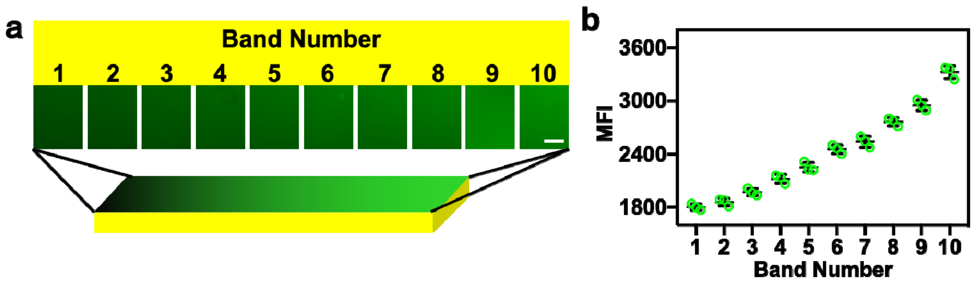

Supplementary Fig. 41 | The gradient properties of Au-Grad-10RGD with gradient RGD density along the vertical direction. (a) The FITC fluorescence images of Au-Grad-10RGD. The images of the 10 bands were collected individually and lined up as they were originally on the Au substrate (n=3, scale bar, 200 μm). (b) The mean fluorescence intensity (MFI) of each band of Au-Grad-10RGD. In each band, we randomly selected 3 points to calculate the MFI (n=3). Data are displayed as mean ± SD. See Experimental section of "Preparation of the RGD-functionalized gradient Au surface" in the Supporting Information for details of the assay.

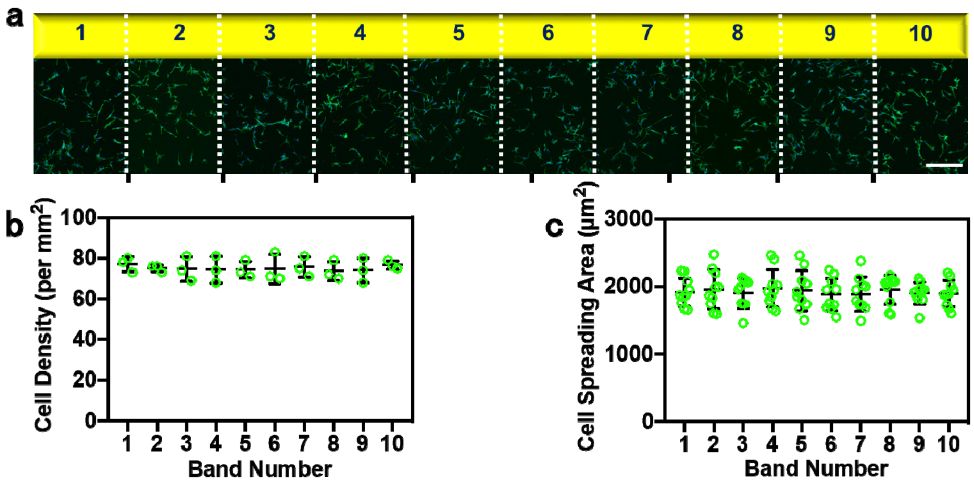

Supplementary Fig. 42 | The distribution of *mBMSCs* on Au-Grad-1RGD. (a) Distribution (n =3), (b) average number (n=3 within a band) and (c) cell spreading area (n = 10 within a band) of fluorescently stained *mBMSCs* on different bands of Au-Grad-1RGD after 24 h of culturing. After being stained with F-actin and DAPI, the cells were observed by fluorescence microscope under the FITC and DAPI channels (scale bar, 400 μm). Data are displayed as mean ± SD. It should be noted that 9 images were obtained along the gradient direction of one sample and combined to form the image in **a**, and the dotted lines in **a** highlight the band boundaries (but not the dividing lines of the 9 images). The actual dividing boundaries of the 9 images are marked as solid black lines at the bottom of the image in **a**. See Experimental section of "Cell assay" in Main Text for details of the assay.

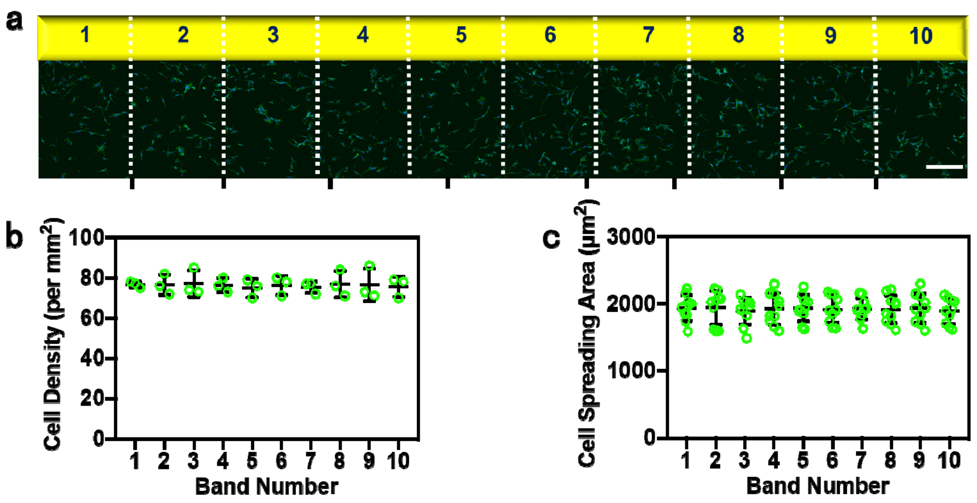

Supplementary Fig. 43 | The distribution of *mBMSCs* on Au-Grad-10RGD. (a) Distribution (n=3), (b) average number (n=3 within a band) and (c) cell spreading area (n=10 within a band) of fluorescently stained *mBMSCs* on different bands of Au-Grad-10RGD after 24 h of culturing. After being stained with F-actin and DAPI, the cells were observed by fluorescence microscope under the FITC and DAPI channels (scale bar, 400 μm). Data are displayed as mean ± SD. It should be noted that 9 images were obtained along the gradient direction of one sample and combined to form the image in **a**, and the dotted lines in **a** highlight the band boundaries (but not the dividing lines of the 9 images). The actual dividing boundaries of the 9 images are marked as solid black lines at the bottom of the image in **a**. See Experimental section of "Cell assay" in Main Text for details of the assay.

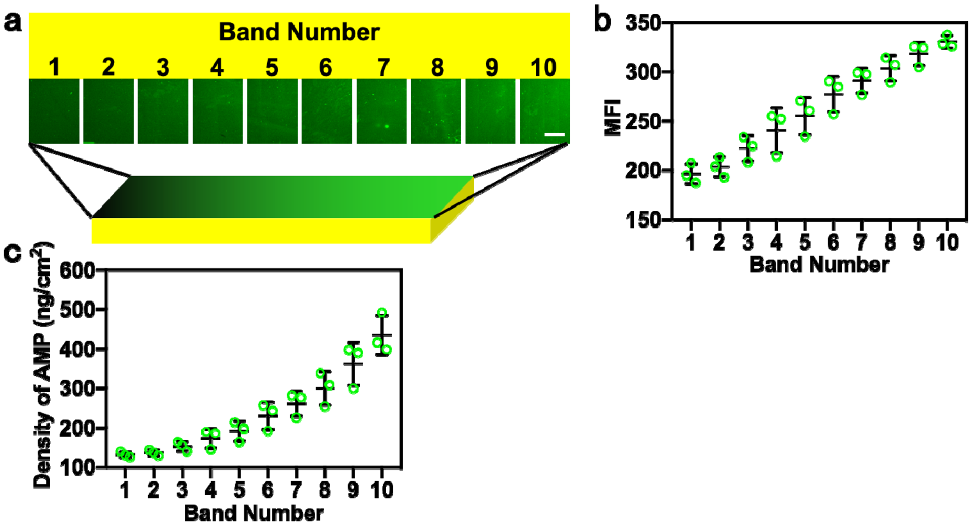

Supplementary Fig. 44 | The gradient properties of Au-Grad-50AMP with gradient AMP density along the vertical direction. (a) The FITC fluorescence images of Au-Grad-50AMP. The images of the 10 bands were collected individually and lined up as they were originally on the Au substrate (n=3, scale bar, 200 μm). (b) The MFI and (c) the AMP density (calculated by the fluorescence method[11]) of each band of Ti-Grad-50AMP. In each band, we randomly selected 3 points to calculate the MFI (n=3). Data are displayed as mean ± SD. See Experimental section of "Preparation of the AMP-functionalized gradient Au surfaces" in Supporting Information and "Calculation of the density of peptide" in the Main Text for details of the assay.

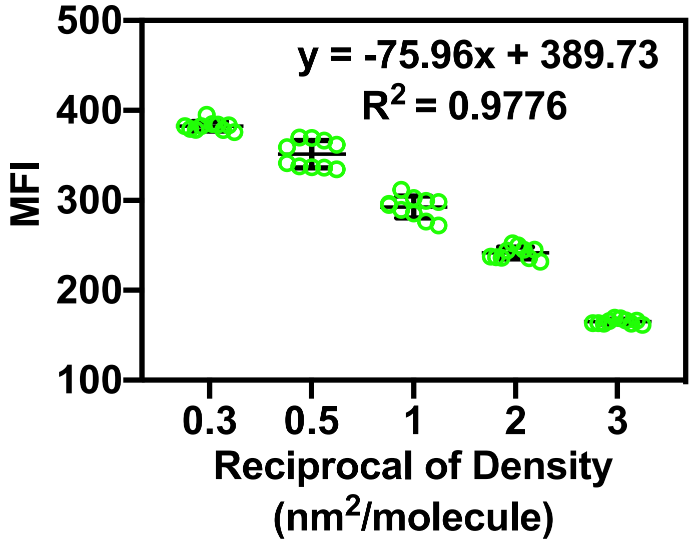

Supplementary Fig. 45 | The standard curve of MFI and the reciprocal inverse of the density of AMP-FITC on the Au substrates. The curve was obtained by fluorescence microscope under the FITC channel. On each surface, we randomly selected 10 points to calculate the MFI (n=10). There was an inverse linear relation between MFI and the reciprocal of the AMP-FITC density. Data are displayed as mean ± SD. See Experimental section of "Calculation of the density of peptide" in the Main Text for details of the assay.

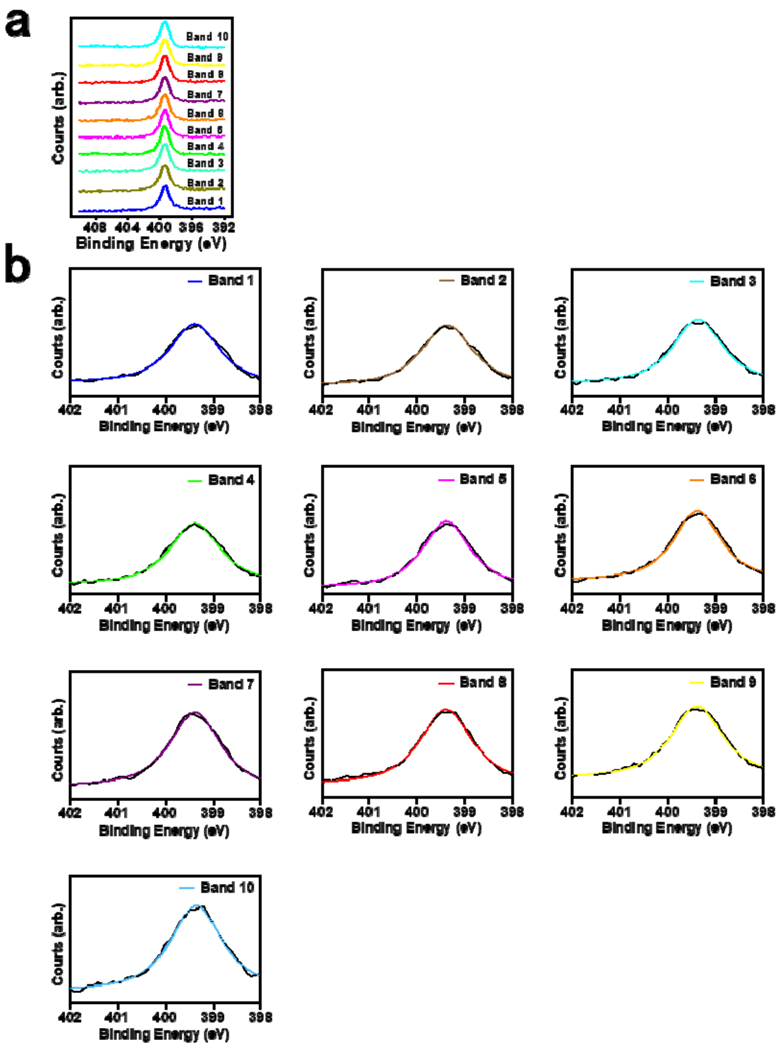

Supplementary Fig. 46 | (a) XPS N1s high-resolution spectra of the indicated bands on Au-Grad-50AMP. (b) The enlarged XPS N1s high-resolution spectrum of each band in (a) with the binding energy from 398 eV to 402 eV was shown. See Experimental section of "X-ray photoelectron spectroscopy (XPS) assay" in Supporting Information for details of the assay.

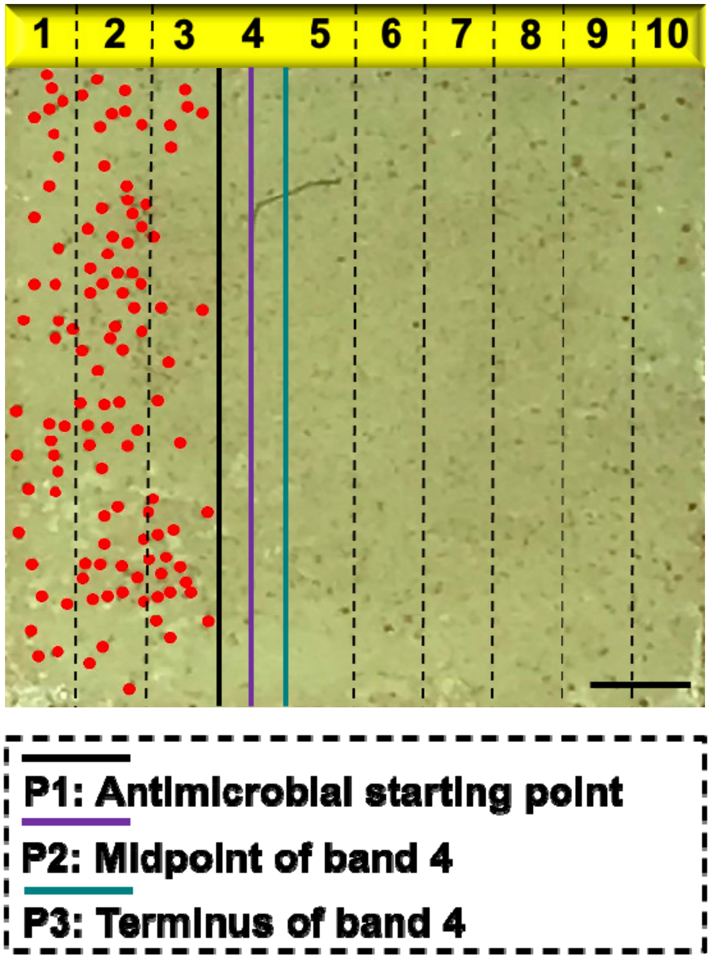

Supplementary Fig. 47 | Distribution of live bacteria (red dots, detected by the Petrifilm method) on the different bands of Au-Grad-50AMP after 24 h of culturing. Three lines were denoted, including P1, P2, P3, corresponding to the boundary between the areas without and with dead bacteria, midpoint position within band 4, and starting boundary of band 5, respectively (n=3, scale bar, 1.5 mm). See Experimental section of "Antimicrobial assay" in Main Text for details of the assay.

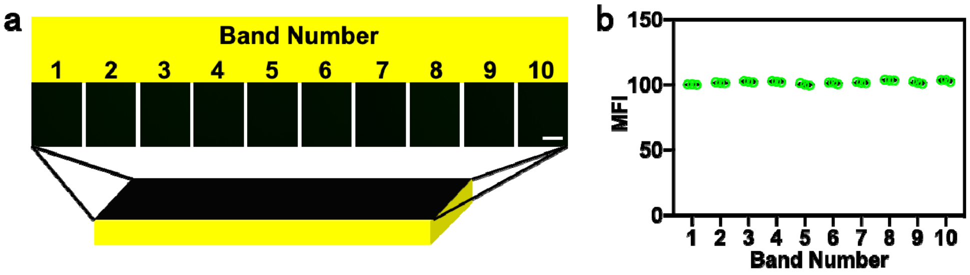

Supplementary Fig. 48 | The gradient properties of Au-Grad-5AMP with gradient AMP density along the vertical direction. (a) The FITC fluorescence images of Au-Grad-5AMP. The images of the 10 bands were collected individually and lined up as they were originally on the Au substrate (n=3, scale bar, 200 μm). (b) The mean fluorescence intensity (MFI) of each band of Au-Grad-5AMP. In each band, we randomly selected 3 points to calculate the MFI (n=3). Data are displayed as mean ± SD. See Experimental section of "Preparation of the AMP-functionalized gradient Au surfaces" in Supporting Information for details of the assay.

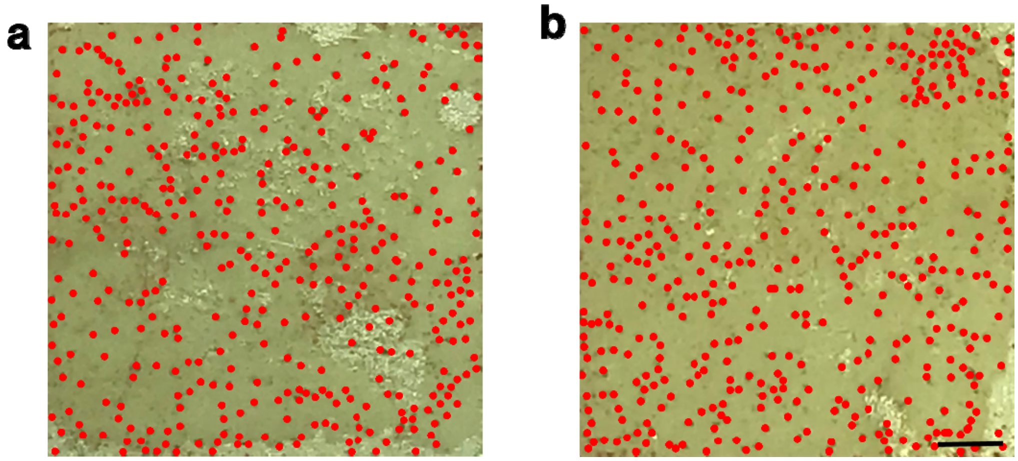

Supplementary Fig. 49 | Distribution of live bacteria (red dots, detected by the Petrifilm method) on the different bands of AMP-functionalized Au surfaces after 24 h of culturing. (a) Au and (b) Au-Grad-5AMP (n=3, scale bar, 1.5 mm). See Experimental section of "Antimicrobial assay" in the Main Text for details of the assay.

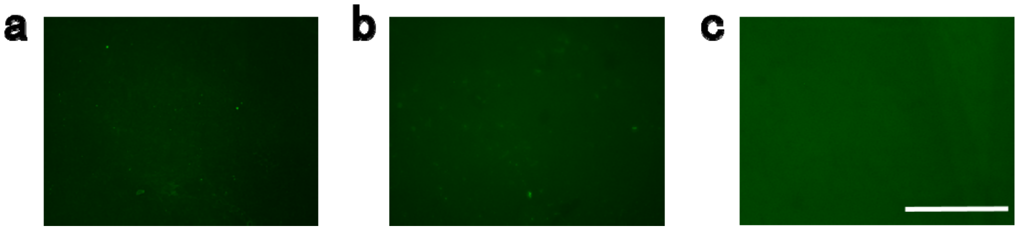

Supplementary Fig. 50 | The fluorescence images of the AMP-functionalized uniform Au surfaces. (a) Au-AMP-P1. (b) Au-AMP-P2 and (c) Au-AMP-P3. The images were obtained by fluorescence microscope under the FITC channel. For each sample, 5 images were chosen randomly to calculate the MFI (n=5, scale bar, 500 μm). See Experimental section of "Extraction of the parameters from the specific site on gradient surface for uniform surfaces" in the Main Text for details of the assay.

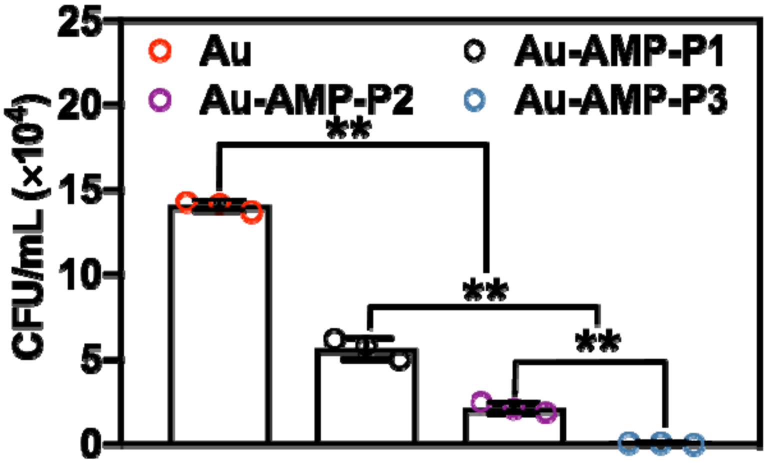

Supplementary Fig. 51 | Antimicrobial assay of the indicated uniform Au surface against *S. aureus* by an agar plate method (n=3). (Sidak's multiple comparisons test, two-way ANOVA. Au vs Au-AMP-P1, **$p < 0.0001$; Au vs Au-AMP-P2, **$p < 0.0001$; Au vs Au-AMP-P3, **$p < 0.0001$; Au-AMP-P1 vs Au-AMP-P2, **$p = 0.0009$; Au-AMP-P1 vs Au-AMP-P3, **$p < 0.0001$; Au-AMP-P2 vs Au-AMP-P3, **$p = 0.0003$). Data are displayed as mean ± SD. See Experimental section of "Antimicrobial assay" in the Main Text for details of the assay.

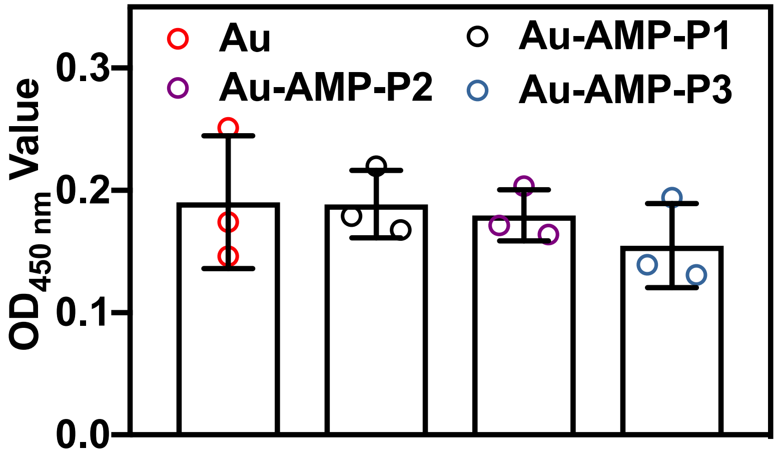

Supplementary Fig. 52 | CCK-8 results of the indicated uniform Au surfaces with *mBMSCs* after 3 days of culturing (n = 3). See Experimental section of “Cell assay” in the Main Text for details of the assay.

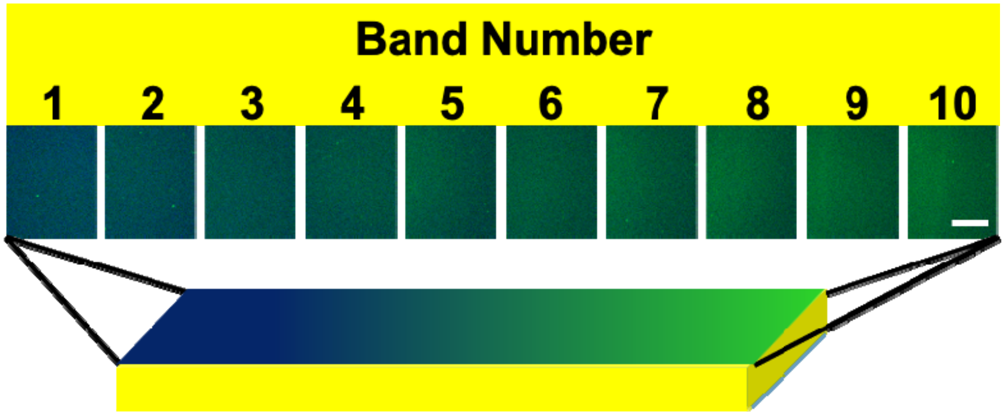

Supplementary Fig. 53 | The FITC fluorescence images of Au-Grad-50AMP with the combination of AMP-FITC/RGD-Mca. The images of the 10 bands were collected individually and lined up as they were originally on the Au substrate (n=3, scale bar, 200 μm). See Experimental section of “Preparation of the dual-functionalized gradient Au surfaces” in the Supporting Information for details of the assay.

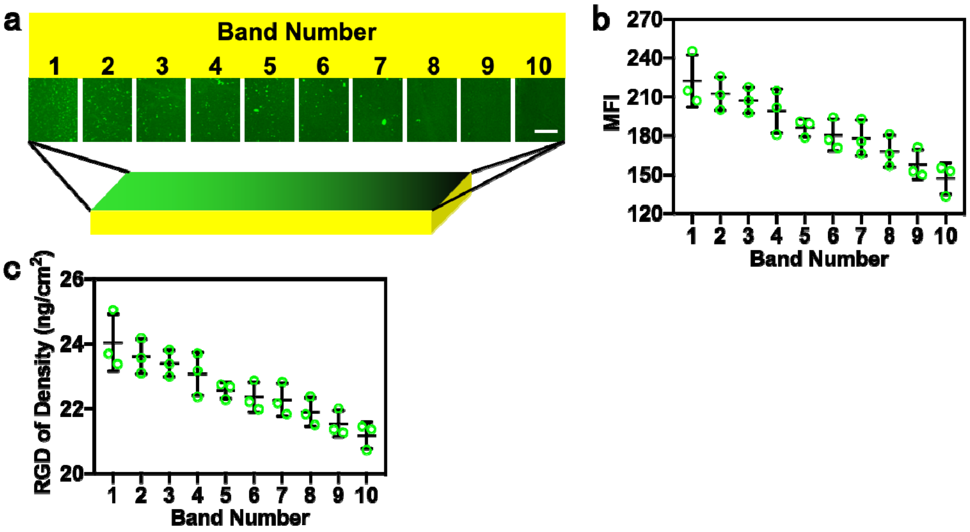

Supplementary Fig. 54 | The gradient properties of Au-Grad-Dual with the combination of AMP/RGD-FITC. (a) The FITC fluorescence images of Au-Grad-Dual with the combination of AMP/RGD-FITC. The images of the 10 bands were collected individually and lined up as they were originally on the Au substrate (n=3, scale bar, 200 μm). (b) The mean fluorescence intensity (MFI) and (c) the RGD density (calculated by the fluorescence method[11]) of each band of Au-Grad-Dual. In each band, we randomly selected 3 points to calculate the MFI (n=3). Data are displayed as mean ± SD. See Experimental section of "Preparation of the dual-functionalized gradient Au surfaces" in the Supporting Information and "Calculation of the peptide density" in the Main Text for details of the assay.

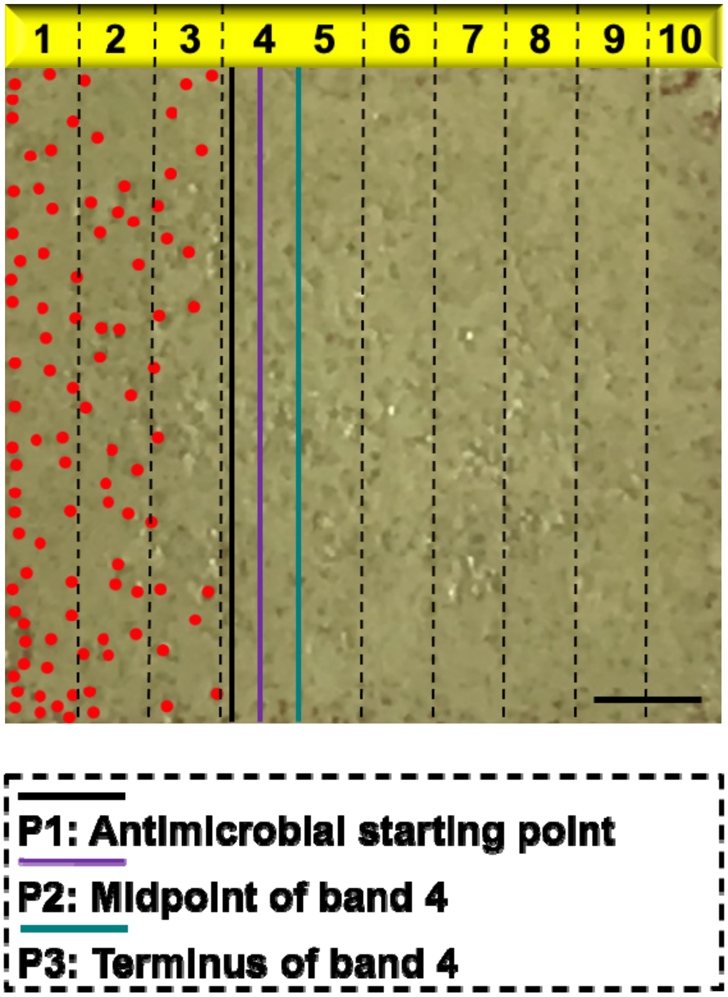

Supplementary Fig. 55 | Distribution of live bacteria (red dots, detected by the Petrifilm method) on the different bands of Au-Grad-Dual after 24 h of culturing. The line was denoted as P3 corresponding to the starting boundary of band 5 (n=3, scale bar, 1.5 mm). See Experimental section of "Antimicrobial assay" in the Main Text for details of the assay.

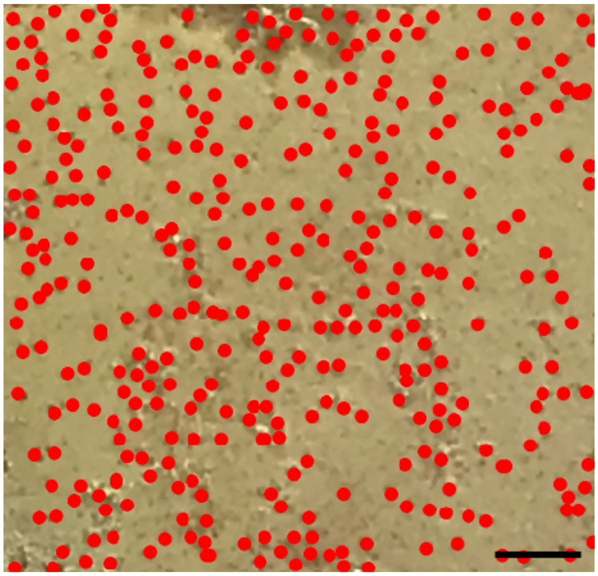

Supplementary Fig. 56 | Distribution of live bacteria (red dots, detected by the Petrifilm method) on the different bands of Au-Grad-5Dual after 24 h of culturing (n=3, scale bar, 1.5 mm). See Experimental section of "Antimicrobial assay" in the Main Text for details of the assay.

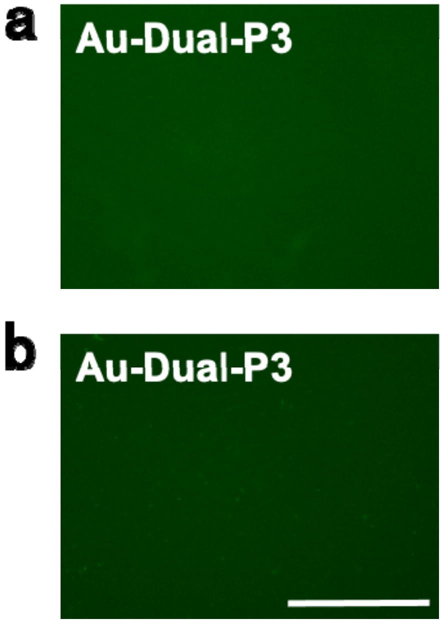

Supplementary Fig. 57 | The fluorescence images of the dual-functionalized uniform surfaces. (a) The indicated Au surfaces with AMP-FITC. (b) The indicated Au surfaces with the combination of AMP/RGD-FITC. The images were obtained by fluorescence microscope under the FITC channel (scale bar, 500 μm). The MFI of each surface was calculated by randomly selecting 5 images (n=5). See Experimental section of "Extraction of the parameters from the specific site on gradient surface for uniform surfaces" in the Main Text for details of the assay.

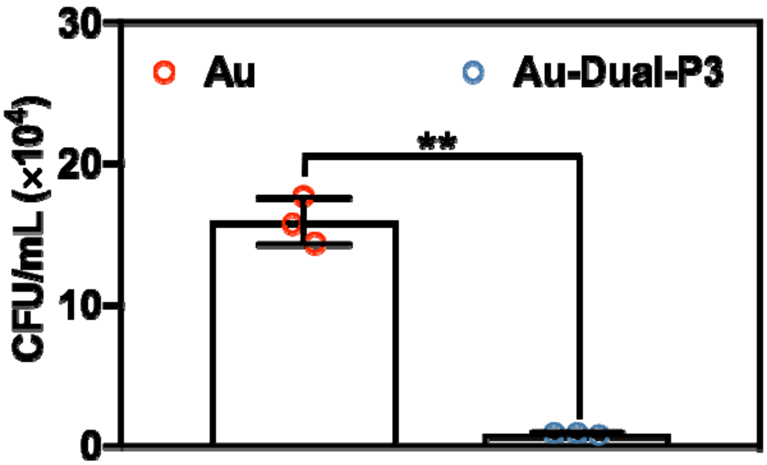

Supplementary Fig. 58 | Antimicrobial assay of the indicated uniform Au surfaces against S. aureus by an agar plate method (n = 3). (Sidak's multiple comparisons test, two-way ANOVA. **p < 0.0001). Data are displayed as mean ± SD. See Experimental section of "Antimicrobial assay" in the Main Text for details of the assay (** denotes $p < 0.01$).

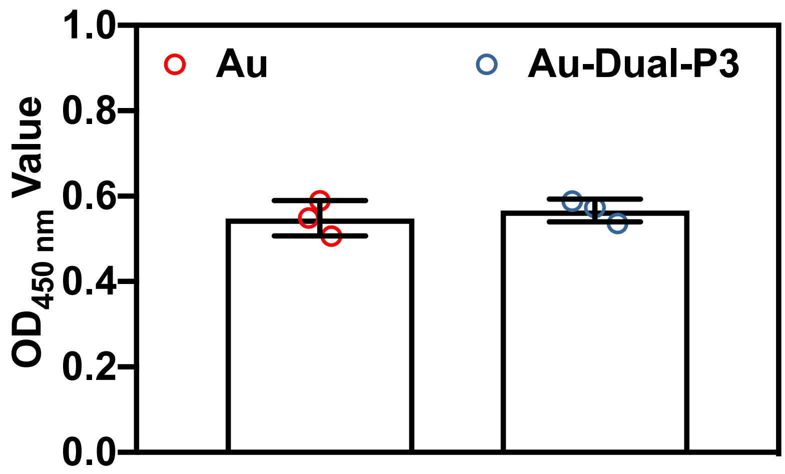

Supplementary Fig. 59 | CCK-8 results for the indicated uniform Au surfaces with *mBMSCs* after 3 days of culturing (n = 3). See Experimental section of "Cell assay" in the Main Text for details of the assay.

Supplementary Table 1 | The sequence of peptides.

Peptides	Sequence
AMP	Cys-Pro-Ala-Pro-Ala-Pro-Lys-Arg-Trp-Trp-Lys-Trp-Trp-Arg-Arg
RGD	Cys-Pro-Ala-Pro-Ala-Pro-Arg-Gly-Asp
AMP-FITC	Cys-Pro-Ala-Pro-Ala-Pro-Lys-Arg-Trp-Trp-Lys-Trp-Trp-Arg-Arg-Lys -FITC
RGD-FITC	Cys-Pro-Ala-Pro-Ala-Pro-Arg-Gly-Asp-Lys-FITC
RGD-Mca	Cys-Pro-Ala-Pro-Ala-Pro-Arg-Gly-Asp-Lys-Mca

Supplementary Table 2 | The densities of AMP and RGD on the indicated uniform Ti surfaces.

Sample	Density of AMP (ng/cm^2)	Density of RGD (ng/cm^2)
Ti-Dual-P1	238.1 ± 16.0	31.2 ± 1.2
Ti-Dual-P2	274.7 ± 21.9	29.9 ± 0.6
Ti-Dual-P3	305.4 ± 31.2	28.4 ± 0.8
Ti-Dual-P4	338.2 ± 26.2	27.8 ± 0.4

Supplementary References:

1. Wang, J., Wang, W., Kollman, P.A. & Case, D.A. Automatic atom type and bond type perception in molecular mechanical calculations. *Journal of Molecular Graphics and Modelling* **25**, 247-260 (2006).
2. Wang, J., Wolf, R.M., Caldwell, J.W., Kollman, P.A. & Case, D.A. Development and testing of a general amber force field. *Journal of Computational Chemistry* **25**, 1157-1174 (2004).
3. Sousa da Silva, A.W. & Vranken, W.F. ACPYPE - AnteChamber PYthon Parser interfacE. *BMC Research Notes* **5**, 367 (2012).
4. Schrodinger, LLC (2015).
5. Hinterwirth, H. et al. Quantifying Thiol Ligand Density of Self-Assembled Monolayers on Gold Nanoparticles by Inductively Coupled Plasma–Mass Spectrometry. *ACS Nano* **7**, 1129-1136 (2013).
6. Zheng, J. et al. Controlling the Integration of Polyvinylpyrrolidone onto Substrate by Quartz Crystal Microbalance with Dissipation To Achieve Excellent Protein Resistance and Detoxification. *ACS Applied Materials & Interfaces* **8**, 18684-18692 (2016).
7. Jeschke, B. et al. RGD-peptides for tissue engineering of articular cartilage. *Biomaterials* **23**, 3455-3463 (2002).
8. Wang, L. et al. Mechanistic Insights and Rational Design of a Versatile Surface with Cells/Bacteria Recognition Capability via Orientated Fusion Peptides. *Advanced Science* **6**, 1801827 (2019).
