# Supplementary material for: High-throughput screening and rational design of biofunctionalized surfaces with optimized biocompatibility and antimicrobial activity
Source: Nat Commun. 2021 Jun 18;12:3757. doi: 10.1038/s41467-021-23954-8 (PMC8213795; doi:10.1038/s41467-021-23954-8)
Supplement: Supplementary file 2 — Reporting Summary [file 41467_2021_23954_MOESM2_ESM.pdf]

nature research

Corresponding author(s): Lin Wang, Xuetao Shi, Yingjun Wang, Chuanbin Mao

Last updated by author(s): Jul 8, 2020

# Reporting Summary

Nature Research wishes to improve the reproducibility of the work that we publish. This form provides structure for consistency and transparency in reporting. For further information on Nature Research policies, seeAuthors & Referees and theEditorial Policy Checklist .

## Statistics

For all statistical analyses, confirm that the following items are present in the figure legend, table legend, main text, or Methods section.

| n/a | Confirmed | |
|---|---|---|
| ☐ | ☒ | The exact sample size (*n*) for each experimental group/condition, given as a discrete number and unit of measurement |
| ☐ | ☒ | A statement on whether measurements were taken from distinct samples or whether the same sample was measured repeatedly |
| ☐ | ☒ | The statistical test(s) used AND whether they are one- or two-sided<br>*Only common tests should be described solely by name; describe more complex techniques in the Methods section.* |
| ☒ | ☐ | A description of all covariates tested |
| ☐ | ☒ | A description of any assumptions or corrections, such as tests of normality and adjustment for multiple comparisons |
| ☐ | ☒ | A full description of the statistical parameters including central tendency (e.g. means) or other basic estimates (e.g. regression coefficient) AND variation (e.g. standard deviation) or associated estimates of uncertainty (e.g. confidence intervals) |
| ☐ | ☒ | For null hypothesis testing, the test statistic (e.g. *F*, *t*, *r*) with confidence intervals, effect sizes, degrees of freedom and *P* value noted<br>*Give P values as exact values whenever suitable.* |
| ☒ | ☐ | For Bayesian analysis, information on the choice of priors and Markov chain Monte Carlo settings |
| ☒ | ☐ | For hierarchical and complex designs, identification of the appropriate level for tests and full reporting of outcomes |
| ☒ | ☐ | Estimates of effect sizes (e.g. Cohen's *d*, Pearson's *r*), indicating how they were calculated |

*Our web collection on statistics for biologists contains articles on many of the points above.*

## Software and code

Policy information about availability of computer code

| | |
|---|---|
| Data collection | The MFI of the surface was obtained via the fluorescence microscopy (Eclipse Ti-U, Nikon); the cells were characterized with the Eclipse Ti-U instrument (Nikon, Japan); The H&E, immunohistochemistry staining methylene blue & basic fuchsin and toluidine blue tissue sections were obtained via the Eclipse Ti-U instrument under the bright field channels (Nikon, Japan); The Au electrode was tested with QCM-D (Q-Sense AB, Sweden); The samples of XPS assay was performed on the photoelectron spectrometer with the X-ray source of Al Kα (1486.4 eV) (AXIS ULTR DLD, Kratos, England). The samples of AFM assay was performed by a MultiMode Nanoscope IIIa AFM (Digital Instruments Inc., Santa Barbara, CA).The samples of FTIR assay was performed on Vertex 70 (Bruker, Germany). The MD simulation results were collected by “gmx_sasa” program in GROMACS 5.1.4, ACPYPE 0.1.1 and PyOML 2.4.0 |
| Data analysis | SPSS 17.0 and Graphpad prism 8.0 were used for the generation of graphs and data analysis. Image J 2.1.0 software (National Institutes of Health, USA) was used for the quantization of image. The statistical significance of observed differences was analyzed by ANOVA test. |

For manuscripts utilizing custom algorithms or software that are central to the research but not yet described in published literature, software must be made available to editors/reviewers. We strongly encourage code deposition in a community repository (e.g. GitHub). See the Nature Research guidelines for submitting code & software for further information.

## Data

Policy information about availability of data

All manuscripts must include a data availability statement. This statement should provide the following information, where applicable:
- Accession codes, unique identifiers, or web links for publicly available datasets
- A list of figures that have associated raw data
- A description of any restrictions on data availability

All data are reported in this manuscript and supporting information and are also available from the authors upon request.

# Field-specific reporting

Please select the one below that is the best fit for your research. If you are not sure, read the appropriate sections before making your selection.

☒ Life sciences ☐ Behavioural & social sciences ☐ Ecological, evolutionary & environmental sciences

For a reference copy of the document with all sections, see nature.com/documents/nr-reporting-summary-flat.pdf

# Life sciences study design

All studies must disclose on these points even when the disclosure is negative.

| | |
|---|---|
| Sample size | All the experiments were carried out in three replicates or more based on our previous experience. |
| Data exclusions | No data exclusions were made. |
| Replication | We found that the data could be replicated. |
| Randomization | The animals were randomly allocated into experimental groups for analysis |
| Blinding | One person implanted different implants into different animal groups while other persons were blinded to group allocation during data collection and/or analysis. |

# Reporting for specific materials, systems and methods

We require information from authors about some types of materials, experimental systems and methods used in many studies. Here, indicate whether each material, system or method listed is relevant to your study. If you are not sure if a list item applies to your research, read the appropriate section before selecting a response.

## Materials & experimental systems

| n/a | Involved in the study | |
|---|---|---|
| ☐ | ☒ | Antibodies |
| ☐ | ☒ | Eukaryotic cell lines |
| ☒ | ☐ | Palaeontology |
| ☐ | ☒ | Animals and other organisms |
| ☒ | ☐ | Human research participants |
| ☒ | ☐ | Clinical data |

## Methods

| n/a | Involved in the study | |
|---|---|---|
| ☒ | ☐ | ChIP-seq |
| ☒ | ☐ | Flow cytometry |
| ☒ | ☐ | MRI-based neuroimaging |

## Antibodies

| | |
|---|---|
| Antibodies used | The following antibodies were used for IHC: IgG H&L (HRP) (abcm, ab275023), Anti-protein A (abcm, 181627), each antibody was diluted 1000 times. |
| Validation | All the antibodies were authenticated by the manufacturer. |

## Eukaryotic cell lines

Policy information about cell lines

| | |
|---|---|
| Cell line source(s) | The mBMSCs were purchased in VWR International, LLC, Pennsylvania, USA (ATCC CRL-12424) |
| Authentication | The mBMSCs were authenticated by VWR International, LLC, Pennsylvania, USA |
| Mycoplasma contamination | The mBMSCs were regularly monitored for mycoplasma contamination. All tests were negative. |
| Commonly misidentified lines (See ICLAC register) | The cell lines we used are not in the ICLAC database |

## Animals and other organisms

Policy information about studies involving animals; ARRIVE guidelines recommended for reporting animal research

| | |
|---|---|
| Laboratory animals | New Zealand rabbits (6-month-old, male) were raised in a standard clean environment. |
| Wild animals | This study did not involve wild animals. |
| Field-collected samples | This study did not involve field-collected samples. |
| Ethics oversight | All animal experiments were approved by the Institutional Animal Care and Use Committee of Guangdong Medical Laboratory Animal Center (Foshan, China). |

Note that full information on the approval of the study protocol must also be provided in the manuscript.
